# Spatial encoding in primate hippocampus during free navigation

**DOI:** 10.1371/journal.pbio.3000546

**Published:** 2019-12-09

**Authors:** Hristos S. Courellis, Samuel U. Nummela, Michael Metke, Geoffrey W. Diehl, Robert Bussell, Gert Cauwenberghs, Cory T. Miller

**Affiliations:** 1 Cortical Systems and Behavior Laboratory, University of California, San Diego, San Diego, California, United States of America; 2 Department of Bioengineering, University of California, San Diego, San Diego, California, United States of America; 3 Neurosciences Graduate Program, University of California, San Diego, San Diego, United States of America; 4 Center for Functional MRI, University of California, San Diego, San Diego, United States of America; Institute of Science and Technology Austria, AUSTRIA

## Abstract

The hippocampus comprises two neural signals—place cells and θ oscillations—that contribute to facets of spatial navigation. Although their complementary relationship has been well established in rodents, their respective contributions in the primate brain during free navigation remains unclear. Here, we recorded neural activity in the hippocampus of freely moving marmosets as they naturally explored a spatial environment to more explicitly investigate this issue. We report place cells in marmoset hippocampus during free navigation that exhibit remarkable parallels to analogous neurons in other mammalian species. Although θ oscillations were prevalent in the marmoset hippocampus, the patterns of activity were notably different than in other taxa. This local field potential oscillation occurred in short bouts (approximately .4 s)—rather than continuously—and was neither significantly modulated by locomotion nor consistently coupled to place-cell activity. These findings suggest that the relationship between place-cell activity and θ oscillations in primate hippocampus during free navigation differs substantially from rodents and paint an intriguing comparative picture regarding the neural basis of spatial navigation across mammals.

## Abstract 2

A neurophysiology study of marmoset monkey hippocampus during free navigation identified place cells analogous to those seen in other species, but the limited interaction observed between the activity of these neurons and theta oscillations suggests notable divergence from rodent models.

## Introduction

Neurons in the hippocampus that encode self position during spatial navigation—i.e., place cells—were first discovered in the common rat [1] but have now been reported in several other mammalian species [2, 3]. Research examining similar neurons in the human hippocampus and the nonhuman primate hippocampus has been dominated by experiments in which restrained subjects explore space either by looking at images or exploring virtual space using a joystick [4–14]. Experiments in which the activity of hippocampal neurons was recorded as freely moving primates navigated a spatial environment have been notably limited [7,15,16], but at least one study does provide evidence of place cells in the primate hippocampus during this facet of spatial exploration [16]. A second prevalent neural signal in rodent hippocampus that covaries with several features of spatial encoding is the θ oscillation [17–21]. Although this oscillation in the local field potential factors prominently in models of medial temporal lobe function for rodents [18,19,22], its functional significance for spatial navigation in other mammals is not as clear. Studies of free-flying Egyptian fruit bats, for example, have shown that place cells and grid cells can form in the absence of coupled θ oscillations [3,23]. Although θ oscillations have been described in the human and the nonhuman primate hippocampus [11,24], the relationship between this neural signal and place-cell activity during free navigation is indeterminate in our phylogenetic order.

Evidence suggests that—similar to rodents—θ oscillations may be correlated with at least some features of spatial navigation in human and nonhuman primates. A series of electroencephalogram (EEG) studies in humans, for example, has shown that θ is modulated during locomotion while freely navigating space [24,25]. However, this neurophysiological approach lacks the resolution to precisely elucidate dynamic interactions that might occur between θ and single-neuron responses, thereby limiting how directly these data can be compared to analogous nonhuman animal studies. Single-unit neurophysiology experiments in head-restrained macaque monkeys may address at least some of this concern. These experiments revealed not only a prevalent θ oscillation in primate hippocampus but one that occurs in sporadic bouts rather than continuously [11]. Further analyses showed that monkey saccadic eye movements reset the phase of hippocampus θ oscillations, suggesting a key interaction between a visual exploration behavior and this neural signal [11,26]. Whether further dynamic interactions would be evident during other exploratory behaviors, such as free navigation, is not known because of the lack of experiments in primates. Here, we recorded the activity of single neurons in the hippocampus of freely moving common marmoset monkeys (*Callithrix jacchus*) as they actively explored real-world environments. We tested whether neurons that encode temporally stable and spatially precise place fields and θ oscillations are present in primate hippocampus during free navigation and whether or not there is significant phase coding between the two. This New World monkey has emerged as a powerful model organism in neuroscience [27,28] and has several advantages for studies of spatial navigation. Because of their small body size, marmosets are particularly amenable to techniques for recording single-neuron activity in the context of free, unrestrained exploration [29–32]. The marmoset brain in general [33,34]—and hippocampus more specifically [35,36]—exhibits the core anatomical organization shared across primates, thus allowing for more direct comparisons with other primates, including humans. Moreover, marmosets exhibit patterns of hippocampal-dependent memory consistent with other primates, including humans [37,38]. By leveraging these attributes, we report clear evidence of place cells in a primate hippocampus that encode self position during natural exploration of space and evidence that θ oscillations are only weakly coupled to the activity of these neurons and the exploratory behavior of these primates.

## Results

### Neurons in the hippocampus-encoded space during free navigation

**Fig 1:**
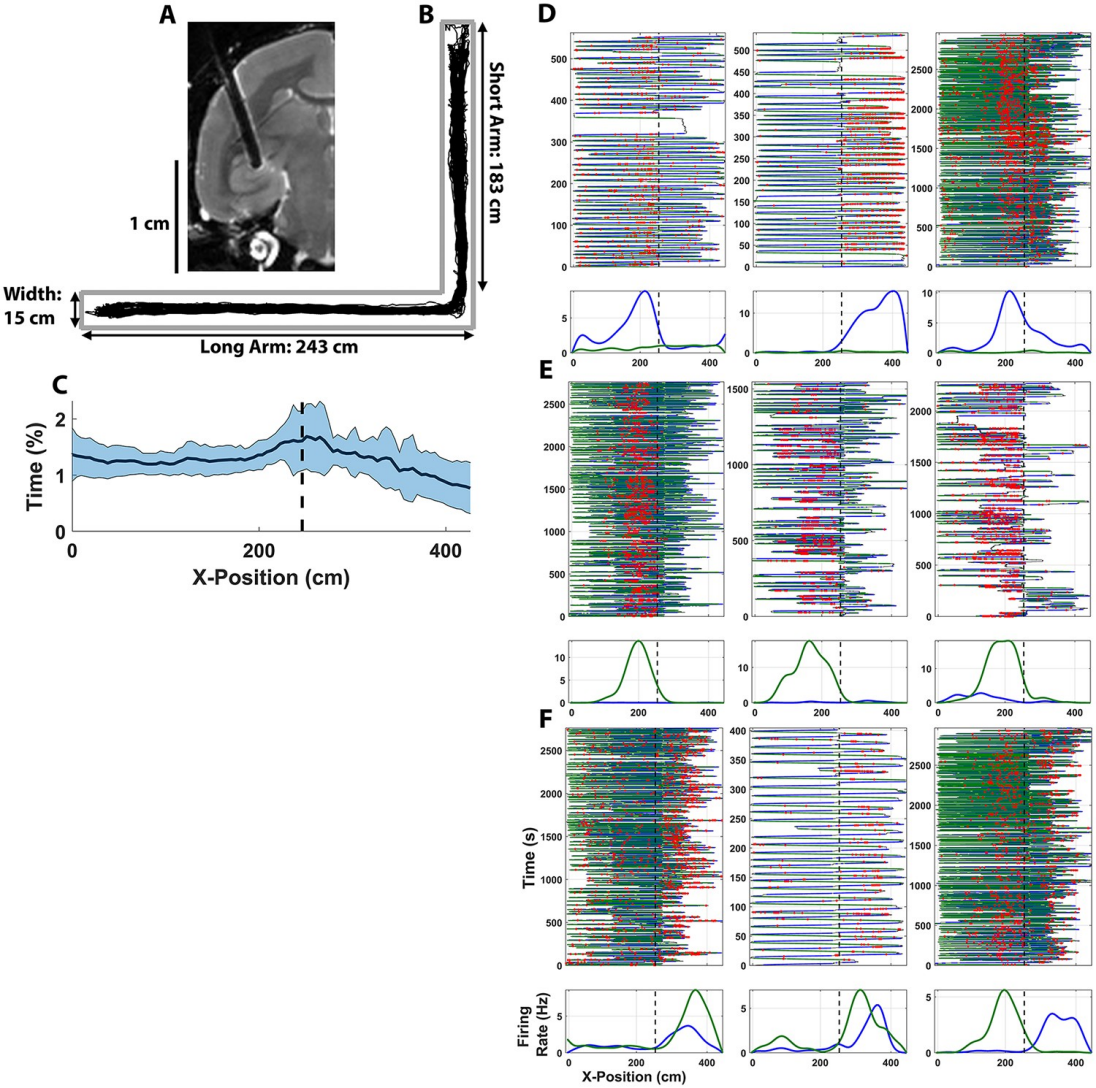
Place cells in the hippocampus of freely moving marmosets. (A) Chronic multi-electrode arrays were implanted directly into the body of the hippocampus using a T2-weighted sMRI-guided surgical procedure. (B) Exemplar behavioral recording of marmoset locomotion along the L track is shown in black within the test environment. (C) Plots of a linearized mean (± σ) occupancy time for positions across the track for all recordings in this test environment. Black line indicates the mean percent of time at each position in the track, and blue indicates 2 SEMs. The vertical dashed line plots the 90° turn in the L track. (D–F) Plots of exemplar place cells in the L-track environment. Three representative neurons are shown for (D) RSPF, (E) LSPF, and (F) bidirectionally tuned place cells from this population. For each individual neuron, the top plot shows individual travel trajectories for each test session (x-axis plots the position on the track, whereas the y-axis plots time [s]). The green line indicates left-moving travel whereas blue lines indicate right-moving travel. Red dots plot the occurrence of an action potential during locomotion. Bottom plots are histograms of the spatial position of action potentials for each neuron, distinguishing between left-moving (green) and right-moving (red) travel during HV movement (>20 cm/sec). The vertical dashed line plots the 90° turn in the L track. The underlying data can be found at https://doi.org/10.5061/dryad.kk63d49. HV, high velocity; LSPF, left-selective place field; RSPF, right-selective place field.

**Fig 2:**
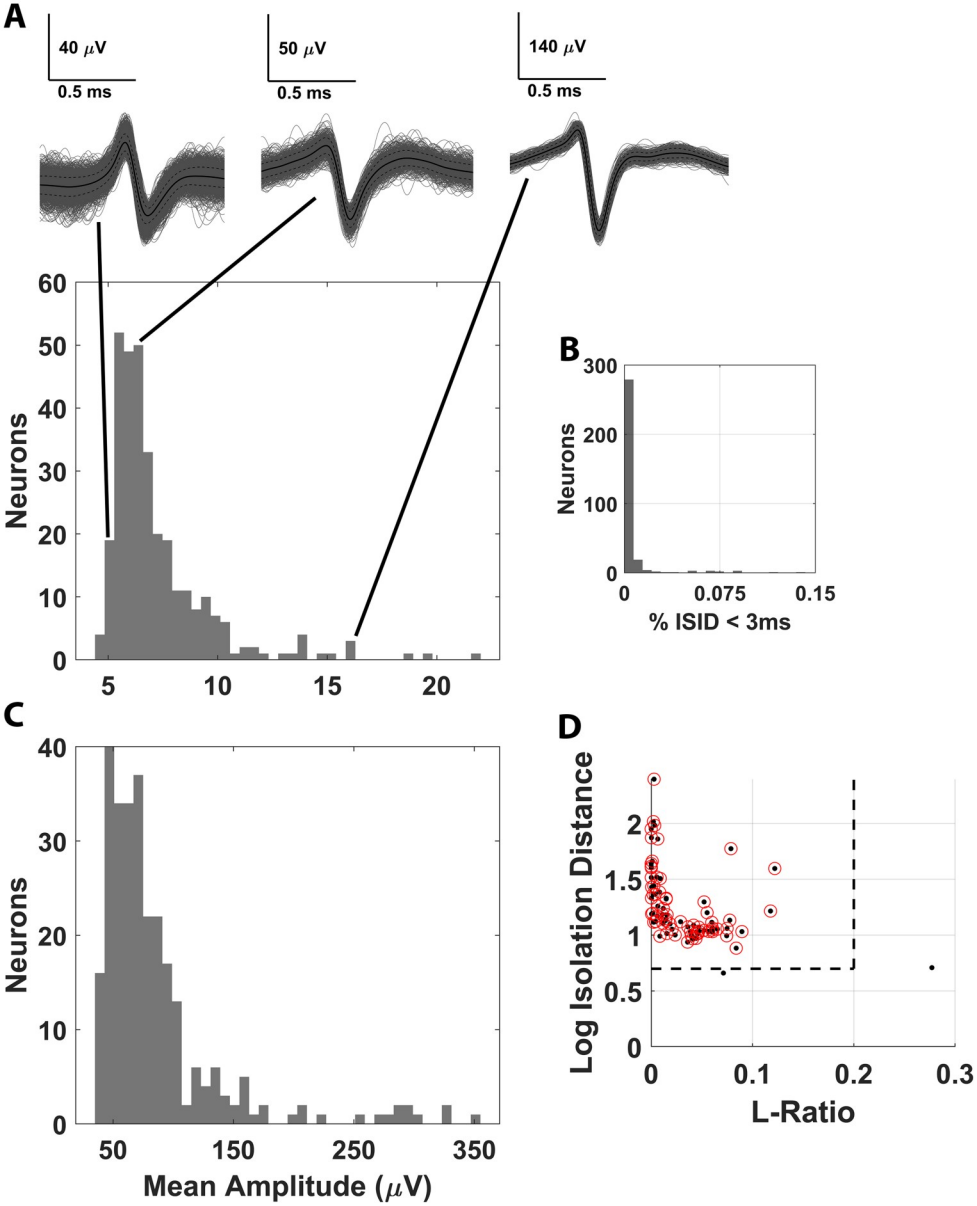
Cluster-quality analyses for all clusters considered single neurons in downstream analyses. (A) The SNR distribution for all single neurons is shown with randomly selected exemplar units showing relatively low SNR (5 dB), distribution-median SNR (6.5 dB), and high SNR (16 dB). (B) The proportion of interspike intervals violating refractoriness (<3 ms) was also computed for each well-isolated neurons, and the distribution is shown as a percent of the total number of interspike intervals. (C) The distribution of mean waveform amplitude (peak to trough) is also shown for all recorded place cells. (D) The distribution of L ratio and ID is also shown for well-isolated neurons, with lower bound thresholds on quality for both metrics overlaid. Each black dot represents the (L ratio, Base 10 log ID) pair for a single neuron, with neurons lying to the left of the L ratio threshold and above the log ID threshold; the vertical and horizontal dotted lines, respectively, are circled in red. Neurons not circled in red were candidate place cells that were not included in subsequent analysis. The underlying data can be found at https://doi.org/10.5061/dryad.kk63d49. ID, isolation distance; ISID, interspike interval distribution; SNR, signal to noise ratio.

To initially probe for the existence of spatial encoding in the marmoset hippocampus, we recorded the activity of single hippocampal neurons as a subject freely explored a 2.44-m long linear track in uniform lighting. These experiments confirmed the presence of neurons that exhibit spatial selectivity during high-velocity (HV) locomotion in a simple linear environment (S1 Fig). Driven by these initial findings, we sought to more systematically characterize the firing dynamics of these neurons by conducting subsequent recordings in a larger environment commonly used in rodent research. An L-track was constructed in which a 1.83-m arm was oriented at a right angle at the end of a 2.43-m arm, thus giving the subject a total of 4.26 m of track to explore. This L track was advantageous in that it provided a highly salient physical landmark and context switch by rounding the corner from one arm to the other as well as providing a large linear distance over which place fields could form. Extensive recordings were conducted in this environment using 2 marmoset monkeys that were implanted with 64-channel Microwire Brush Arrays (MBAs). One subject had a single MBA implanted in anteromedial hippocampus (Fig 1A), whereas the second subject was implanted bilaterally with progressively more posterior-hippocampal implants, yielding a total of 3 hemispheres across the 2 marmosets (S2 Fig and S1 Table). By estimating the maximal volume of coverage for each implanted MBA, it was determined that neurons were sampled from the dentate gyrus, Cornu Ammonis 3 (CA3), and CA1 (S3 Fig and S2 Table). Because the specific location of each microwire—as well as any given unit—within that maximal space recorded by each MBA cannot be determined, no attempts were made to differentiate neurons from each of these regions. Behaviorally, subjects explored the environment frequently and completely without exhibiting significant behavioral preferences for either arm of the track (Fig 1B and 1C, and S4 Fig). Single- and multi-unit spiking activity was isolated from continuous neural recording through standard spike-sorting techniques, and only well-isolated single units, as determined by a number of cluster-quality analysis techniques (Fig 2 and S5 Fig), were considered putative neurons to be included in subsequent analysis. The spatial tuning of each neuron was analyzed by projecting subject movement during recording onto the principal axis of the track and computing 1D firing rate of each neuron as a function of subject position along the track. Only spikes discharged while the subject was locomoting at a velocity greater than 20 cm/s were included for subsequent place-field calculation. This velocity threshold was selected because it was able to isolate the ballistic trajectories that marmosets tended to make across the track (examples in Fig 1D–1F) when roaming around the environment, while excluding short periods of acceleration and deceleration from the place field calculation at the end of such trajectories. The statistical significance of the spatial information encoded by each neuron was evaluated using a common circular-shift shuffling technique (*N* = 1,000) [23]. Neurons were only labeled as “place cells” if they exhibited significant spatial selectivity at *p* < 5 × 10^−3^. Although our criterion is more conservative than what is typical of analogous studies involving rodents, this threshold was implemented to ensure that only neurons exhibiting highly significant spatial selectivity were included in our analyses. Furthermore, no lower bound was set a priori on the magnitude of the spatial information exhibited by candidate place cells. This approach was used intentionally to prevent rejection of place cells that encoded significant information regarding self position on the track but did so with great spatial extent. However, absolute rejection thresholds were set using the L ratio and isolation distance (ID) by setting a lower bound on a previously described classification scheme [39]. Two candidate place cells were rejected at this stage for having low cluster quality in spite of their significant place tuning. It is noted that, per the classification scheme proposed in the work by Ainge and colleagues [39], 48 candidate units would be classified as “poor” by ID (<20) but would be “good” by L Ratio (<0.1) and thus were retained for subsequent analysis. Using these criteria, we classified 289 neurons out of the 2,054 single units and multi-units recorded in the marmoset hippocampus (14.1%) as place cells.

Place cells were isolated from 37 recording sessions across the 3 implanted hemispheres, with an average yield of 7.6 ± 4.8 place cells per session. These neurons varied considerably in their information content on the track, with a mean of 1.60 ± 1.61 bits/spike of encoded self-position information (information rate: 0.97 ± 0.61 bits/s). The full pseudopopulation distributions across place cells in all recordings are shown in S6 Fig, including the distribution for mean firing rate, spatial information rate, and spatial information. Consistent with the rodent literature [40, 41], a number of different place-encoding dynamics were observed. Of the neurons that exhibited strong spatial selectivity, 77.9% (225/289) revealed strong place encoding in one direction of travel and not in the other. Within this group, 40.4% (91/225) of neurons had only a right-selective place field (RSPF; Fig 1D and S7A Fig), and 59.6% (134/225) of neurons had only a left-selective place field (LSPF; Fig 1E and S7B Fig). The remaining 64 neurons (22.1%) exhibited significant place tuning in both directions (i.e., bidirectional tuning; Fig 1F and S7C Fig), with the correlation between their RSPFs and LSPFs significantly deviating from a uniform distribution over the interval they covered (*p* = 8 × 10^−6^, Kolmogorov-Smirnov Test [KS]) and skewed toward encoding similar track positions rather than being uncorrelated. This observation is consistent with previous findings demonstrating increased overlap in place fields of bidirectional place cells on a linear track in the context of a cue-rich environment such as our own [42].

**Fig 3:**
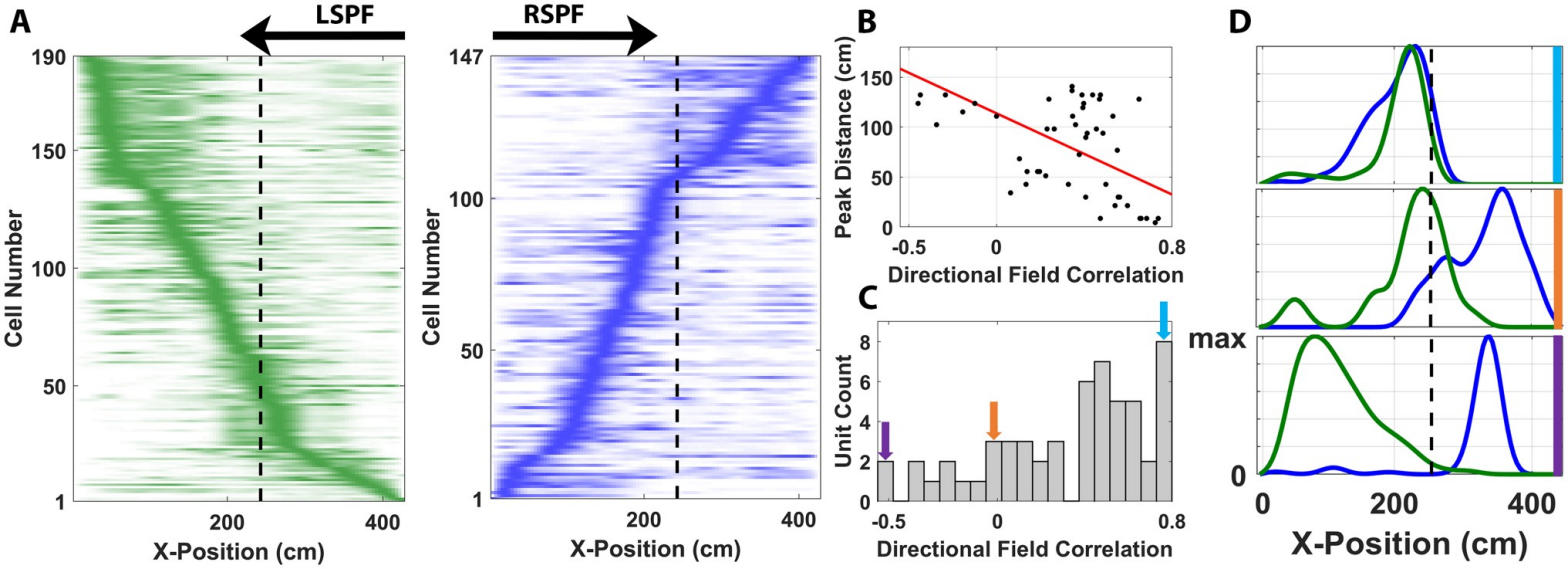
Population dynamics of place encoding. (A) LSPFs (left) and RSPFs (right) from all recorded place cells, linearized along the principal axis of the L track, normalized to maximum firing rate and sorted by position along the track according to ascending displacement from point of origin. The vertical dashed line demarks the transition point between the long and short arms of the L track. (B) The scatter plot shows the relationship between peak distance separation of the place field in each direction (right and left) across the track and directional field correlation. Each black dot shows an individual bidirectionally tuned neuron, whereas the red line plots the least-square fit line. (C) The distribution of directional field correlations for bidirectional units is shown. (D) Plots of exemplar bidirections place cells exhibiting high (top; blue), middle (orange), and low (bottom; purple) correlations, which correspond to the arrows shown in panel C. The firing rate of these exemplar neurons at each position along the L track is shown during left-moving (green) and right-moving (blue) travel. Place fields shown in panel D are all normalized to maximum firing rate. The underlying data can be found at https://doi.org/10.5061/dryad.kk63d49. LSPF, left-selective place field; RSPF, right-selective place field.

The fields from all recorded place cells were separated according to direction of travel, normalized by maximum firing rate, and sorted by peak firing rate location in their respective direction. The accumulated fields demonstrated complete tiling of the explored environment in both directions (Fig 3A). In fact, on some recording days with higher yield, a population of place cells was recorded that achieved coverage of the entirety of the explored environment (S8 Fig). This suggests that the population of place cells in the marmoset hippocampus encoded a full internal representation of the local environment during locomotion through the space as has previously been observed extensively in other mammals [43,44]. Interestingly, a slope-asymmetry was present in both the LSPF and RSPF population plots, indicating that the short arm of the track, which had the shallower slope, was being tiled by fewer place cells in both directions. A number of mechanisms might have contributed to this representational difference, including the increased optic flow that would have been associated with faster traversals of the long arm (S4B Fig), or simply an increased salience of the long arm of the L track when compared with the short arm. Increasing environmental size has been associated with increased place-cell recruitment in rodents [45], and the asymmetry seen here might be a reflection of this phenomenon in the marmoset hippocampus.

We further analyzed neurons exhibiting bidirectional tuning and, as an internal control, evaluated the relationship between peak place-field location and place-field correlation. Analyses confirmed that the distribution of correlations was related to the distance between the peaks of the LSPF and RSPF (*ρ* = −0.56, *R*^*2*^ = 0.31; Fig 3B) and was not significantly related to differences in information encoding between the two directions or changes in place-field shape independent of position encoding. These neurons were biased toward exhibiting strong correlations in bidirectional tuning, indicating that most of these cells encoded similar locations in space when traveling both left and right through the track, though some variation was evident across the population (Fig 3C and 3D).

### Spatial representations were stable over time

**Fig 4:**
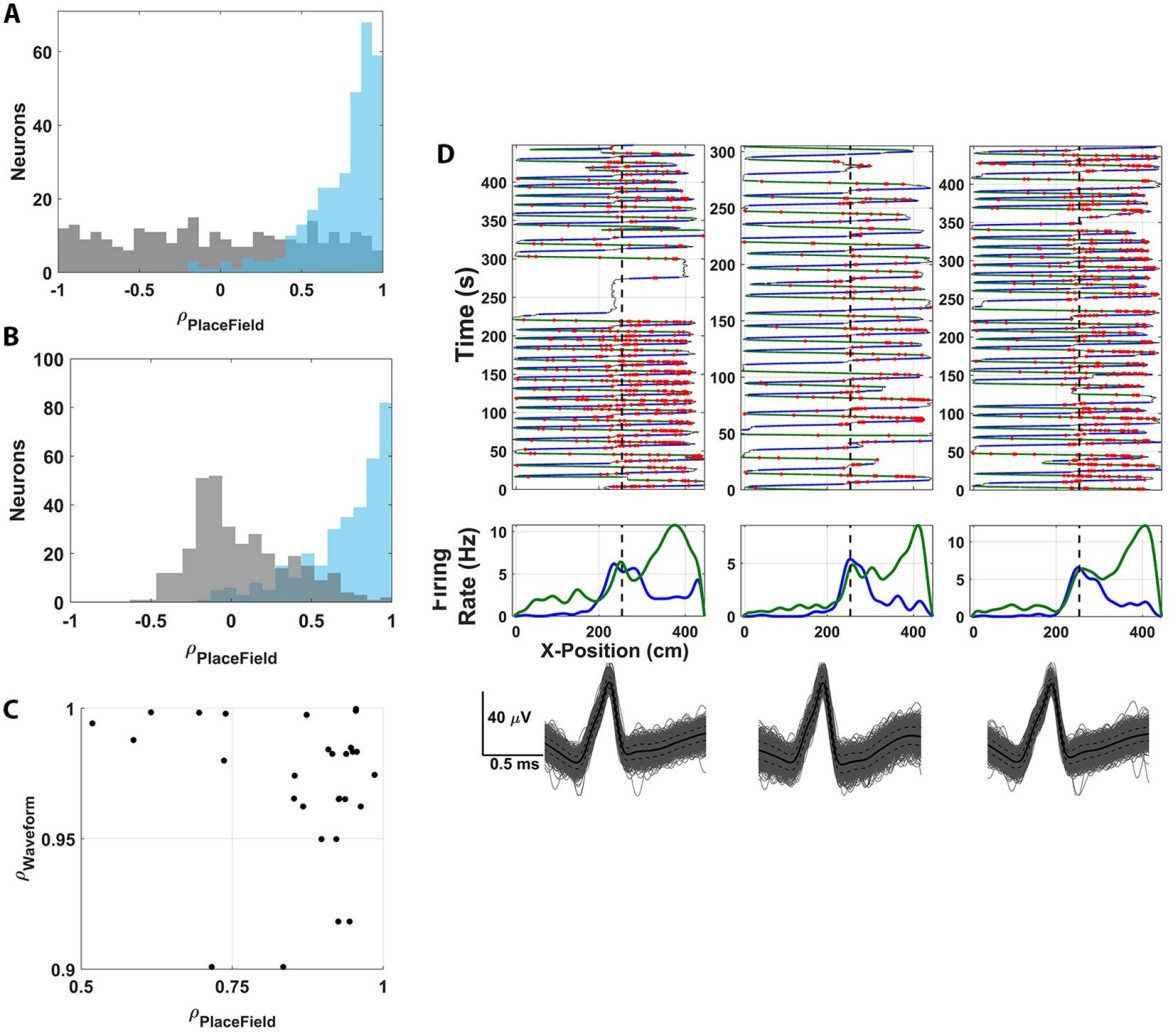
Stability of marmoset place cells. (A) Place-field stability across the population of all recorded neurons was evaluated by correlating fields computed from the first and second halves of recording (light blue) and is significantly right-shifted (*p* < 10^−50^, KS test) compared with the null distribution of correlations determined by recomputing the place field for each recording session following circular shifting (gray). (B) The correlation stability distribution (light blue) was also compared with a null distribution determined by shuffling the identity of the second halves of recordings between place cells (gray) and is significantly right-shifted in this case as well (*p* < 10^−50^, KS test). (C) Plotted in black dots are the place field and waveform correlations for 21 individual place cells recorded in 2 separate sessions on the same day. (D) An exemplar neuron is shown across 3 successive recordings performed on the same day in the L-track environment. For each individual session, the top plot shows individual travel trajectories for the test session. The green line indicates left-moving travel whereas the blue lines indicate right-moving travel. Red dots plot the occurrence of an action potential during locomotion. Middle plots show a histogram of the spatial position of action potentials for each neuron, distinguishing between left-moving (green) and right-moving (red) travel during HV movement (>20 cm/s). The vertical dashed line plots the 90° turn in the L track. Bottom plots are the raw waveforms of the cell for each of the sessions. The black solid line shows the average waveform, whereas the dashed black line plots 2 SEMs. The underlying data can be found at https://doi.org/10.5061/dryad.kk63d49. HV, high velocity; KS, Kolmogorov-Smirnov Test.

A key feature of place cells in rodents is that the spatial representations are stable over periods of time. We assessed this characteristic of marmoset place cells in two ways. We first quantified field stability by correlating the place field computed from the first and second half of each test session. This analysis revealed a highly significantly right-shifted correlation distribution across the population of recorded neurons with a mean stability correlation of 0.74 ± 0.23, indicating that the spatial encoding properties of individual marmoset place cells were generally highly stable over the duration of a recording session (*p*-values practically zero, *p <* 10^−55^ *t* test, *p <* 10^−50^ KS-Test with respect to null; Fig 4A). The null distribution in this case was constructed by recomputing the inter-recording place-field correlation for each place cell following spike-position circular shifting as previously described. To further test the significance of the temporal stability observed through the correlation distribution, another hypothesis test was performed with a different null hypothesis that was computed by shuffling the cell identity labels on the second half of the recording and recomputing the correlation for each cell in the population. Again, the correlation distribution was found to be highly significantly right shifted compared with null (*p <* 10^−100^ *t* test, *p <* 10^−50^ KS test with respect to null; Fig 4B). The outcome of this analysis indicates that place cells in marmosets exhibit a key stability characteristic of place cells reported in other mammals (i.e., that these neurons will encode the same position in space when returning to that location at different points in time).

To further characterize the ability of these place cells to stably represent spatial information, we performed an experiment in which we recorded from hippocampal neurons as marmosets explored the same L track in 2 to 3 successive sessions punctuated by the subject’s removal from the environment and transfer to an adjacent room for 10 min. We recorded 21 well-isolated single neurons in this paradigm and used their high waveform stability between subsequent recordings (*ρ*_wav_ = 0.97 ± 0.03) to identify high correlations in their respective place fields in both directions of travel (*ρ*_LSPF_ = 0.85 ± 0.14, *ρ*_RSPF_ = 0.86 ± 0.11; Fig 4C). The exemplar neuron shown in Fig 4D demonstrates the consistency of neural activity across 3 successive recording sessions. This inter-recording stability demonstrates that a given place representation in a marmoset hippocampal neuron associated with an environmental context persists even if the monkey is temporarily removed from that context. In other words, the spatial encoding characteristics of individual neurons retain the same spatial selectivity upon reintroduction to an environment—a characteristic that has been observed in other mammals with place cells over short periods of time (i.e., within the same recording day) [46,47]. The stability of the spatial representation for individual units in marmoset hippocampus further supports classifying these neurons as place cells.

### Spatial information encoding varied along the principal axis of the hippocampus

**Fig 5:**
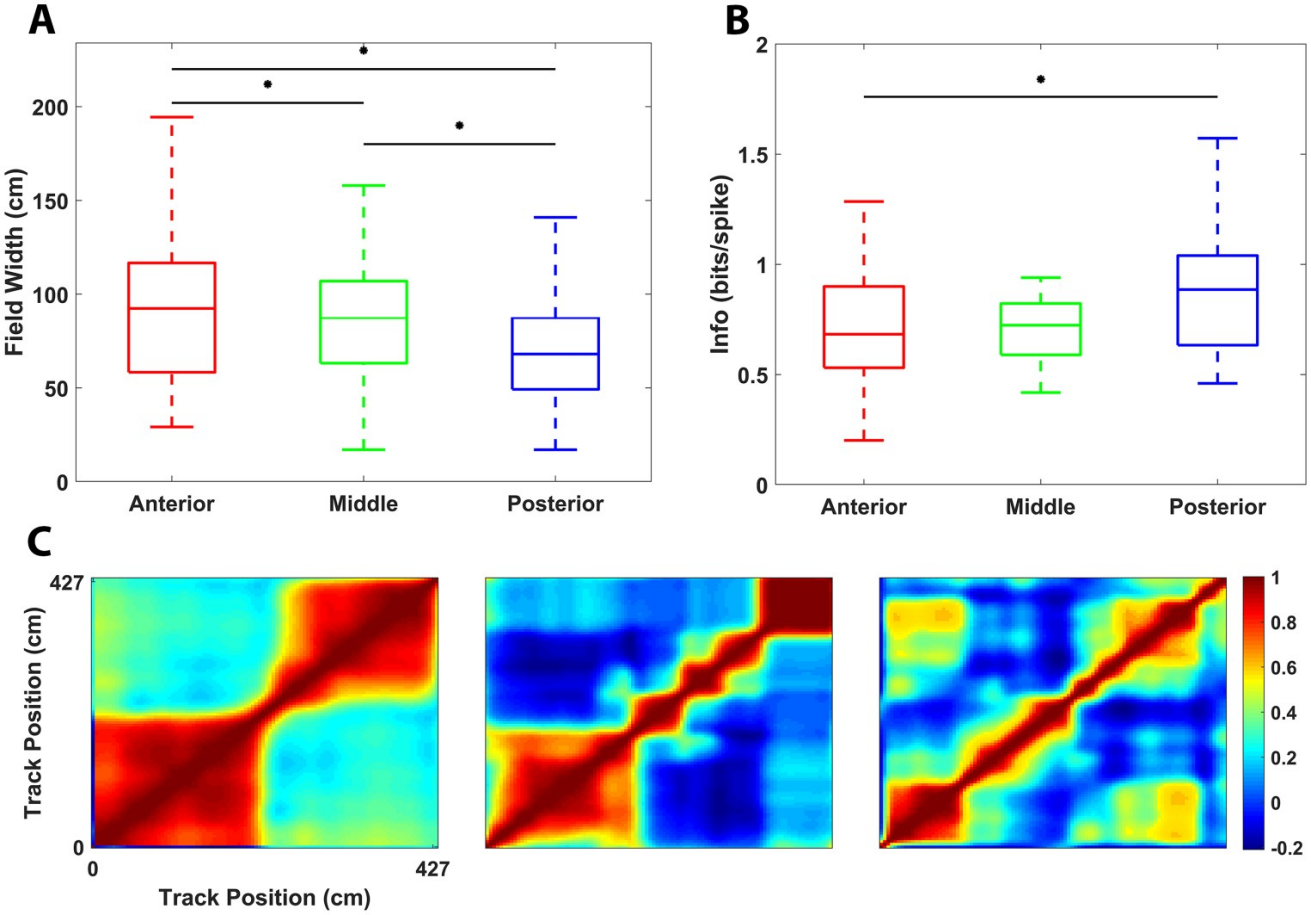
Differences in place field-width and information encoding along the hippocampal long axis. (A) Place-field width defined as 20% of max firing rate and (B) spatial information in bits/spike are displayed using 25-50-75 percentile box plots with 10/90 percentile whiskers. Each region—anterior, middle, posterior—was computed separately. Significant differences are indicated with the starred horizontal black bars above the associated box plot pairs. (C) Population-vector autocorrelation analysis is shown for each of the 3 regions—anterior, middle, and posterior—ordered from left to right. Each point (*i*, *j*) in each matrix corresponds to the Pearson’s correlation coefficient between the activity vector constructed from all place-field firing rates at position *i* on the track with the activity vector of all place field firing rates at position *j* on the track. The color scale shown on the right applies to all 3 correlation matrices shown. The underlying data can be found at https://doi.org/10.5061/dryad.kk63d49.

Another key feature of the spatial representations present within the hippocampus [48,49] and adjacent entorhinal cortex [6,50] is their consistent size gradient at the single neuron level when mapped along various anatomical axes. Specifically, elements of spatial and cognitive tasks are encoded at progressively larger scales when progressing from dorsal to ventral in rodents, and, as some evidence suggests, from posterior to anterior in primates [49, 51]. By implanting MBAs with significant coverage of the long axis of hippocampus (S9 Fig) and recording place-cell activity throughout (S3 Table), we grossly characterized the scale of spatial information encoded by these neurons along this axis. Analysis of individual place fields along this axis revealed that place-field width significantly decreased the further posterior the recording was conducted (Fig 5A: Mann–Whitney U Test, Anterior-Middle (AM), Middle-Posterior (MP), and Anterior-Posterior (AP), being significant (*p* = 0.0446 AM, *p* = 0.003 MP, and *p* = 1.6 × 10^−7^ AP). Field width was computed as the distance between the first crossings of the place field below 20% of the peak firing rate. Correspondingly, the spatial information encoded by individual place fields increased along this axis (Fig 5B: Mann-Whitney U Test, AP comparison (*p* = 0.59 AM, *p* = 0.30 MP, *p* = 0.013). This finding is consistent with the notion that—with decreasing spatial extent of place fields—discharged spikes would be increasingly more informative about the current location of the marmoset on the track. To demonstrate an increase in spatial extent of place fields independently of the place field width definition, the population-vector autocorrelation approach previously employed to characterize the spatial organization of the dorso-ventral axis of rodent hippocampus [52] was also applied here. To achieve this, population vector of activity at every location *i* on the L track was correlated with the population vector at every other location *j*, thus yielding a 2D (*i*, *j*) matrix of the intrinsic spatial extent of place-field correlation at the population level. A wider band of high correlation along the diagonal indicates a greater spatial extent for the neurons incorporated in that population. Indeed, it was found that—in progressing from posterior to anterior—the width of this diagonal band did increase considerably (Fig 5C), reflecting the increase in spatial extent of the place fields when progressing anteriorly along the long axis. Interestingly, even at the most anterior implant site (Fig 5C, left), the high population correlation was limited by a position consistent with the elbow of the L track, with seemingly distinct subcomponents of the place-cell population being sensitive to the long and short arms of the track as reflected by the bilobed diagonal band. Overall, these findings are notably consistent with prior efforts to characterize physiological variability along the long axis of the primate hippocampus and strongly suggest that the spatiotopically organized changes in the spatial-encoding scale present in rodent hippocampus are preserved to some degree in this nonhuman primate.

### Track position was decoded from place-cell population activity

**Fig 6:**
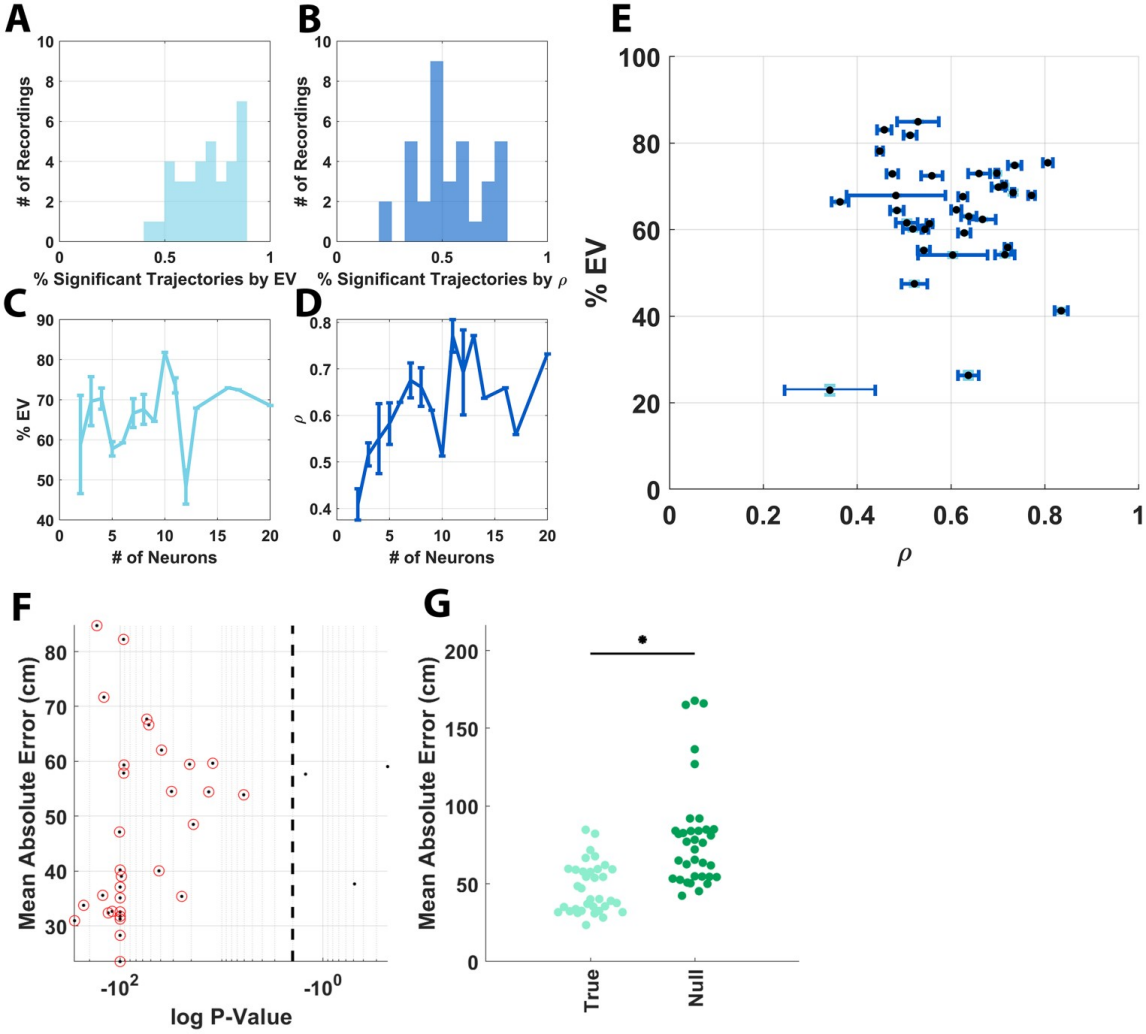
Bayesian decoding of marmoset position from individual recording days. (A) Plots of the number of recordings as a function of the fraction of trajectories per recording whose decoding on average explained a significant amount of variance of the true trajectory. Significance in this case was determined by *p*-value against the null distribution of recomputed decoded trajectories with shuffled place-cell activity. (B) Plots of the same analysis as in panel A but with the correlation of the true trajectory and decoded trajectory as the performance metric of choice. (C) Plots of the percent of variance explained by the decoded trajectories as a function of the number of neurons per recording session. Error bars are SEM computed across all recording days with the given number of neurons. (D) Plots of the same analysis as in panel C with the correlation of the true trajectory and decoded trajectory as the performance metric of choice. (E) The relationship between the mean explained variance and the mean correlation across all trajectories is shown for each recording session. Here, error bars in each dimension are SEM of explained variance and correlation computed across all trajectories for each recording session. (F) The mean absolute error between the decoded and true trajectories is shown for each recording session with a statistical significance threshold given at 0.05 by the vertical dashed line. Here, again, *p*-values were determined by shuffling of place-cell activity followed by recalculation of the mean absolute error. Significant decoding with respect to mean absolute error was found in all but 3 recording sessions (i.e., 34/37 sessions). (G) Plots of the mean absolute error for each recording session with true place fields on the left and the average of the null distribution for each recording on the right. The distributions, here, significantly differ as determined by Mann–Whitney U test (*p* = 1.3 × 10^−5^). This significance is indicated by the horizontal black bar and asterisk plotted above the distributions. The underlying data can be found at https://doi.org/10.5061/dryad.kk63d49.

The relatively large place fields observed in many of the track recordings here invite the question of whether a concurrently recorded population of neurons from the hippocampus in a marmoset is sufficient to decode track position. Substantial efforts have been devoted to the development of various decoding methods that serve to demonstrate both the information content of the recorded neurophysiological signals [53–56] and, when used causally to predict downstream behavior, give insight into the computational mechanisms that the brain employs to subsequently process the recorded neuronal activity [57]. Given observations that concurrently recorded populations of neurons in our subjects appeared to provide full spatial coverage of the environment (Fig 3A), a two-step Bayesian decoder was implemented as described in Zhang and colleagues [53] to decode the position of marmosets during HV trajectories on the L track from concurrently recorded place cells. Statistical significance of trajectory decoding was evaluated by repeatedly decoding marmoset position from shuffled place fields and empirical *p*-value calculation against this null distribution. Post hoc decoding analysis revealed that the decoded trajectories were able to capture a significant fraction of the movement dynamics of individual marmoset trajectories. This was quantified both by the fraction of the variance of each true trajectory that was captured by its respective decoded trajectory (Fig 6A) and by the Pearson’s correlation between the true and decoded trajectories (Fig 6B). The effect of the number of recorded place cells on decoding accuracy was also assessed by evaluating the percentage of explained variance (Fig 6C) and the correlation (Fig 6D) as a function of the number of place cells in the recording session. The positive trend exhibited by these metrics suggests that the increasing number of place cells provided the decoder with improved spatial location information to more accurately infer the marmoset’s position. Overall, the decoded movement trajectories were representative of true marmoset movement, with significant trajectories capturing on average 64.0% ± 13.8% variance (μ ± σ) across all recording sessions with an associated average decoded trajectory correlation of 0.61 ± 0.13 across all recordings. The correlation and explained variance for each recording with associated standard errors computed across all trajectories within each recording from which the above values were computed are shown in Fig 6E. To further quantify the accuracy of the decoder, the mean absolute error between the decoded and true trajectories was computed for each recording session with a statistical significance threshold at 0.05 (Fig 6F). Significant decoding with respect to the mean absolute error was found in all but 3 recording sessions (34/37 sessions). Finally, to demonstrate the magnitude of decoding accuracy with respect to null, the mean absolute error for trajectories decoded with true place fields was compared with the average of the null distribution for each recording (Fig 6G). The distributions, here, significantly differed as determined by Mann–Whitney U test (*p* = 1.3 × 10^−5^). This demonstrates that a significant amount of spatial information contained in the place fields of our recorded place cells was leveraged by the Bayesian decoder to successfully decode marmoset movement on the L track.

### θ oscillations during locomotion

**Fig 7:**
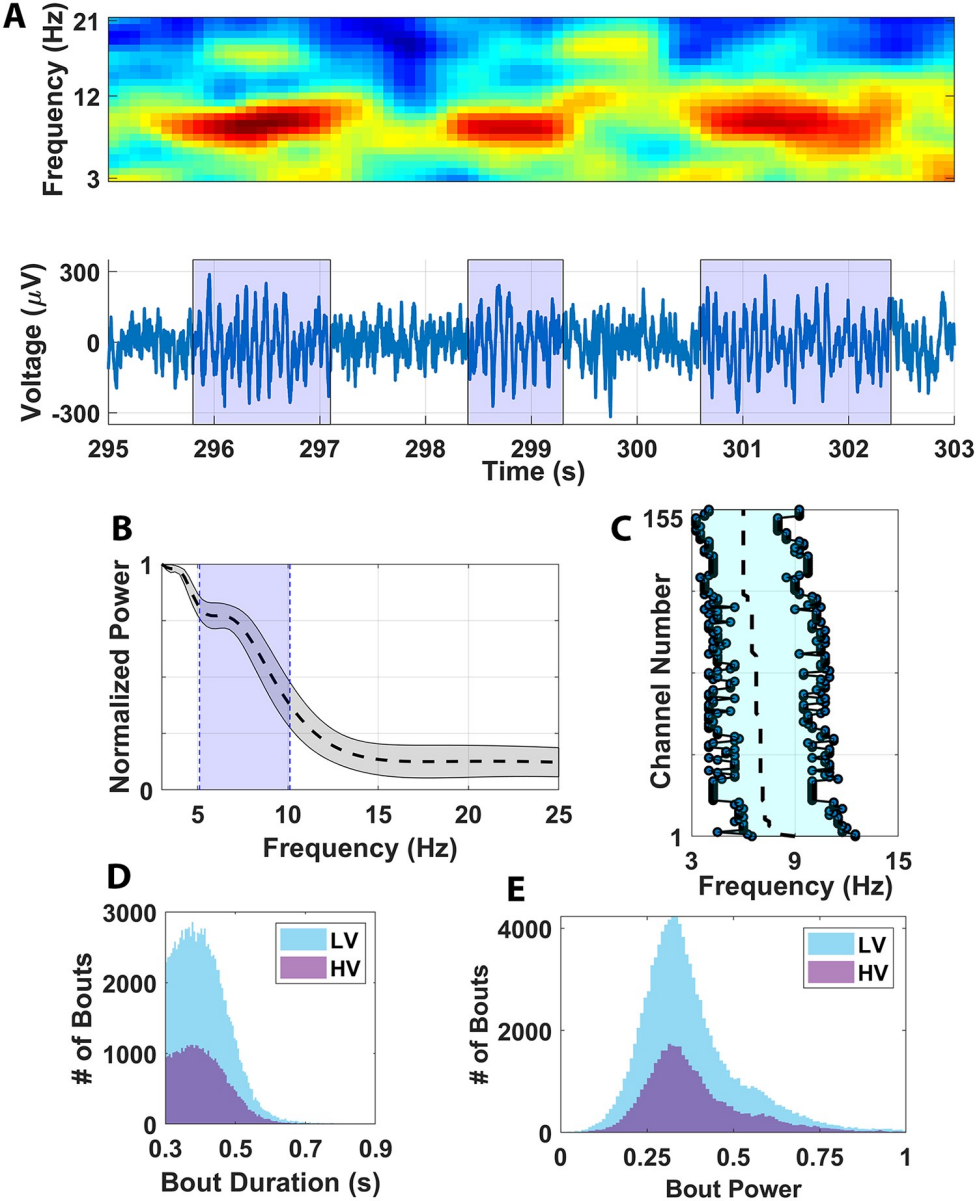
Patterns of θ oscillations in marmoset hippocampus. (A) Above shows a spectrogram highlighting the spectrotemporal dynamics of θ oscillations during locomotion. Below plots the corresponding raw voltage recording from an electrode within the hippocampus during recording. Blue shading reflects the occurrence of θ oscillations. (B) Normalized power spectral across all channels recorded with place-cell activity. Mean power spectra ± SEM is shown by the dashed black line and gray shading, respectively. Blue shading indicates the θ frequency range of 5 to 10 Hz. (C) Plots of the mean (dashed line) and lower and upper limits (light blue shading) of the θ oscillation for each channel recorded with a place cell (i.e., θ bandwidth). The channels have been resorted across all subjects/recordings according to the frequency at which peak θ power was identified. The mean peak across the population was 6.5 Hz. Differences between the number of HV (>20 cm/s; purple shading) versus LV (<20 cm/s, ≥0 cm/s; blue shading) locomotion are plotted for both (D) bout duration and (E) bout power. Neither metric was significantly modulated by locomotor speed when bouts were pooled across all recording sessions (*p* > 0.05, KS test). The underlying data can be found at https://doi.org/10.5061/dryad.kk63d49. HV, high velocity; LV, low velocity.

Numerous studies report dynamic interactions between θ oscillations and place-cell activity in the hippocampus of freely moving rodents [18,58–61], but this relationship has not been directly explored in primates. To address this issue, we next characterized the θ oscillation and its relationship to active place cells during marmoset spatial navigation. Oscillations were detected by identifying periods of local field potential (LFP) activity whose spectral content significantly deviated from the 1/f background [62]. A significant θ oscillation was identified during track recording in the LFP underlying 93% (269/289) of recorded place cells. In contrast to the continuous θ oscillation commonly observed during analogous experiments in the rodent, θ oscillations in freely moving marmosets occurred in bouts of activity with similar spectral and temporal parameters to those observed in humans and macaques [11,25]. Given the velocity-dependent modulation of θ that has previously been observed [63] and the presence of a temporally discontinuous oscillation, analyses were conducted to assess the potential effect of behavior on both spectral and temporal characteristics of the bouts. Specifically—in addition to θ power, frequency, and bandwidth—the dependence of θ bout duration and frequency of occurrence on behavior was analyzed to determine whether a temporal information channel relating to behavior existed within the observed θ bouts. In agreement with prior observations, θ bouts observed both during locomotion and at rest were highly band-limited and temporally discrete (Fig 7A). The bouts were consistently present in the 5 to 10 Hz range (mean lower and upper bounds) with a mean peak of 6.5 Hz (Fig 7B). Also noted was that the LFP power spectrum was quite consistent when comparing θ oscillation parameters across all recordings (Fig 7C), with no significant differences in the distribution of peak θ frequencies and θ band limits for comparisons across animals and across hemispheres (*p >* 0.05, ANOVA). Interestingly, neither bout duration (Fig 7D) nor bout power (Fig 7E) was significantly modulated by HV: >20 cm/s) versus low-velocity (LV: <20 cm/s, ≥0 cm/s) locomotion (*p* > 0.05, KS test) when bouts were pooled across all recording sessions. It is noted that the detected θ bouts were generally quite short compared with those observed in other nonhuman primates [11, 25], with the median HV bout duration being 0.4 s (HV μ = 0.41 s, HV σ = 0.074 s), and the median LV bout duration being 0.4013 s (LV μ = 0.41 s, LV σ = 0.073 s). A very small fraction of bouts was longer than 1 s in duration, with only 0.033% of bouts (14/41895) for the HV condition and only 0.014% of bouts (15/105537) for the LV condition. Given the peak frequency around 6 Hz and the mean bout duration of approximately 0.4 s, most bouts lasted 2 to 3 θ cycles. Properly estimating the phase for such short and potentially noisy LFP signals is challenging, particularly in a freely moving preparation in which rapid jumps in phase due to movement artifact could artificially create or destroy θ phase dynamics. However, the frequency sliding approach employed by the Multiple Oscillation Detection Algorithm (MODAL) algorithm is specifically implemented to handle such abrupt noise spikes [64] and, therefore, is well suited to estimate θ power and phase in the context of this experimental preparation. Nevertheless, in order to assess the capability of MODAL to extract oscillatory bouts of such short temporal extent, MODAL was also applied to synthetic LFP data with simulated bouts inserted (see Materials and methods; S10 Fig). For the bout frequency and duration ranges in question, the sensitivity of the algorithm was found to be 100% consistently. However, the specificity of 80.1% ± 4.1% (mean ± SD) indicates that some contamination of bouts with nonbout activity could be occurring for the θ bouts of interest, and caution should be exercised because of the possibility of false positives.

**Fig 8:**
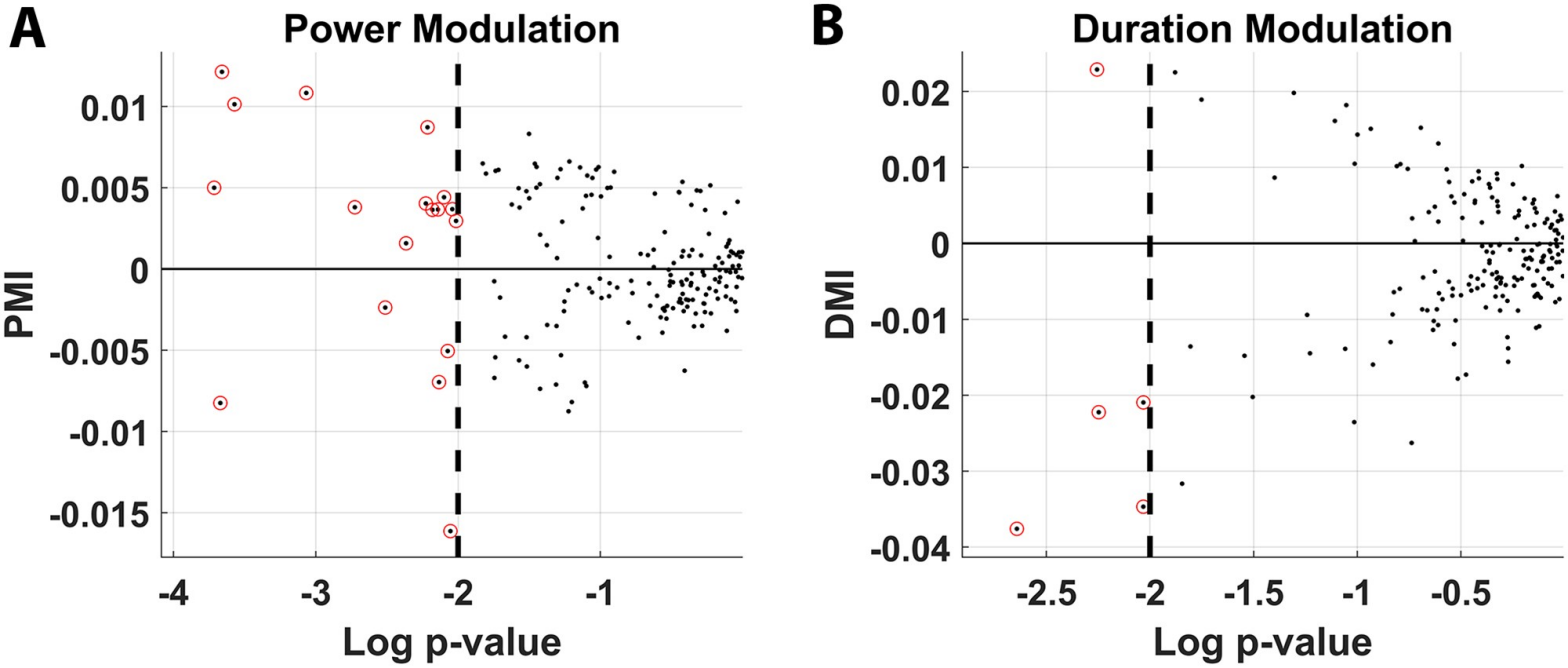
Modulation of θ oscillations during HV and LV locomotion. Mean power (A) and mean duration (B) modulation indices for each channel are plotted as a function of the *p*-value of the difference between HV and LV bout distributions by KS-Test. Positive modulation indices indicate higher mean power and longer mean duration during HV bouts in (A) and (B), respectively. Each black dot represents a test session. Channels whose power or duration modulation index was statistically significant by KS-Test at the 0.01 level are circled in red. A significant number of channels exhibit power modulation (18/185, *p <* 0.01 BT) and duration modulation (5/185, *p* = 0.039 BT). However, the relatively low magnitude of observed modulation indices indicates that the HV and LV mean powers and durations are predominantly within a scalar factor of only 0.95 of each other. The underlying data can be found at https://doi.org/10.5061/dryad.kk63d49. BT, binomial test; HV, high velocity; KS, Kolmogorov-Smirnov Test; LV, low velocity; PMI, Power modulation index.

To determine whether data pooling was masking locomotion-based θ modulation at isolated recording depths, bout power and duration were further analyzed for each recording session and then again on each channel. Recording session analysis revealed that, even though several recording days did exhibit significant locomotion-modulated bout power (22/37, S11 Fig) and duration (5/37, S12 Fig), none of these findings remained significant when *p*-value thresholds were corrected for multiple comparisons. The magnitude of the increases in mean power and duration were also small compared with broad-scale network change observed in rodents at the LFP level and were in concordance with prior findings observed in other primates [65]. Analysis of θ bouts on a channel-by-channel basis was also conducted. In this context, binomial tests (BTs) were conducted using a probability parameter equal to the *p*-value threshold for each analysis to determine a reasonable lower-bound probability for a given number of channels significantly exhibiting a characteristic by chance. Channel-wise analysis revealed that, even though a significant number of channels exhibited θ bout power modulation (18/185, *p <* 0.01 BT) and duration modulation (5/185, *p* = 0.039 BT), once again, the amplitude of this effect was limited in all cases (Fig 8A and 8B). In fact, the magnitude of observed modulation indices indicates that the HV and LV mean powers and durations are predominantly within a scalar factor of only 0.95. The strongest of these effects was a 2.1% increase in bout duration for HV compared with LV. Furthermore, the significant changes in bout power and duration were not consistently positive or negative, with a majority of channels exhibiting significant increases in bout power and decreases in bout duration when comparing HV and LV. Furthermore, there was not a consistent modulation between HV and LV, with θ bout power being suppressed (Power Modulation Index [PMI] < 0) during HV movement in 14/18 significant channels and θ bout duration shortening during HV movement in 4/5 significant channels. The lack of consistency in these findings suggests a departure in phenomenological dynamics of the θ oscillation when comparing freely locomoting rodents and primates in regions of the hippocampus where place-cell activity is observed. An important caveat to this conclusion, however, is that we are assuming the relationship between continuous θ oscillations and locomotion in rodents are applicable to the intermittent bouts observed in marmosets. It is entirely possible that the nature of hippocampus θ bouts in primates may preclude an interaction with this particular behavior, though other exploratory behaviors in primates can modulate θ [11].

**Fig 9:**
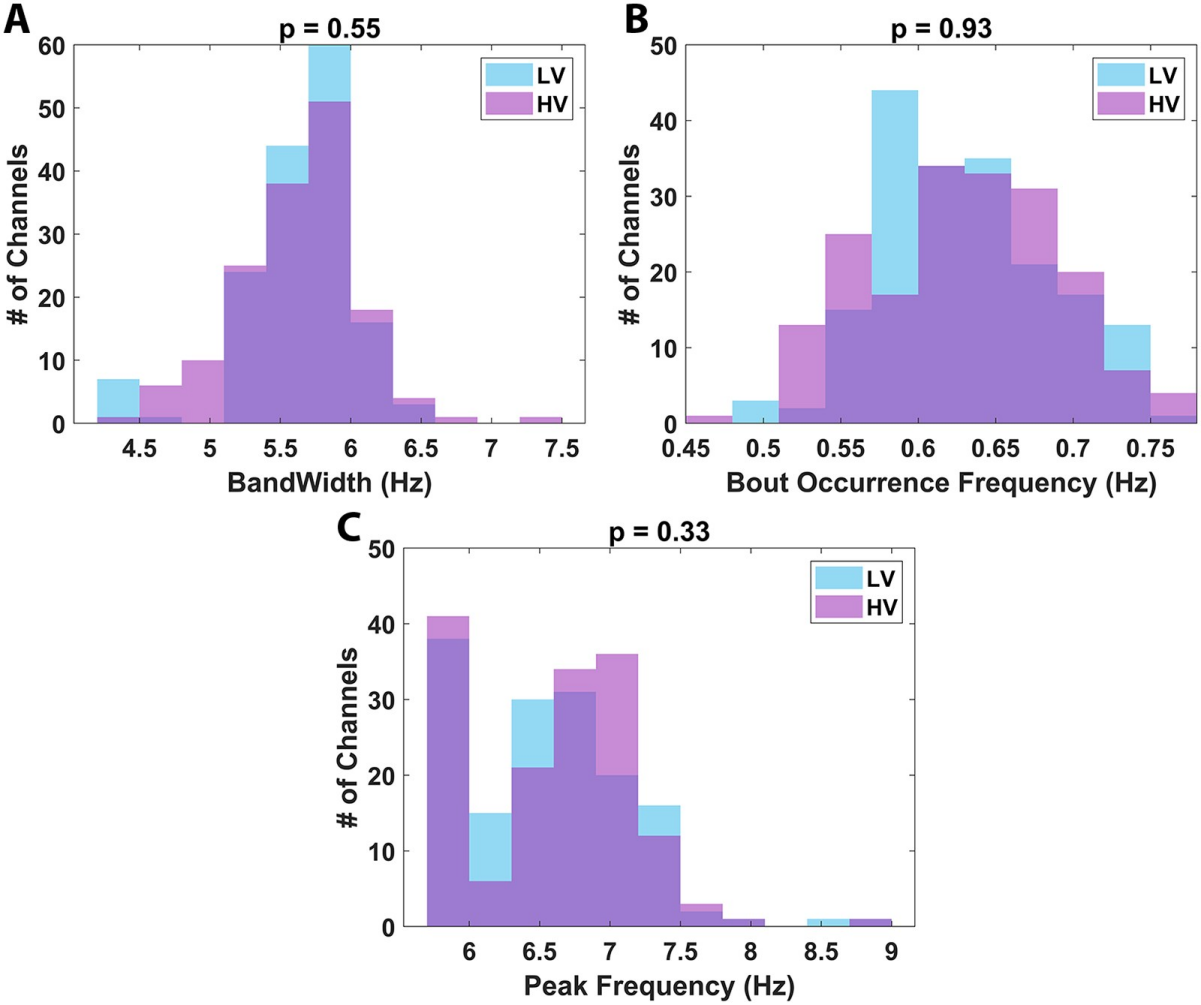
Locomotion-related modulation of the θ oscillation. (A) Comparison of θ-oscillation bandwidth between HV (purple shaded) and LV (blue shaded) locomotion pooled across all recording sessions. (B) Comparison of θ-bout frequency of occurrence between periods of HV and LV locomotion along the L track pooled across all recording sessions. (C) Comparison of θ-oscillation peak frequency between HV and LV locomotion pooled across all recording sessions. All reported *p*-values shown above each plot were computed using a paired signed rank test between the HV and LV oscillation parameter for each recording session. The underlying data can be found at https://doi.org/10.5061/dryad.kk63d49. HV, high velocity; LV, low velocity.

Further analysis revealed a lack of significant locomotion-related modulation on the bandwidth, occurrence frequency, and peak frequency of θ bouts (Fig 9). Out of concern that intermediate velocities, i.e., 5 to 20 cm/s, may have been masking a significant difference between the HV and LV groups, the bandwidth, occurrence frequency, and peak frequency comparisons were recomputed after excluding those bouts whose mean velocity occurred within the range of 5 to 20 cm/s. Even with these restrictions on the LV distribution, no significant difference was observed between the LV and HV groups (*p >* 0.05 in all cases). These results support the notion that, despite the occurrence of θ oscillations during nearly all neural recordings with place cells in marmoset hippocampus, locomotor behavior did not modulate θ oscillations in this freely moving primate across multiple metrics. This overall pattern demonstrates a significant divergence from analogous analyses in rodents.

**Fig 10:**
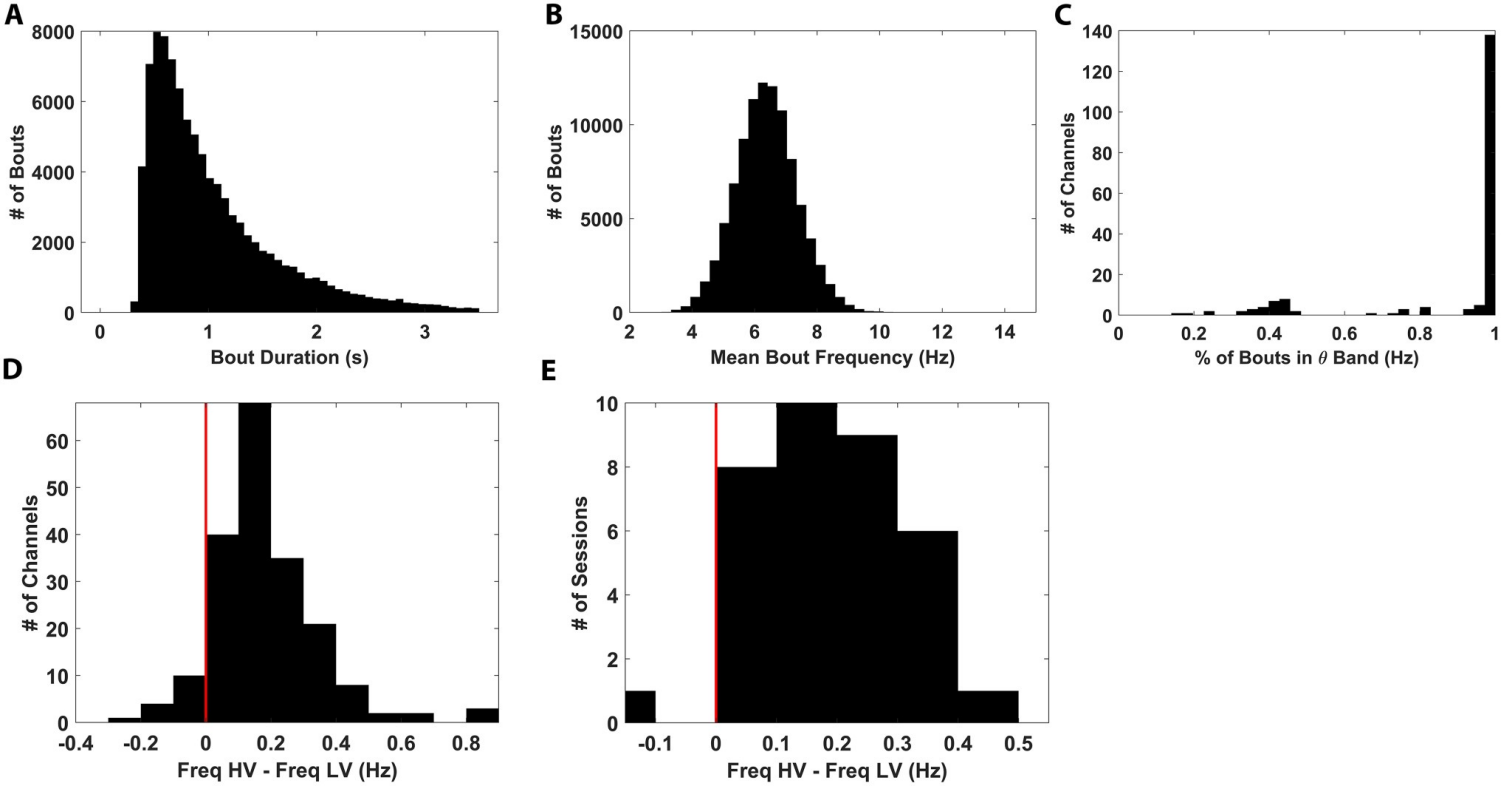
Analysis of nonrhythmic LFP bouts. Note that “bouts” in this case refers to the nonoscillatory cycle defined bouts defined agnostically of any oscillations. (A) Distribution of bout durations pooled across all channels and recordings from all subjects. Bout duration is reported in seconds. (B) Distribution of mean bout frequency across all channels and recordings from all subjects. Mean bout frequency is reported as the average frequency of all cycles included in a given bout, reported in hertz. (C) Distribution of percentage of bouts whose mean frequency fell within the MODAL-defined θ band is shown for all channels. The fraction for a given channel was computed as number of bouts within the MODAL θ-band limits for that channel divided by the total number of bouts detected on that channel. (D) The distribution of frequency differences between bouts occurring during HV and LV movement was computed for all channels. (E) Plots of the same analysis as in panel D but averaged across channels and reported for all sessions. Mean bout frequency was computed as in panel B. (D, E) Red vertical line indicates 0 Hz difference in mean frequency between HV bouts and LV bouts, and positive values indicate Freq HV > Freq LV. The underlying data can be found at https://doi.org/10.5061/dryad.kk63d49. HV, high velocity; LFP, local field potential; LV, low velocity; MODAL, Multiple Oscillation Detection Algorithm.

Given the absence of locomotion-dependent θ modulation, we sought alternative mechanisms by which the LFP might encode information about the behavioral state of the marmosets on the track. Recent findings in the hippocampus of fruit bats suggest that nonrhythmic LFP dynamics are an available information channels whose content reflects behaviorally relevant information during locomotion [66]. To test whether a similar pattern would emerge in marmoset hippocampus, we applied this same analysis to the current data. Specifically, cycles of nonoscillatory LFP fluctuations were detected by low-pass filtering (1–10 Hz) and identifying the troughs in this broadband signal, which defined the end points of consecutive cycles. Subsequent instantaneous power estimation via a Hilbert transform then allowed for exclusion of cycles falling below the 25th percentile, after which bouts were defined by linking together consecutive cycles (see the Nonoscillatory LFP analysis section in Materials and methods). This new bout definition yielded significantly longer bouts of activity (μ = 1.12 s, σ = 0.865 s) compared with the θ bouts detected by MODAL (μ = 0.41 s,σ = 0.079 s; Fig 10A); although, interestingly, the mean frequency content of these nonrhythmic bouts was quite similar to that observed when specifically identifying θ bouts in this frequency range (μ = 6.39 Hz,σ = 0.998 Hz; Fig 10B). To assess the congruence in bout frequency content between these nonoscillatory bouts and the θ bouts detected by MODAL, the fraction of nonoscillatory bouts whose mean frequency fell within the bounds of the defined θ band for each channel was computed across all channels, the results of which are shown in Fig 10C. Clearly, the vast majority of the bouts detected by this approach fell within the confines of the computed θ bands, with the vast majority of channels exhibiting over 95% congruence rate between the θ band and these bouts. Subsequent analysis of the behaviorally gated modulation of the frequency content of these bouts revealed that a small increase in frequency of on average 0.31 Hz was observed when comparing periods of HV and LV locomotion as previously defined, similar to previous observations [66]. This effect was present both at the level of individual channels (Fig 10D) and at when averaging over entire recordings (Fig 10E). Curiously, these findings conflict with the prior analysis conducted on HV and LV θ bouts that demonstrated a lack of significant frequency modulation, with the critical difference being that the bouts computed here are done so independently of narrow frequency bands, such as that defined by a θ oscillation, and are intentionally increased in temporal extent to span multiple cycles. These findings suggest that there may, in fact, be a behaviorally modulated information channel present within this broad low-frequency band, as evidenced by the θ coverage of these bouts, but that methods that identify oscillation bouts on a cycle-by-cycle basis such as that employed by Talakoub and colleagues [65] are more sensitive to the presence of this behavioral modulation.

### Spike-field interactions were limited in marmosets

**Fig 11:**
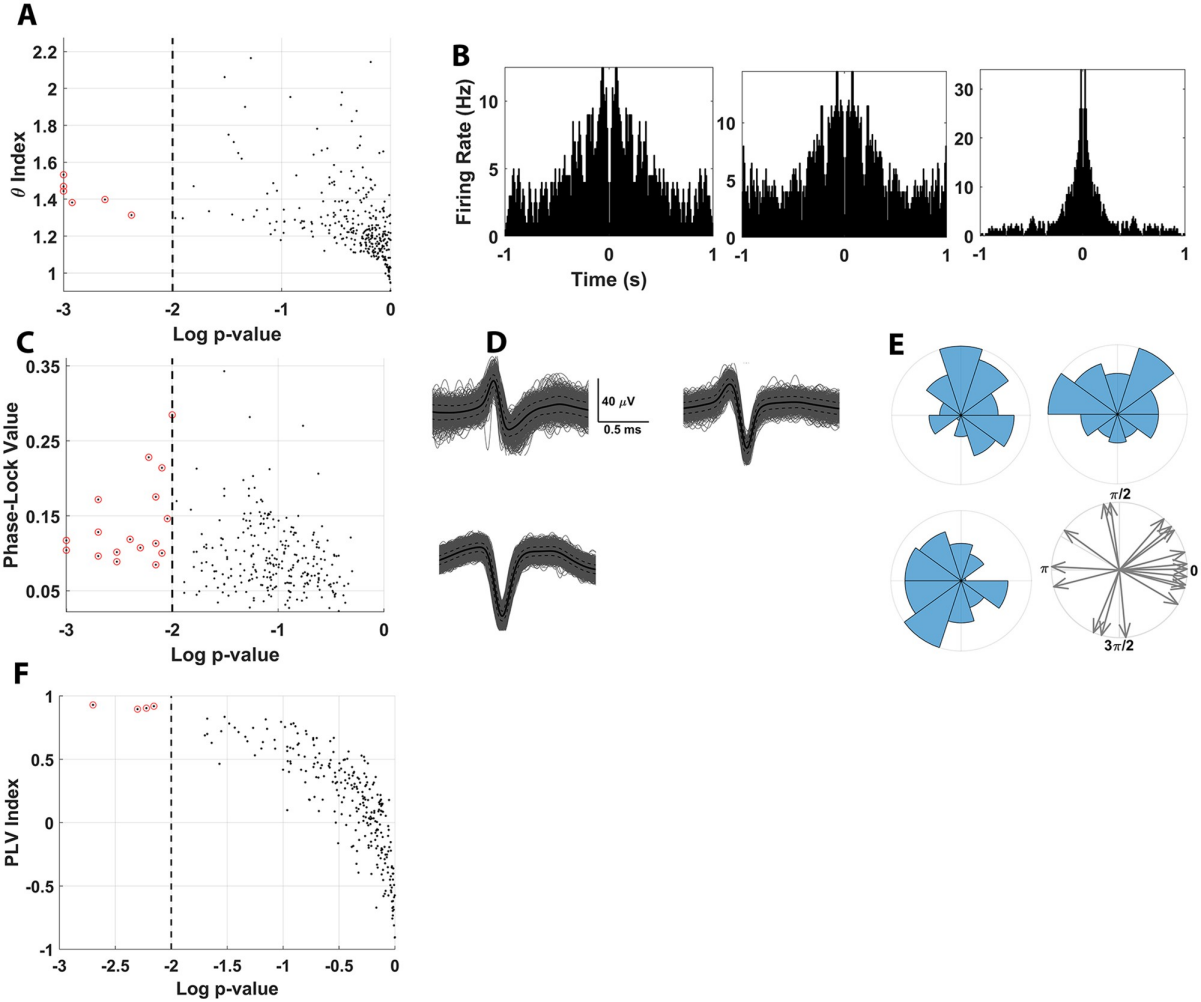
Spike-θ interactions in marmoset hippocampus. (A) θ-index modulation are plotted as a function of the *p*-value on all recorded place cells revealed 6/289 significantly modulated place cells at *p* < 0.01 significance (*p* = 0.072, binomial test). Black dots indicate individual neurons, whereas red circles denote those units exhibiting statistical significance. (B). Shown are ACFs for the 3 units with the lowest *p*-values in panel A. A visible θ oscillation covarying with the HV ACFs computed is evident for each of these neurons. (C) PLV for each place cell, an indicator of θ-phase coherence, is plotted as a function of the *p*-value. Higher PLV indicates stronger coupling to a specific phase within the oscillation. A total of 18/269 place cells (with θ oscillations) exhibited HV θ-phase locking at *p* < 0.01 significance (*p* < 10^−10^, binomial test). Black dots indicate individual neurons, whereas red circles denote those units exhibiting statistical significance. (D) Cluster waveforms for the 3 most significant units from panel C are shown. (E) θ-phase firing distributions associated with the waveforms plotted in panel D. Note the position of each spike cluster in panel D corresponds to the position of the θ-phase distribution shown in panel E. θ-phase distributions were computed with 36° bins and were normalized to the bin with the maximum number of spikes. Panel E, lower right plots the preferred phase of all units with significant phase locking. (F) The LFP-based PLV index is plotted as a function of the *p*-value for each place cell. A total of 4/269 place cells were significantly modulated (*p* = 0.28, binomial test). Black dots indicate individual neurons, whereas red circles denote those units exhibiting statistical significance. Positive PLV indices indicate stronger phase coherence during HV locomotion and vice versa for negative PLV indices. For panels A, C, and F, significance was determined by empirical *p*-value computed on shuffled null-distributions. All *p*-values were computed one-sidedly, hence the lack of neurons exhibiting significant negative PLV-index in panel F. The underlying data can be found at https://doi.org/10.5061/dryad.kk63d49. ACF, autocorrelation function; LFP, local field potential; HV, high velocity; PLV, phase locking value.

To address questions of phase coding in the marmoset hippocampus, the following series of analyses were performed. We first examined the intrinsic oscillatory modulation of place-cell firing in the θ frequency band independently of the recorded underlying LFP. Though LFP data were leveraged in subsequent analyses, these initial analyses were conducted to probe for the presence of behaviorally dependent changes in the spike-train frequency content of place cells independently of any underlying oscillatory bouts. Our metric of choice—the θ index—quantifies the degree to which θ rhythmicity in the autocorrelation of individual spike trains is modulated by animal locomotion. We computed this index for all recorded place cells and found 6/269 (2.2%) were significantly modulated at *p* < 0.01 significance. The number of neurons exhibiting this characteristic, however, was not greater than would be expected by chance using a binomial test (Fig 11A). This finding is consistent with observations in free-flying Egyptian fruit bats [3] and differs from the θ dynamics prominently observed in locomoting rodents [18]. It is noted that a visible θ envelope did co-occur with the HV autocorrelation functions for the top 3 exemplar units (Fig 11B). Nevertheless, the conclusions that can be drawn about the role of θ modulation in the locomoting primate are limited by the fact that the number of neurons exhibiting θ modulation is not significantly different from chance in this data set.

We next calculated a phase locking value (PLV) as an indicator of spike-field synchrony to directly investigate the interaction between the spike timing of single units during HV locomotion and θ oscillations. The PLV acted as an indicator of the consistency with which place cells discharged at a given phase in the oscillation, with a perfectly synchronous spike-phase interaction (i.e., always spiking at the same phase) yielding a PLV of 1 and a perfectly asynchronous interaction (i.e., spiking with a uniform distribution with respect to the phase) yielding a PLV of 0. Thus, the PLV was used to measure static, consistent interactions between place-cell firing and θ phase for each neuron individually. Furthermore, the limitation of this analysis to HV locomotion was intentional given that the phenomena of interest (i.e., θ synchronization and phase precession) are normally present during locomotion in rodents. Furthermore, the lack of significant locomotion-related modulation of the θ-bout characteristics did not preclude the possibility of transient phase coding at the level of the place cells occurring in a behaviorally sensitive manner. Thus, the HV-limited analysis revealed 18/269 place cells (6.7%) exhibiting θ PLVs at *p* < 0.01 significance. This proportion of the population, although notably low, was greater than would be expected by chance (*p <* 0.01 BT; Fig 11C). The 6 units with a significant θ index also had significant PLVs; a pattern that is shown in the 3 most statistically significant units by plotting both their respective cluster waveforms (Fig 11D) and θ-phase firing distributions (Fig 11E). Because the θ index and PLV are not directly analogous metrics of θ modulation, we also computed a PLV index as a means of assessing the degree to which the observed phase locking was driven by locomotion. Using an analysis that directly paralleled the θ index, only 4 of 269 (1.49%; Fig 11F) place cells exhibited a significant locomotion-driven increase in PLV. Once again, the number of neurons observed with θ modulation as defined with this metric is not significantly greater than chance. Despite the same neurons that exhibited significant modulation with the θ index were also significant with the PLV-index, the neuron count exhibiting this dynamic is simply not high enough to support the presence of θ modulation in these neurons. Although these measures of θ-phase coding in marmoset place cells demonstrate somewhat different outcomes, each metric explicates a different facet of spike-field interactions. Consistent across all of these analyses, however, is how few marmoset place cells exhibited significant spike-field interactions in this population.

**Fig 12:**
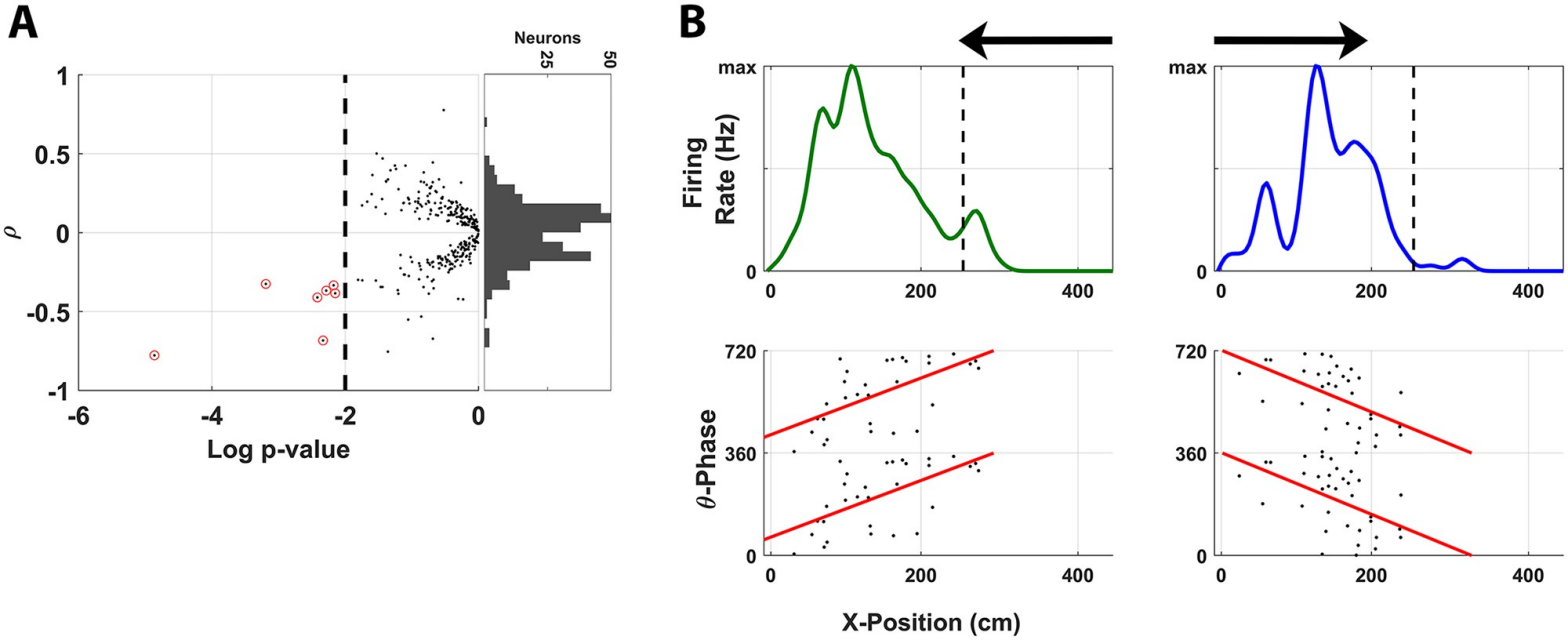
Place-cell phase precession in marmoset hippocampus. (A) The phase-location correlation *ρ* is plotted against the log *p*-value in panel A for all place cells indicated by black dots, with neurons clearing the *p*-value threshold circled in red. Neurons exhibiting significant θ phase precession (*p* < 0.01, linear-circular correlation) during concurrent HV locomotion and θ bouts were also identified (7/269, *p* = 0.019 BT). Negative correlation indicates a place cell fired earlier in the θ phase as the marmoset traversed the place field. Right shows the distribution of correlations across the neural population. (B) The top plots the mean firing rate of 2 neurons as the marmoset locomoted across the L track. The top left plots an exemplar of an LSPF, whereas the top right plots an exemplar of a RSPF. The place field for each cell is also shown with an arrow indicating the direction of travel for which the cell was sensitive. The bottom plots spikes discharged during a θ bout as the place field is traversed using the instantaneous θ phase and marmoset position for the corresponding units above. The correlation in spike timing between θ phase and position along the L track is indicated by the least-square fit line (red). The underlying data can be found at https://doi.org/10.5061/dryad.kk63d49. HV, high velocity; LSPF, left-selective place field; RSPF, right-selective place field.

Given these findings for static spike-θ phase interactions, we hypothesized that dynamic spike-θ interaction, namely, θ phase precession during HV locomotion, would be equally sparsely present within our population of place cells. We probed for the occurrence of phase precession by computing linear-circular correlations between θ phase and position for spikes discharged during a θ bout and during HV locomotion in the direction of travel encoded by a given neuron. This analysis revealed evidence of phase precession in a handful of neurons from this population (*n* = 7/269; 2.6%; *p* = 0.028 BT). Every incidence of significant phase precession in this population was a “negative” correlation (Fig 12A, left). In other words, the spiking phase began late in the oscillation and progressively shifted earlier in phase as the marmoset traversed the place field. No significant positive correlations were observed in this population. It is further noted, however, that the distribution of correlations (Fig 12A, Right) was not significantly skewed in either the positive or negative direction, suggesting that there is not a consistent population-level implementation of phase precession, as has been observed in rodent place cell ensembles during locomotion [67]. This analysis was repeated with a subpopulation of place cells that fell within the bottom 50th percentile for place field width and the top 50th percentile for cluster isolation quality as determined by the L ratio and ID computed previously. Of the 112 neurons included in this new subpopulation, all 7 exhibiting significant phase precession were present. Although the fraction of neurons from this subpopulation exhibiting significant phase precession was more statistically robust (*n* = 7/112; 6.3%; *p* = 1.5 × 10^−4^ BT), no significant skew in the subpopulation phase correlation distribution was present in this case. Although phase precession was only identified in a small number of units compared to many analogous rodent studies, the consistency of precession direction for significant units and the clear precession effect present in representative exemplar neurons from this population (Fig 12B) suggest that this feature is present to some degree in marmoset hippocampus during locomotion.

The presence of θ sequences was investigated as another potential indicator of spike-phase interaction given that it has been identified in rodents as a direct consequence of phase precession [21,68]. Though not purely a trivial consequence of phase precession [69], a relationship between the sequential firing of place cells at the time scale of the θ oscillation and at the time scale of behavior would provide strong support for the presence of θ-based phase coding. To investigate this, the peak distance between concurrently recorded place fields was compared to the peak cross-correlation latency at the 2 different time scales—the behavioral and θ scales. Though a highly significant (*p <* 10^−10^) correlation between place field distance and cross-correlogram (CCG) latency was identified at the θ time scale (S13A Fig) with *ρ* = 0.30, the fraction of variance explained (R^2^ = 0.07) was very low compared to the behavioral time scale correlation (S13B Fig) with *ρ* = 0.68 and R^2^ = 0.46. Given the possibility of θ sequences only arising in the presence of a significant θ oscillation, the analysis was reconducted only with spikes occurring during θ bouts, which caused the place field (PF) peak distance/CCG peak latency correlation to plummet at both time scales (S13C and S13D Fig). Like the previous phase precession analysis, here, we observed some limited evidence supporting the presence of phase coding dynamics at the θ time scale in marmosets, but the prevalence is much lower when compared to analogous findings in rodents [70,71].

**Fig 13:**
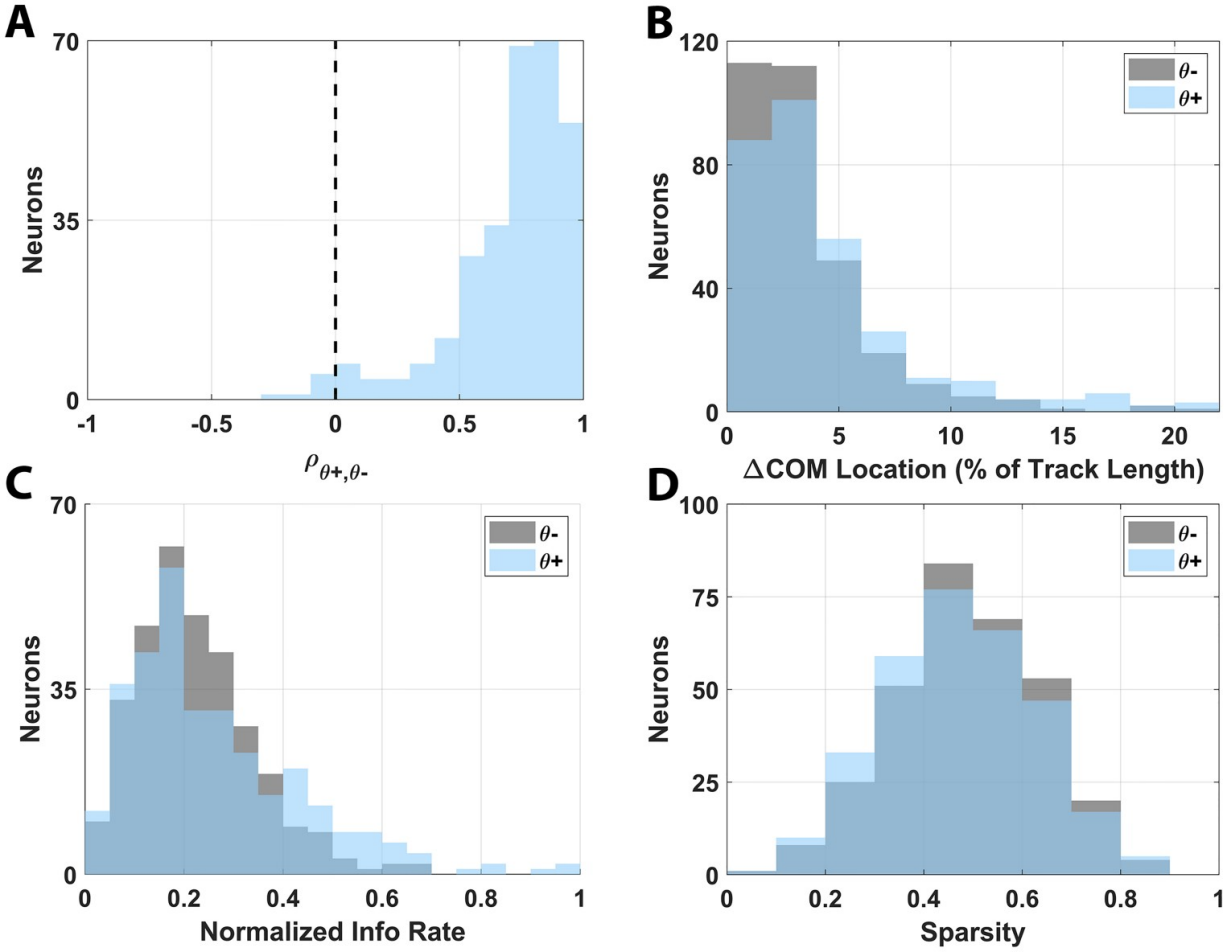
Analysis of θ-driven place-field representations. (A) Distribution of place-field correlations encompassing the θ bout (θ+) and nonbout (θ−) periods during HV track traversals is shown. (B). Distributions of place-field location encoding through the COM separated by θ+ (blue shading) and θ− (gray shading) periods are shown. (C) The distributions of information transfer rates in the θ+ and θ− periods are provided. (D) The distributions of the sparsity of the place fields along the L track for both θ+ and θ− periods are provided. The underlying data can be found at https://doi.org/10.5061/dryad.kk63d49. COM, center of mass; HV, high velocity.

The presence of θ bouts provided a unique opportunity to study the potential effects of intermittent θ on the gating of spatial information encoded by place cells. To explore this dynamic, place fields for all recorded neurons were recomputed separately using spikes occurring within θ bout and nonbout periods during HV track traversals (θ+ and θ−, respectively). Following this separation, we sought to characterize the effect of θ bout occurrence on the rate coding of the place-cell population during locomotion. The computed place fields across the neuronal population were highly correlated between θ+ and θ− periods (*μ* = 0.72 ± 0.23, Fig 13A), indicating that gross place-field characteristics were unchanged by the presence of θ. This finding was supported by a direct analysis on the change of encoding position as quantified by the center of mass of each place field, which showed that place-field location was not significantly altered for either the θ+ or θ− condition. In both cases, the majority of place cells (89.5% and 95.9%, respectively) encoded a location within 10% track length of the combined θ+/θ− place field and the distribution of encoding location changes with respect to the combined field was insignificantly different between the θ+ and θ− condition (Fig 13B), suggesting no consistent changes in location encoding as a consequence of the presence of θ bouts. Furthermore, there was not a significant difference in either the amount of spatial information encoded by the place fields in the θ+ and θ− conditions (*p* > 0.05 KS-Test, Fig 13C) or in the sparsity of the place fields along the track (*p* > 0.05 KS-Test, Fig 13D). Together, these findings suggest that the presence of θ bouts during HV locomotion did not grossly affect the spatial representations of place cells in the primate hippocampus and did not gate change in rate-coded information transfer to downstream neuronal populations.

## Discussion

The question of how the primate hippocampus represents space has been considered since the seminal discovery of place cells in rats [1], though efforts to examine neural activity in the hippocampus during free navigation in primates have been notably limited [7,15,16]. Here, we sought to bridge that gap by recording the activity of single neurons in the hippocampus of freely moving marmosets as they naturally explored a physical environment. By testing subjects in linear environments commonly used in studies of rodents, we aimed to more directly compare results between these mammalian taxa as well as identify similarities and differences that might occur. Using this approach, we report the evidence of neurons in the marmoset hippocampus that encode self-position—place cells—during freely moving exploration. These place cells showed notably similar properties to other mammals [2,3], suggesting potential parallels across taxa for this neural population. In stark contrast to comparable studies in rodents, however, θ oscillations in the marmoset hippocampus were weakly correlated with either place-cell activity or locomotion. Overall, these findings paint a particularly intriguing comparative picture of the respective roles that these distinct neural signals may play in representing space across vertebrates.

A key implication of this study pertains to the role of local field potentials in primate spatial representations. Specifically, we recorded θ oscillations in parallel with nearly all marmoset place cells, demonstrating that this signal is prevalent in the primate hippocampus. Like previous reports in head-restrained macaque monkeys [11], this neural signal in marmosets occurred in sporadic bouts rather than continuously. The similarity in the pattern of LFP activity between these 2 studies—one in freely moving animals and one in head-restrained subjects—is suggestive that some degree of bout-based θ phase coding may be characteristic of the primate hippocampus. Although θ oscillations factor prominently in models of hippocampal spatial encoding in rodents [18,19,22], we found that θ oscillations exhibited limited interactions with place-cell activity in the hippocampus of a freely moving primate, a result consistent with recent findings in free-flying Egyptian fruit bats [3,23,66] and the big brown bat [72]. In other words, self position can be encoded in hippocampal neurons without the strong influence of this oscillation for at least 3 nonrodent mammalian species. Although the anatomy of the hippocampal formation has remained relatively conserved across mammals [73], primates and rodents differ in numerous characteristics, ranging from the details of their respective exploratory behavior, dominant sensory modalities, the relative expansion of neocortex, and the evolution of the lateral prefrontal cortex in primates [28,74–79]. It should be noted that our results do support a previously uncharacterized level of neurophysiological conservation in the structure of the primate hippocampus. Specifically, the progressively increasing spatial scale of place fields observed in rodent hippocampus [49] is indeed a feature of the marmoset hippocampus when recording along the homologous long axis. However, direct parallels between axis locations between rodent and primate hippocampus are difficult to draw using the place cells we recorded because of systematic differences in size and information content compared to those found in rodents [47,49,52]. These differences might have arisen for a number of different reasons. Many of our place cells were recorded in the more anterior segments of hippocampus, which is expected to carry a more spatially diffuse representation of space at the single-neuron level. Thus, more targeted recordings specifically being conducted at the posterior end of primate hippocampus might be necessary to identify the place-cell population that matches the rodent dorsal hippocampus. The concomitant representation of visual space might also be implicated in the decrease in spatial information encoded by primate place cells, but experiments involving concurrent eye tracking and free navigation would need to be conducted to determine whether mixed selectivity such as this even exists. It is also entirely possible, albeit quite speculative, that the involution of the dorsal hippocampus known to occur in both primates and rodents during embryonic development [80,81] is asymmetric in such a way that the homolog to rodent dorsal hippocampus simply is not present in primates. The available physiological evidence suggests caution in assuming that patterns of hippocampal activity in rodents are directly predictive of analogous processes in primate brains but does indicate that many new lines of inquiry are required to better characterize the separating boundary between mammalian taxa.

The emerging comparative data on hippocampal θ oscillations provide clues about its functional role and suggest a distinction between 2 facets of spatial navigation: exploration and self position. Exploration refers to actions related to constructing a representation of the environment, whereas self position refers to one’s location in the environment. In nocturnal burrowing animals, such as rodents, these 2 facets of navigation are closely coupled because there are fewer distal sensory cues with which to construct a map of space prior to moving through it. Certainly, the relationship between these neural signals is not static in rodents, because it is affected by characteristics of behavior that may reflect the distinction between exploration and position [82]. In species for which these 2 navigational components are less temporally integrated, the relationship between θ oscillations and place cell activity is likewise more weakly coupled. Primates, for example, tend to explore the world visually prior to actively locomoting through it. Here, we observed notably limited spike-θ coherence for self position. Visual exploration of space through saccadic eye movements can reset the phase of θ rhythms in the rhesus monkey hippocampus, suggesting a potential interaction between this neural signal and visual exploration [11]. Likewise, θ oscillations in big brown bats exhibited a bout-based structure similar to primates, but—unlike our simian cousins—this local field potential only emerged during explicit instances of echolocation as the animals explored the environment acoustically [72]. It is possible that, because primates are constantly exploring the world visually, the bouts of θ oscillations are more constant than in echolocating bats, for which periods of exploration may be more intermittent. If the computational role of θ is more closely coupled to exploration than self position, current models of its role in spatial navigation may not fully capture its function in the human and nonhuman primate hippocampus. To test this hypothesis, experiments that record neural activity in primate hippocampus as monkeys both visually explore an environment and change their position within that same space through active locomotion are needed.

The results presented here suggest a fascinating convergence of neural signals related to spatial encoding in the primate hippocampus whose interaction must be explicated to more fully understand the neural basis of spatial navigation in our order. Findings that neurons in primate hippocampus represent one’s self position in space—as shown here and previously [16]—complement experiments demonstrating that neurons in this same population can encode the spatial configuration of the environment visually [10,11,26,83]. Precisely how these 2 likely complementary systems for exploring and self position intersect mechanistically is virtually unknown. A second dimension of hippocampal function in this context relates to the role of θ oscillations. Although we observed little coupling between this neural signal and place cells in free navigation here, the timing of hippocampal θ oscillations are affected by saccadic eye movements [11], suggesting a further layer of dynamic interactions within this system that incorporates the myriad of related medial temporal lobe processes (e.g., decision making, planning, episodic memory formation [5,9,11,38,83–85]) and potential idiosyncratic mechanisms in the primate brain. Finally, these results paint an exciting comparative portrait by bridging several gaps in our understanding of the hippocampal mechanisms that support spatial navigation across mammals. Although our data indicate the presence of some core characteristics that are shared across multiple taxa—such as neurons that encode self position—the particular suite of properties evident in the marmoset hippocampus may be distinct to primates, though data from other species during free navigation are clearly needed to reconstruct a more comprehensive phylogenetic framework [86]. Moreover, the lack of clear anatomical specificity for which hippocampal subfields were recorded and the use of only linear track environments are clear caveats to this study that necessitate further study. Notwithstanding the limitations of the current study, the stage is set for an exciting new chapter of research aimed at further explicating these issues, one that includes marmosets as a powerful species in which to examine the role of the hippocampus in primate spatial navigation and other medial temporal lobe functions [37].

## Materials and methods

### Ethics statement

All experiments in this manuscript were approved by the University of California, San Diego, Institutional Animal Care and Use Committee (Protocol S019147) and in accordance with National Institutes of Health and the American Veterinary Medical Association guidelines.

### Subjects and surgical preparation

Two adult marmoset monkeys, one male (BL) and one female (TD), served as subjects in these experiments. All experiments were approved by the UCSD Institutional Animal Care and Use Committee. Subjects underwent an initial surgery to affix a head cap and post prior to beginning experiments. The procedure is necessary to restrain subjects’ heads during experiment preparation and to embed the drive housing and the electrode array for stable chronic electrophysiological recording. These surgeries were performed under sterile and anesthetized conditions. During these surgeries, a head post made of PEEK plastic was affixed to the skull using nylon anchor screws placed in approximately 1 mm holes and MRI compatible dental acrylic. Placement of anchor screws was planned such that the lateral temporal regions of the skull were left clear, leaving access for subsequent electrode array implantation. Subjects were given 1 month of recovery time prior to being subjected to further surgeries.

### MRI localization procedure

All marmosets underwent pre- and postoperative structural MRI scans in order to localize the body of the hippocampus within the medial temporal lobe during preoperative scanning, as well as to confirm correct implantation of the MBAs postoperatively. Three small saline barrels were attached with dental acrylic to the head cap to provide markers for stereotaxic alignment of the brain scan with the plane in which the implantation surgery took place. During MRI scans, animals were anaesthetized with 40 mg/kg of ketamine and head fixed in the sphinx position in a horizontal 7 T/20 cm MRI scanner (Bruker-Biospin Corp., Billerica) with Advance II hardware. T2-weighted RARE Imaging was performed with a Turbo Spin Echo (TSE) pulse sequence (TE/TR = 46.24/4000 ms; slice thickness = 700 μm, matrix 192 × 192, 6 averages, RARE factor 12, flip angle 180°) 200 μm isotropic resolution. This scan was developed to enhance contrast between the body of the hippocampus and the surrounding white matter to facilitate surgical targeting. As such, the hippocampal formation was identified in scans using locations of the temporal horn of the lateral ventricle, choroid fissure, and parahippocampal gyrus. Preoperative scans were used to optimize the trajectory for the microwire implant. The trajectory was measured from the lateral sulcus, lateral from the midline, aiming for the center of the hippocampal formation. The distance from the surface of the skull to the nearest edge of the hippocampus was also measured, and entry into the cortex was planned at an angle of declination from vertical. Postimplant, the location of the electrode bundle and guide tube were also confirmed with a FLASH scan (TE/TR = 25/50 ms; slice thickness = 937 μm, matrix 128 × 128, flip angle 10°). Quantification of implantation and recording sites was conducted by aligning a postoperative T2w MRI from each subject with a T2-Nissl co-registered atlas published by the RIKEN research institute [87]. The atlas and the T2 MRI from each subject were initially manually centered on the posterior edge of the anterior commissure. Each subject’s T2 was then warped to atlas space using standard volume co-registration and reslicing protocols available in SPM12. Regions of volume coverage at the initial implant site were identified by defining a spherical ROI with a 1-mm radius, the maximum possible splay of the electrode array; see below. Projection of these volumes onto the long axis of the atlas-defined volume of the hippocampus was used to determine the relative AP position of each implant.

### Brush array surgical preparation

Brush array implantation was conducted after subjects were given sufficient time to recover from head cap implantation. The multi-electrode array used for these experiments is a MBA produced by Microprobes for Life Science developed at the National Institutes of Health specifically for use in chronic single-unit recording preparations in nonhuman primates [88]. Our preparation involved housing the 64-channel MBA within a custom microdrive machined completely out of PEEK plastic to allow for postimplantation MRI. This microdrive was constructed such that the MBA and a static referencing electrode could be implanted and remain operational for long periods of time. The MBA was controlled by a drive screw with a 650-μm pitch allowing for fine-tuned positioning and mobilization of the MBA postimplantation. In addition to differential cortical referencing for LFP, an independent epidural ground wire was employed because this method of grounding was found to be optimal for line noise and artifact elimination. All surgical equipment and implanted devices were sterilized in a standard ethylene oxide sterilizer, allowing for the implantation to also be conducted under sterile conditions. During the implantation surgery, the microdrive housing the MBA initially housed a sharpened titanium stylus of the same gauge. After drilling the dental acrylic down to the surface of the skull, a trephine drill bit was used to create a 3-mm diameter craniotomy within which the epidural ground was placed and affixed to the surrounding dental acrylic. A reference electrode was implanted under the edge of the craniotomy and cemented in place with dental acrylic such that one end sat superficial to the periosteal dura and under the skull, and the other end remained accessible outside of the operating field for attachment during recording. At this stage, the stylus/microdrive assembly was mounted on a stereotaxic micromanipulator (Kopf Instruments) and adjusted to the proper rostro-caudal implantation position and angle of declination relative to the craniotomy using the preoperative MRI scan. A small durotomy was made in the center of the craniotomy through which the stylus/microdrive assembly was passed and advanced through the brain to a previously computed implantation depth. A bio-compatible silicone elastomer (Silastic, Dow Corning) was then used to plug the craniotomy underneath the microdrive, and a new dental acrylic was used to cement the drive in place on the skull. After the dental acrylic hardened, the stylus was removed from the microdrive, and the MBA was threaded down the guide tube of the drive and clamped in place. The connector block between the MBA and the amplifier head stage (Intan RHD 2164) to be used during neural recording was also cemented into dental acrylic and covered with a protective cap when not in use. Reference and ground wires were connected to this connector block using small wires protruding from the base of the block that were shorted to the reference and ground channels on the head stage. In some cases, motion artifact during preliminary recordings necessitated the connection of the ground wire directly to the ground on the amplifier.

### Experimental procedures—Track environments

Initial experiments were conducted on a linear track constructed of 80/20 Aluminum and acrylic wall and floor panels. This track was 244 cm long and 15 cm wide, creating a single principal direction of motion of travel for the marmosets introduced to the environment. The track was wide enough for marmosets to turn around comfortably but did not allow for significant movement in the off-axis direction. The interior walls and floor of the track were painted matte black with horizontal gray and white stripes on opposite ends of the track to allow the marmosets to orient themselves within the track, because the high walls (122 cm) prevented the marmosets from seeing the rest of the room. In addition to this linear track, an L track was constructed featuring 2 linear arms oriented perpendicularly and connected at a corner such that a marmoset traveling from one end of this track to the other needed to negotiate a 90° turn. The arms of the track were not symmetric in length; the long arm was 243 cm, and the short arm was 183 cm for a cumulative travel distance of 426 cm from end to end. Again, different sides of each arm of this L track were painted with different patterns to ensure each arm of the track was easy for the marmoset to visually identify. The acrylic panel walls were fitted to the track such that each could individually be removed to clean the interior of the track as well as to facilitate introduction and removal of marmosets to and from the track at the beginning and end of recording sessions.

### Experimental procedures—Recording sessions

Prior to conducting neural recording during locomotion, all subjects were given several days to acclimate to the track environment because we found that, upon first introduction to the track, the marmosets would remain in a single corner of the track and would not physically explore. This period of acclimation was completed prior to implantation with an MBA so that, following implantation and postoperative MRI, recording with exploratory behavior could begin after recovery. Individual recording sessions consisted of recording from a single 64-channel MBA using a custom head stage to ensure good recording quality during track exploration. The head stage consisted of an Intan RHD 2164 amplifier encapsulated by a custom-machined PEEK plastic casing that protected the amplifier while allowing it to be connected to the connector block (64 Channel Omnetics Micro-connector) in the dental acrylic of the current subject. Set screws at the base of the plastic cover fastened the head stage to the connector block to prevent detachment during recording. The SPI cable that connected the head stage to the Intan control board was protected by a metal coil glued to the top of the plastic connector and extended for 40 cm up the length of the cable to prevent damage incurred by the animals. In addition to the SPI cable, a cable connected to earth ground and separate wires powering the LEDs affixed to the head stage were routed through the protective coil and into the head stage.

On a recording day, subjects would be removed from their home cage and restrained in a custom-built marmoset chair used for head-fixed experimentation while the head-stage/tether assembly was properly attached and checked for unit activity. The subject was then removed from the chair and placed into the track environment, an overhead camera (GoPro) was turned on, and the neural recording initiated. In cases in which consecutive recordings were conducted to evaluate within-day stability of neuron waveforms and place fields, the neural recording and video recordings were terminated and the subject was removed from the track to be taken to an adjacent room for the 10-minute interrecording period. Following this period, the subject was retethered and re-introduced to the environment, after which video and neural recording recommenced.

At the end of each day of recording, the subject was head-fixed for implant cleaning and wound margin maintenance. During this time, the MBA was manually adjusted by advancing or retracting at least one-fourth turn (approximately 160 μm) in order to record from new neurons during the next track session. In order to account for potential tissue drag and repeated sampling of the same neurons across days, similarity analysis was conducted as described in the Data analysis—Neurons section. The results of this similarity analysis are shown in S14 Fig and demonstrate that very few of the place cells recorded on the same channel across consecutive recording sessions were consistent in their waveform shape, spiking statistics, and place selectivity on the track, suggesting that there was relatively little repeated sampling of neurons across days that might have been incurred through tissue drag when advancing the MBA.

### Video tracking

To track marmoset movements during experiments, 2 LEDs—red and green—were placed on the anterior and posterior edges of the marmoset’s head cap as it explored the track environments, allowing for position to be recorded using a high-resolution video camera positioned to capture the entirety of spatial environment (720 p resolution at 120 FPS). The camera’s intrinsic parameters were computed using the Scaramuzza calibration algorithm and were used to correct fisheye distortion in every video frame prior to marmoset position analysis. The marmoset’s position within the track was computed offline using a GPU-accelerated implementation of the mean shift algorithm. The initial ROI was a manually defined circle that encompassed the LEDs on the marmoset’s head cap. As this ROI tracked the position of the marmoset’s head, position was reported by the ROI center, generating an X and Y time series for location over the course of the recording session. The marginal RGB probability distributions required for the mean shift algorithm were computed by binning pixel intensities (range = 0–255) within each color channel of the initial ROI and convolving the resultant histograms with a Gaussian kernel (sigma = 8). Histogram back-projection was done on the GPU (GTX 1080) and achieved an acceleration factor of approximately 30× in comparison to conventional CPU implementations. The video recording and neural recording were synchronized using a blue LED placed on the side of the track. A square wave with a period of 1 s was used to drive both this LED and a digital input channel of the Intan amplifier, with analysis of both video position and neural activity only beginning from the onset of the first synchronizing pulse.

### Data processing

Isolation of single-unit activity from our extracellular recordings is achieved using a multistage fully automated computational pipeline that takes advantage of modern CPU multiprocessing capabilities in order to accelerate data processing as previously described in the work by Courellis and colleagues [89]. In brief, neurophysiological data were sampled at 30 kHz and stored for off-line analysis. The data from each channel were acausally filtered using a 500 Hz to 7 kHz Kaiser-windowed bandpass filter. Spikes were extracted by applying positive and negative thresholds, which were selected using a blanket $\pm 4{\hat{\sigma }}_{n}$ threshold (typically in the 30–50 μV range) that was computed independently for each channel. Spike detection was triggered on threshold crossing, and spikes were realigned to the peak/trough, followed by extraction of the 60 data points (± 1 ms) surrounding the new alignment point. The isolated spikes were subjected to presorting artifact rejection, which entailed removal of all waveforms whose maximum absolute voltage exceeded 1 mV and by removing all waveforms that occurred concurrently on more than 25% of the channels in the MBA (16 channels), because waveforms that fit these criteria were generally not neurophysiological in origin. From this point onward, all processing was conducted independently on a channel-by-channel basis. The spikes were then subjected to K-means clustering based on a combination of principal component (PC) features and multilevel wavelet coefficients. The feature space was defined by the top 3 PCs and the top 3 least Gaussian wavelet coefficients determined by the Shapiro–Wilke test for normality. To take advantage of parallel processing, spikes from a single channel were segmented into chunks of 20,000 that were clustered separately using K-means. Clustering outcomes were then merged across chunks using hierarchical clustering of all chunk cluster centroids. Manual curation of spike clusters following automated spike sorting was achieved by inspecting spike clusters in all possible pairwise 2D feature spaces defined by the same features on which the clustering took place. At this stage, cluster boundaries were manually shifted to exclude spikes from noise clusters that contaminated putative action potentials, under-clustering was resolved by manually defining separating hyperplanes in feature space, and over-clustering was corrected by manually merging clusters that clearly belonged together. Multi-units, or spikes drifting past one another, that could not be safely separated by manual intervention at this stage were discarded and not considered in subsequent analysis. Only spike clusters that were well isolated following both initial automated clustering and subsequent spike cluster curation by hand were retained for downstream analysis. It is noted that all 64 channels of the MBA were always subjected to spike sorting and subsequent manual inspection from every recording, yielding an average of 7.6 neurons per recording session. In addition to single-unit activity, local field potentials were also extracted from the MBA recordings.

The raw data was down-sampled from 30 to 3 kHz, which was well above the Nyquist limit for resolving LFP waveforms while still providing submillisecond temporal resolution for the LFP. Frequency bands of interest are isolated by applying an oscillation fitting algorithm called the MODAL [63] to identify deviations from the 1/f background (putative oscillations) and identify parameters of interest from these oscillations such as peak frequency and band limits. The algorithm was applied to the LFP data from each channel as follows: instantaneous spectral power was calculated between 3 and 25 Hz with a frequency resolution of 0.5 Hz using a 6-cycle Morlet wavelet convolution, followed by time averaging to compute the mean power spectrum for that channel. The 1/f background was estimated using a linear fit in log-log space, and segments of the power spectrum that rose above this line were defined as the oscillations present in the channel. Instantaneous frequency and phase for each band were then computed for the entire recording using the “frequency sliding” algorithm proposed by Cohen [64]. This algorithm is essentially just the calculation of the Hilbert transform of the bandpass filtered LFP signal followed by median filtering to remove rapid noise-driven phase discontinuities that tend to occur in electrophysiological data. To identify bouts, the 1/f background was refit into 10-s windows of the data, and only segments of the oscillation within the frequency bands defined by the full 1/f fit described above that exceeded the locally computed 1/f were flagged as bouts and retained for subsequent analysis. As such, the MODAL algorithm specifically allows for the detection of discontinuous oscillations, facilitating the analysis of oscillations occurring in bouts during locomotion. The start and end times of the bouts considered here were those returned through the MODAL analysis, and no post hoc joining of bouts was conducted such that the final temporal statistics of the bouts (i.e., duration and occurrence rate) reflect those statistics determined by the algorithm in a data-driven manner. The power spectrum computed by MODAL is shown in S15A–S15D Fig to demonstrate the presence of the θ-oscillation bump that was captured for every channel that exhibited significant oscillation.

To further assess the efficacy of MODAL in extracting short oscillatory bouts in a low SNR physiological setting, a number of simulations were conducted as follows. LFP data were simulated by creating 1/f distributed noise at 3 kHz with V_RMS_ determined by averaging V_RMS_ across all channels in all recordings. Bouts of frequencies that varied within the range of θ oscillations observed in our recordings were then injected with an occurrence rate that matched the observed occurrence rates of bouts in our recordings. The V_RMS_ of the simulated bouts was determined by estimating the oscillation SNR for each channel in which a significant θ oscillation was identified by computing the log-ratio of the maximum power within the oscillation band limit (the signal power) and the power of the 1/f background estimated at the oscillation frequency that exhibited maximum power. The number of bouts per simulated recording was controlled such that the simulated rate of bout occurrence was equal to the observed rate of bout occurrence during recording once the bouts were inserted into the simulated LFP; MODAL was applied to the signal using the same parameters employed to extract bouts from the recorded data as described in the previous section. The sensitivity and specificity of the algorithm were then computed by comparing the known bout occurrence times with those identified by MODAL. This procedure was repeated for bouts occurring at 6 Hz, 8 Hz, and 10 Hz, evaluating each case with bout durations varying from 300 ms to 1,000 ms in steps of 50 ms.

### Data analysis—Neurons

Place fields were computed by segmenting the track into 100 equally sized bins (approximately 4.3 cm/bin on the L track) and computing mean firing rate of each unit for every bin using only spikes that occurred during HV locomotion. These mean firing rate profiles were computed by generating occupancy maps separately for the left- and right-moving periods of the recording to account for the directional selectivity exhibited by the recorded neurons. Spikes discharged during recording were also segmented by direction of motion, binned identically by position discharged along the track, and normalized with respect to the occupancy map for that direction of motion. Thus, LSPFs were computed in units of the number of left-moving spikes per second of time moving left (Hz) and vice versa for RSPFs. Place fields were smoothed using a 5-point 1D Gaussian filter normalized for unit gain yielding 21.5 cm of effective Gaussian smoothing for the firing rate in each bin across the track.

In order to identify units whose firing rates were significantly modulated by the marmoset’s position along the track; the amount of information related to track position encoded by each unit was computed in each direction using firing rate as a function of track position through the Skaggs–McNaughton information index, a common evaluation procedure for place cells:

$$I=\sum_{x}r(x){log}_{2}(\frac{r(x)}{\overset{-}{r}})p(x),$$

where *r*(*x*) is the firing rate of the cell as a function of position along the track, $\overset{-}{r}$ is the mean firing rate of the cell throughout the recording, and *p*(*x*) is the probability of the marmoset occupying bin *x* along the track, which is computed from the behavioral data.

To determine whether the amount of information encoded by each unit in each direction was statistically significant, a shuffling procedure was used in which the spike times for each unit were randomly circularly shifted past the position information for the recording. The place field was recomputed using the new spikes that fell within the bounds of the HV locomotion periods in each direction. HV locomotion was defined as movement >20 cm/s, and LV locomotion was defined as all movement below this threshold. Notably, LV locomotion also included instances when the subjects were stationary. The information index was then recomputed for the new shuffled “place field” and an empirical *p*-value was computed by ranking the true information index against a surrogate distribution of *N* = 1,000 repetitions for each unit. To minimize false-positives in identifying neurons as place cells, the threshold for significance of *p* = 0.005 was selected, thus requiring that the place field of a unit in at least one direction of motion was above threshold in order to classify a unit as a place cell. Note that the Skaggs–McNaughton information index reports information in bits/spike, so the information rate of a neuron can easily be computed by multiplying the index by a neuron’s firing rate.

Place fields were characterized by the peak firing rate and associated location, which was used as an indicator for the position along the track, which the place field encoded for relevant subsequent analyses. For place cells that had significant bidirectional place sensitivity, several additional analyses were conducted in order to examine the relationship between the place fields exhibited in each direction. Similarity between place fields was generally quantified using the correlation between the fields. A high correlation between place fields indicates that the shape of the fields is similar and, given this, that they encode a similar location along the track. The distribution of LSPF/RSPF correlations was computed for all bidirectionally sensitive place cells that were recorded to examine the directional interactions of this population of place cells. Additional descriptors of bidirectionality were computed, including the distance between peak encoding positions, and a left/right information selectivity index:

$$ISI=\frac{I_{LSPF}-I_{RSPF}}{I_{LSPF}+I_{RSPF}}$$

(an indicator of relative differences in the location specificity of the place fields in each direction).

In order to quantify potential repeated sampling of neurons across recording days, similarity analyses were conducted on place cells recorded on the same channel during adjacent recording sessions not conducted on the same day. Similarity was evaluated using a number of different metrics that encompassed the waveform similarity, spiking statistics, and task responsiveness of neurons. Waveform similarity was evaluated by comparing the mean waveform correlation and change in peak amplitude. Spiking statistics were compared by comparing the interspike interval distribution correlation computed with a 500 ms time lag and 5 ms bins. Similarity in task responsiveness was evaluated by comparing the change in mean place field rate, change in place field width, and overall place field correlation between the 2 recording days. It is noted that only neurons recorded on the same channel of a given implant that were both identified to be place cells that had the same directional tuning properties across consecutive recording sessions were included in this analysis. The myriad of metrics used to compare neurons here was motivated by the potential for a significant amount of variability in place field tuning over longer periods of time that has been observed by multiple research groups conducting chronic recordings with the intention of retaining the same units across multiple days [90,91]. Though dissimilarity in the metrics proposed here does not provide definitive proof that tissue drag and repeated sampling of the same neurons across days did not occur, the proportion of neuron pairs that meet the inclusion criteria proposed here and retain high consistency across days can serve as an estimator for the amount of repeated sampling and possible inflation of the number of place cells that were repeatedly observed across multiple days.

### Data analysis—Bayesian decoder

The relatively large place fields observed in many of the track recordings invite the question of whether a concurrently recorded population of neurons from the hippocampus in a marmoset is sufficient to decode track position. Substantial efforts have been devoted to the development of various decoding methods that serve to demonstrate both the information content of the recorded neurophysiological signals [53–56] and, specifically when used causally to predict downstream behavior, give insight into the computational mechanisms that the brain employs to subsequently process the recorded neuronal activity [57]. In spite of the large spatial extent of place fields and relatively low recording yield of place cells, we attempted to decode marmoset position using a two-step Bayesian decoder [53]. Rather than continuously decoding 2D position as is the common practice of rodents exploring a 2D environment, individual trajectories were decoded separately in each direction, only using place fields that were spatially tuned in each trajectory’s direction of motion along the track. Thus, aggregated measures of decoding performance for each session were averaged across trajectories and across left and right directions of motion along the L track. Decoding of location was conducted by defining separate training and test recording periods for each recording session, lasting 75% of the recording and 25%, respectively. Place fields were computed from the training period of the recording as previously described in Data analysis—Neurons. Decoding was subsequently conducted using the test period, with a 0.75 sliding window and a 0.01-s step length defining the test period spikes that were used to compute the current position. Following trajectory prediction, the decoded trajectory was subsampled to align with the sampling rate and timing of the camera frames captured during actual marmoset recording, after which decoding performance was evaluated. Three different performance metrics were used to evaluate the decoder: the correlation between each true and decoded trajectory, the fraction of the variance of the true trajectory that the decoded trajectory explained, and the mean absolute error between the true and decoded trajectories reported in centimeters. This entire decoding procedure was subsequently repeated using 4-fold cross validation, with performance metrics being averaged over the folds. Statistical significance of the decoding outcome was evaluated using another variant of shuffling to generate a null distribution in which the identity of each place cell during the test period was shuffled with respect to the place field computed during the training period, thus destroying the predictive relationship of the discharged spikes with the underlying position of the animal while retaining the true spatial statistics of the computed place fields and the true temporal statistics of the discharged spikes during the test period [92].

### Data analysis—Long axis analysis

Variations in spatial information encoding as a function of position along the rostro-caudal axis of the marmoset hippocampus were evaluated post hoc by separating neurons by implant and conducting 3 separate analyses of place-field spatial extent. The first of these was a comparison of place-field width, which was computed as the width of the place field above 20% of the maximum firing rate for each place field. In cases in which multiple place fields were identified by the 20% threshold, the fields were merged together if their boundaries were within 5 cm of each other. If the multiple fields were more than 5 cm apart, the largest place-field width was taken as representative for that place cell. Evaluation of pairwise statistical significance was then conducted between the anterior, middle, and posterior implant sites. For all tests of significance in this analysis, distributions of metrics—in this case, the distribution of place-field widths for all place cells at each implant site—was quantified by Mann–Whitney U Test. The second analysis involved a comparison of the spatial information transfer rate exhibited by place cells at each location. The spatial information had already been computed as described in Data analysis—Neurons and is computed as a weighted information entropy of each neuron with respect to the environment the animal is exploring. Given this, one would expect that place fields with large spatial extent would not provide very much information about current animal position per spike discharged, whereas place fields with a very narrow firing field would be more informative about the animal’s current location. This notion was assessed by, once again, separating place cells based on recording location and conducting pairwise comparisons for the distribution of spatial information across all place cells in each location. The third analysis involved a direct assessment of spatial extent at the population level while being agnostic to the exact definition of “place field width” as we defined for the first analysis. Inspired by the analysis conducted in the work by Kjelstrup and colleagues [52], a similar analysis was conducted in which 2D population correlation matrices were constructed for each of the implanted sites. These matrices were constructed by first defining an *N* × *M* pseudopopulation matrix of place field firing rates for each implant site, where *N* is the number of place cells recorded across all sessions at a given implant site and *M* is the number of discrete bins used to calculate place fields. The Pearson correlation coefficient was the computed between every pair (*i*, *j*) of *N* × 1 “population vectors” each representing the activity of the pseudopopulation at track locations *i* and *j*, respectively. Thus, a pseudopopulation correlation matrix of size *M*^2^ was computed for each of the 3 implant sites, and the extent of track position “cross-correlation” could be quantified in a place field width-parameter agnostic manner.

### Data analysis—Nonoscillatory LFP analysis

Nonoscillatory LFP analysis was conducted to match the cycle-by-cycle variability in LFP frequency analysis detailed in Zhang and colleagues [53]. The raw LFP sampled at 3 kHz was bandpass filtered with a passband of 1 to 10 Hz, and individual cycles were demarcated by identifying the troughs in the filtered signal. Instantaneous power was also computed using the Hilbert envelope (square of the magnitude of the Hilbert-transformed LFP) and was averaged within the time-limits of each cycle to compute the mean power of each cycle. The bottom 25th percentile of cycles by mean power were then discarded to mitigate noise-intrusion on the nonoscillatory LFP cycles. The remaining cycles were then linked into bouts as follows: the bottom 50th percentile of cycles ranked by mean power was again discarded, and this final subset of cycles was subjected to linking wherein pairs of cycles that were separated by no longer than their mean duration were assigned to the same bout. Following linkage, only bouts with 3 or more cycles present were retained for subsequent analysis. Statistics related to these bouts were computed, including the distribution of durations and mean frequencies computed as the average frequency of all cycles included in individual bouts. In order to assess the agreement in spectral content between these wide-band LFP bouts and the θ bouts identified by MODAL; the fraction of bouts per channel whose mean frequency content fell within the band limits of the MODAL-identified θ oscillation were also quantified. To assess behaviorally relevant modulation of these nonoscillatory LFP fluctuations, the nonoscillatory bouts were divided into HV and LV groups in a manner identical to that employed for the MODAL bouts, and differences in mean bout frequency were computed between the HV and LV groups, both within individual channels and averaged across recordings.

### Data analysis—Spike-field interactions

To probe for θ-phase coding of place cells during locomotion, 2 separate general approaches were used; one involving the computed θ oscillations and one strictly involving intrinsic spiking dynamics of place cells. Intrinsic oscillatory modulation of place cells was assessed independently of estimated θ oscillations using a previously developed approach [3]. In brief, the autocorrelation function (ACF) of each recorded place cell (symmetric 2 s window, 10 ms bins) is computed separately for periods of LV and HV locomotion in the direction of motion for which there is a place field. The power spectrum for each of these ACFs was then computed with a 0 to 50 Hz bandwidth and 0.5 Hz frequency resolution given the selected ACF parameters. θ modulation was identified by comparing the spectral power in a broadly defined θ band (5–12 Hz) during the low and HV motion conditions. The θ index was computed as an asymmetry index using the integrated power across the θ band for the 2 conditions:

$$\theta -index=\frac{\theta_{HV}-\theta_{LV}}{\theta_{HV}+\theta_{LV}}.$$

Statistical significance of the θ index was assessed using a similar permutation bootstrap technique to that previously described for the information index. The spike times for each unit were randomly circularly shifted past the marmoset’s location and the θ index recomputed *N* = 1,000 times to generate a null distribution of θ indices for each place cells. The *p*-value was computed accordingly.

The interaction between single-unit spike timing and the local field was quantified by determining the instantaneous LFP phase during which each spike occurred using the estimated phase. The PLV was then computed from these phases and was used as an indicator of spike-field synchrony. The PLV was computed as the sum of the phases of the θ oscillation at which spikes were discharged [93]. These phases were treated as phasors and mapped onto the unit circle per the definition of the PLV, giving rise to the calculation:

$$PLV=\frac{1}{N}\sum_{n=1}^{N}e^{j\varphi_{n}},$$

where *φ*_*n*_ is the θ phase at which the *n*th spike discharged for a given neuron during recording. Statistical significance of the PLV for a given single unit was demonstrated by generating a surrogate PLV distribution of random, uniformly distributed circular shifts of the spike-timing data with respect to the LFP. Because of the intermittent nature of the θ oscillation, only spikes that occurred both during HV locomotion in the direction of place tuning and during bouts of θ were included in this analysis. Simple phase locking of place cells to θ was evaluated by binning spike phases on the interval [0,2*π*]) for those units that exhibited significant PLV using the above approach. In addition, phase precession of spiking through the θ oscillation was evaluated by computing a linear-circular correlation [94], relating spike phases during locomotion through the place field to the position of the marmoset within the place field. With this method of circular correlation, a formula for the analytical *p*-value had been derived, thus allowing for direct computation of significance without the need for surrogate statistics.

### Data analysis—θ sequence analysis

Data were also evaluated for the presence of θ sequences by employing a comparative cross-correlogram approach as previously described [21,68]. The CCG between pairs of concurrently recorded place cells was computed at 2 different time scales, the time scale of the θ oscillation and the time scale of behavior. The θ-CCG was computed with a 125 ms latency and a 1 ms bin width, after which the CCG envelope was smoothed with a third order low-pass Butterworth filter with a 40 Hz cutoff. The behavior-CCG was computed with a 1,000 ms latency and a 3 ms bin width, after which the CCG envelope was smoothed with a third order low-pass Butterworth filter with a 1.5 Hz cutoff. Each CCG peak was only classified as significant and included in subsequent analysis if the peak amplitude exceeded 0.5 Hz and the z-scored peak amplitude exceeded 1.5. The relationship between CCG peak latency and place-field distance was quantified by computing the Pearson’s correlation coefficient separately between the θ-CCG peak latencies pooled across all neuron pairs in all recordings and the place field separation, computed as the distance in centimeters between the place-field peaks for the pair of neurons in question. This same correlation was also computed between the behavior-CCG and the place-field separation across all recording sessions. For reference, the distance between the peaks of the 2 place fields was also computed and used as a sanity check. It should always be the case that, for 2 place cells with spatially adjacent fields, a strong association exists between the maximal behavioral-CCG time lag and the distance separating the place-field peaks. However, in the presence of significant temporally organized θ sequences, a strong correlation will also exist between the maximal θ-CCG time lag and the place-field distance, because the temporal organization of the θ sequence will reflect the spatial sequence in which adjacent place fields are traversed [69]. Thus, the correlation between the θ-CCG latency and behavioral-CCG latency was computed for all pairs of neurons within all recording sessions. A further analysis was conducted in which all CCGs were recomputed only using spikes that occurred during periods of significant θ bouting, and the correlation between CCG peak latency and place-field distance was reassessed with the correlation coefficient as previously described.

### Data analysis—LFP-driven place fields

In order to determine whether the presence of θ bouting during locomotion was influencing the spatial representations of place cells, spikes discharged during HV locomotion were segregated into those that occurred during a θ bout (θ+) and those that did not (θ−). The consistency of the place field between the 2 conditions was initially quantified by correlation. Changes in the encoded location were then computed by comparing deviations in the center of mass of each place field with respect to the full place field (θ+/−). Since the θ+ and θ− place fields are effectively subsampling the full recording, any changes in location encoding equally reflected in the θ+ and θ− fields with respect to the θ+/− field are not due to the presence or absence of θ bouting, and the distributions of ΔCOM_θ+_ and ΔCOM_θ−_ were compared via KS-test to determine whether this was the case. Finally, the spatial information rate for the θ+ and θ− place fields of each place cell was computed to examine differences in the total information conveyed by the population to downstream neurons during locomotion.

Data have been deposited in the Dryad repository: https://doi.org/10.5061/dryad.kk63d49 [95].

## Supporting information

S1 Fig Spatial encoding in the marmoset hippocampus in a linear track. The first neurons exhibiting spatial selectivity in the marmoset hippocampus were recorded on a 2.4-m linear track over the course of 2 recording sessions conducted on separate days (peak FR = 8.47 ± 2.96 Hz, field size = 141.85 ± 45.96 cm, FR modulation index = 0.73 ± 0.09, Skaggs–McNaughton information = 0.60 ± 0.26 bits/spike). Normalized maximum firing rates on a linear track for individual exemplar neurons are shown for cells exhibiting spatial selectivity during (A) left-moving travel and (B) right-moving travel. (C) Three individual exemplar neurons recorded in the linear track environment. The upper left plots the spike waveform. The mean (dark black line) and 2 SEM (dashed black lines) are shown for each waveform. Lower left plots the interspike interval distributions for each neuron. The upper right plots the individual travel trajectories for each test session (x-axis plots the position on the track, and the y-axis plots time in seconds). Green line plots left-moving travel, and the blue lines indicate right-moving travel. Red dots plot the occurrence of an action potential during locomotion. The lower right plots a histogram of the spatial position of action potentials for each neuron, distinguishing between left-moving (green) and right-moving (red) travel during HV movement (>20 cm/s). FR, Firing Rate; HV, high velocity.
(TIF)

S2 Fig Surgical outcomes for the 3 implanted hemispheres. The left implant of subject BL is shown in (A), and the left and right implants of subject TD are shown in (B) and (C), respectively. Localization information for these implants is given in S1 Table.
(TIF)

S3 Fig Axial views for each of the 3 projected implant volumes shown coronally in S2 Fig. Axial slices were taken from the maximal cross-sectional area through each implanted volume and overlaid on the RIKEN marmoset atlas NISSL stain to visualize DG/CA field coverage. Each full-brain visualization (A, C, E) is paired with a zoomed view (B, D, F) to highlight the region of MBA coverage for each implant. The color-coding scheme matches that used in S8 Fig with blue = TD-R, green = TD-L, and red = BL-L. Quantification of volumetric subfield coverage is documented in S2 Table. BL-L, Male subject (BL) Left Hemisphere; CA, Cornu ammonis; DG: Dentate Gyrus,; MBA, microwire brush array; TD-L, Female subject (TD) Left Hemisphere; TD-R, Female subjects (TD) Right Hemisphere.
(TIF)

S4 Fig Analysis of marmoset behavior on the L track. (A) Histograms of the total time the subject spent on the long and short arms of the track is shown for all 37 recording sessions. Time is aggregated across both HV and LV periods on each arm. (B) Histograms of mean velocity of the subject on each arm of the track is shown for all 37 recording sessions. Instantaneous velocity was separately computed across each recording session, separated by track arm (short/long), then averaged to yield the final mean velocity for each track arm and session. (C) Histograms of mean velocity of the subject traveling in each direction along the track. Instantaneous velocity, as computed in panel B, was segmented by direction of travel, left or right, and the mean computed for each direction of travel across both arms of the track for each recording session. Occupancy times for each recording are reported in seconds. All velocities are reported in centimeters per second. All reported *p*-values above were computed by paired signed-rank test between the 2 histograms shown in the figure, i.e., long versus short arm in panels A and B and left versus right in panel C. HV, high velocity; LV, low velocity.
(TIF)

S5 Fig An example of the clustering outcome of a single channel in which 3 distinct spike clusters were identified. The waveforms for each of the 3 isolated spike clusters are shown in panels A through C, with the associated clustering feature space shown in 2D projections in panel D. The time and amplitude scale of each waveform cluster in panels A through C is shown to the right of its associated waveform. Within the feature spaces in panel D, the blue cluster represents the waveforms of panel A, the yellow cluster represents the waveforms in panel B, and the cyan cluster represents the waveforms in panel C. The top row of feature spaces in panel D shows the projection of all spikes detected on this channel onto the top 3 principal components shown in pairs from left to right: PC1 × PC2, PC2 × PC3, PC1 × PC3. The bottom row of panel D shows the projection of spikes onto the maximally non-Gaussian wavelet coefficients (MW) shown in pairs from left to right: MW1 × MW2, MW2 × MW3, MW1 × MW3. Clustering during spike sorting was conducted on the full 6-dimensional feature space. MW, maximum non-Gaussian wavelent coefficient; PC, principal component.
(TIF)

S6 Fig The distribution of mean firing rate over the course of the recording, spatial information rate encoded on the track, and spatial information per spike discharged are reported for all neurons classified as putative place cells. (A) Distribution of the mean firing rate for each neuron recorded. The mean firing rate was computed as the total number of spikes discharged over the course of the recording including both HV and LV periods. (B) Distribution of the mean information rate for all neurons recorded. The mean information rate is an indicator of the amount of information per unit time being transmitted about current track position by a given place cell to downstream neurons. This value was determined by dividing the amount of information carried per spike discharged. (C) The distribution of mean information of the place cell population plotted with the same conventions as in panel B. HV, high velocity; LV, low velocity.
(TIF)

S7 Fig Additional exemplar place cell activity on the L track. Place cells are separated according to directional selectivity with (A) RSPF, (B) LSPF, and (C) bidirectionally selective cells all being observed. For each individual neuron, the top plot shows individual travel trajectories for each test session (x-axis plots the position on the track, and y-axis plots time in seconds). Green line indicates left-moving travel, and blue lines indicate right-moving travel. Red dots plot the occurrence of an action potential during locomotion. Bottoms plots are a histogram of the spatial position of action potentials for each neuron, distinguishing between left-moving (green) and right-moving (red) travel during HV movement (>20 cm/sec). The vertical dashed line plots the 90° turn in the L track. HV, high velocity; LSPF, left-selective place field; RSPF, right-selective place field.
(TIF)

S8 Fig Example of a high-yield recording session in which place cells recorded simultaneously represented the entirety of the recording environment. The image on the left plots the firing rate by the position along the L track for individual LSPF in blue, and the image on the right plots RSPF. Each distinct place field subplot corresponds to the activity of a single neuron when traveling in the respective direction. Place-cell numbering corresponds to the sorted order of peak position from the origin and in the direction of travel, i.e., 0 to 427 cm for blue and 427 to 0 cm for green. LSPF, left-selective place field; RSPF, right-selective place field.
(TIF)

S9 Fig Co-localization of the initially implanted volume of tissue across all 3 subjects. The volume of implantation is defined as the maximum possible volume of splay around the implant site, i.e., a sphere with 1 mm radius. The red volume corresponds to the implant on the left hemisphere of subject BL, the green corresponds to the left implant of subject TD, and the blue corresponds to the right implant of subject TD. The long axis of hippocampus was defined by computing the principal axis of all voxels in the anatomical atlas defined as “HPC.” The colored lines in the long axis position plot are the 1D projections of the volumes, with the red line (BL-L implant) being the most anterior, and the blue line (TD-R implant) being the most posterior. Black vertical lines indicate AP position of the cross-sectional slices of the T2-weighted MRI shown above. Note that the conventions for our animal scanner have image left corresponding to subject left. AP, Anterior Posterior.
(TIF)

S10 Fig Evaluation of the MODAL algorithm on simulated LFP data with inserted θ bouts. (A) The distribution of estimated θ-bout SNR for all recording channels that had a significant θ oscillations. The Sens and FPR (1 − specificity) are shown for bouts simulated at 6 Hz, 8 Hz, and 10 Hz in panels B, C, and D, respectively. Blue and orange bars in these figures correspond to the Sens and FPR of MODAL for the reported bout parameters, e.g., 100% Sens and 17% FPR for 0.3 s bouts with a mean frequency of 6 Hz for the left-most entry of panel B. The x-axes for panels B through D are bout durations for individual simulated runs of MODAL reported in seconds, and the y-axes are percentages for Sens and FPR. FPR, false positive rate; LFP, local field potential; MODAL, Multiple Oscillation Detection Algorithm; Sens, sensitivity; SNR,signal-to-noise ratio.
(TIF)

S11 Fig Distributions of θ-bout power comparing HV (purple shading) and LV (blue shading) separated by recording day. Bout power is plotted on the x-axis in normalized units, and total number of bouts exhibiting each power level by bin is plotted on the y-axis. The *p*-values reported for each day are indicative of the significance of the difference between the HV and LV duration distributions using the KS-Test. Only *p*-values that exceeded the *α* = 0.01 threshold are indicated. HV, high velocity; KS, Kolmogorov-Smirnov Test; LV, low velocity.
(TIF)

S12 Fig Distributions of θ-bout durations comparing HV (purple shading) and LV (light shading) separated by recording day. Bout duration is plotted on the x-axis in seconds, and total number of bouts exhibiting each duration by bin is plotted on the y-axis. The *p*-values reported for each day are indicative of the significance of the difference between the HV and LV duration distributions using the KS-Test. Only *p*-values that exceeded the *α* = 0.01 threshold are indicated. HV, high velocity; KS, Kolmogorov-Smirnov Test; LV, low velocity.
(TIF)

S13 Fig CCG analysis was conducted comparing peak CCG latency to peak place-field distance to probe for θ sequences and further probe for evidence of phase precession. The distance between place-field peaks (x-axis) is plotted against the peak CCG latency for all pairs of units recorded on the same day (y-axis), with each point corresponding to a single place-cell pair. Overlaid red lines indicate the least-square fit of the data with associated Pearson correlation coefficient (ρ) and explained variance (R^2^) of the fit reported above each plot. (A) Plots of the CCG using the entire recording at the θ time scale. (B) Plots of the CCG using the entire recording at the behavioral time scale. (C) Plots of the CCG construction for the θ time scale but limited to spikes that occurred during θ bouts. (D) Plots of the CCG construction for the behavioral timescale but limited to spikes that occurred during θ bouts. All linear fits computed above were highly statistically significant (*p* < 10^−10^ in all cases), but the explained variance for the θ time scale was miniscule (7% = A, 5% = C). CCG, cross-correlogram.
(TIF)

S14 Fig Similarity analysis is shown between pairs of neurons recorded on consecutive recording days punctuated by movement of the MBA. Neuron pairs shown here were on the same channel and exhibited the same place tuning across the different recording sessions. The similarity metrics shown are (A) the change in spike waveform amplitude, (B) the mean waveform correlation between pairs, (C) the correlation of the unsmoothed interspike-interval distribution with 500 ms latency and 5 ms bins. The remaining metrics focused on the place field: (D) place field correlation, (E) the change in peak firing rate within the place field, and (F) the change in width of the place field defined by 20% of max firing rate. For the place field metrics, directionally tuned place fields were matched (i.e., a bidirectionally tuned cell contributed twice to each of these metrics but only once to the metrics shown in panels A through C. MBA, microwire brush array.
(TIF)

S15 Fig The power spectrum for each channel on which a significant θ oscillation was detected by the MODAL algorithm is shown for the fitted frequency range (3–25 Hz). Individual plots are shown with increasing frequency on the x-axis and normalized power on the y-axis. Note spectra in these plots were normalized to the power at the 0Hz frequency of thepower spectrum (DC) to facilitate comparison between channels by visual inspection. The channels here are pooled across all 3 subjects and are sorted in order of increasing peak θ frequency, with the title number being an arbitrary total order for the channels postsorting. The identified θ band was not demarcated to facilitate reading of the estimated spectra but in all cases is centered on the peak consistently found between 5 and 10 Hz across all recordings. (A) Ch 1:45, (B) Ch 46:90, (C) Ch 91:135, (D) Ch 136:155. DC, direct current; MODAL, Multiple Oscillation Detection Algorithm.
(TIF)

S1 Table Targeting information for implanting MBAs into the marmoset hippocampus. The AP axis is computed with respect to the center of the interaural canal, which was clearly identifiable in all pre- and postoperative MRIs. The medio-lateral axis distance is computed with respect to the cerebral fissure. The declination is reported as angular deviation in degrees from true vertical. Depth is computed as linear distance advanced from the surface of cortex to the end of the leading edge of the guide tube within hippocampus. Note that, during postoperative scanning, the MBA had already been advanced 250 μm beyond the leading edge, though splaying of the individual electrode tips cannot be visualized because of isotropic spatial resolution limitations. AP, Anterior Posterior; MBA, microwire brush array.
(TIF)

S2 Table Relative hippocampal subfield coverage estimated from post-implant T2-weighted MRI. The first column indicates the identity of each implant ordered from posterior to anterior, and the associated row indicates the fraction of pyramidal/granular layer voxels within hippocampus that the implant contains. Percentages for each implant are computed separately based on number of voxels falling within the pyramidal layer of each CA field and the granular layer of the dentate gyrus divided by the total number of voxels from all CA-field pyramidal layers within the hippocampus as indicated by the Riken T2-weighted anatomical atlas aligned with the included NISSL stain. CA, cornu ammonis.
(TIF)

S3 Table Recording yield information for each location accumulated across the recording lifetime of each implant. The implants are reported in order from anterior to posterior according to their associated ROI’s projection onto the hippocampal long axis (see S8 Fig for details). All of the units recorded from a given MBA implant site are reported in the “All Units” column. The absolute number of place cells at the same location is reported in the “Place Cells (#)” column, and the ratio of the 2 is reported in the “Place Cells (%)” column. It is noted that for these counts, units intentionally recorded on the same day for stability analyses were included but not double or triple counted across the repeated sessions. MBA, microwire brush array; ROI, region of interest.
(TIF)

## Abbreviations

ACF: autocorrelation function
AM: Anterior-Middle
AP: Anterior Posterior
BT: binomial test
CA: Cornu Ammonis
CCG: cross-correlogram
EEG: electroencephalogram
HV: high velocity
ID: isolation distance
LFP: local field potential
LSPF: left-selective place field
LV: low velocity
MBA: microwire brush array
MODAL: Multiple Oscillation Detection Algorithm
MP: Middle-Posterior
PC: principal component
PF: place field
PLV: phase locking value
PMI: power modulation index
RSPF: right-selective place field
SNR: signal to noise ratio
TSE: Turbo Spin Echo
