# Supplementary figures and images for: Spatial encoding in primate hippocampus during free navigation
Source: PLoS Biol. 2019 Dec 9;17(12):e3000546. doi: 10.1371/journal.pbio.3000546 (PMC6922474; doi:10.1371/journal.pbio.3000546)

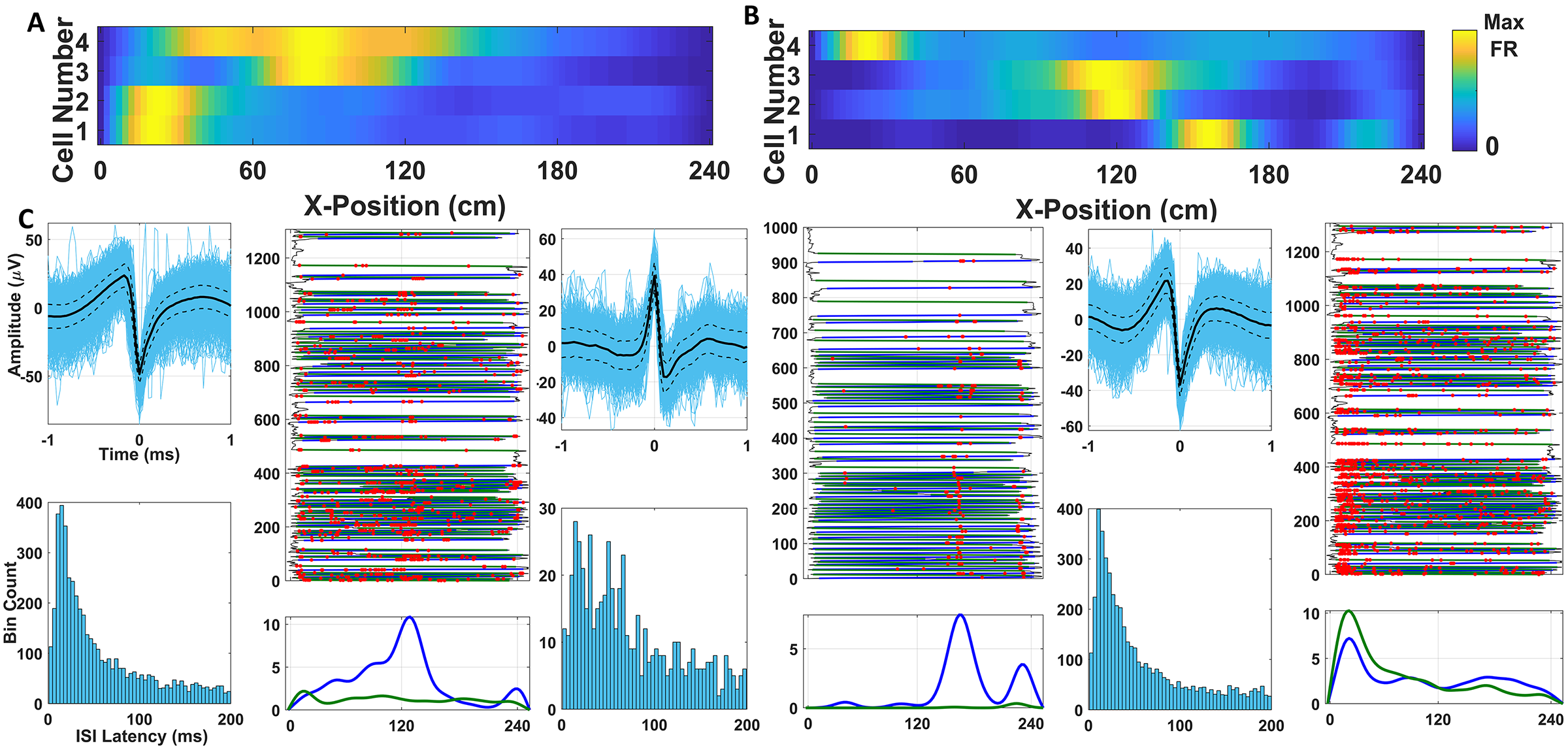

Supplement: S1 Fig — The first neurons exhibiting spatial selectivity in the marmoset hippocampus were recorded on a 2.4-m linear track over the course of 2 recording sessions conducted on separate days (peak FR = 8.47 ± 2.96 Hz, field size = 141.85 ± 45.96 cm, FR modulation index = 0.73 ± 0.09, Skaggs–McNaughton information = 0.60 ± 0.26 bits/spike). Normalized maximum firing rates on a linear track for individual exemplar neurons are shown for cells exhibiting spatial selectivity during (A) left-moving travel and (B) right-moving travel. (C) Three individual exemplar neurons recorded in the linear track environment. The upper left plots the spike waveform. The mean (dark black line) and 2 SEM (dashed black lines) are shown for each waveform. Lower left plots the interspike interval distributions for each neuron. The upper right plots the individual travel trajectories for each test session (x-axis plots the position on the track, and the y-axis plots time in seconds). Green line plots left-moving travel, and the blue lines indicate right-moving travel. Red dots plot the occurrence of an action potential during locomotion. The lower right plots a histogram of the spatial position of action potentials for each neuron, distinguishing between left-moving (green) and right-moving (red) travel during HV movement (>20 cm/s). FR, Firing Rate; HV, high velocity. (TIF) [file pbio.3000546.s001.tif]

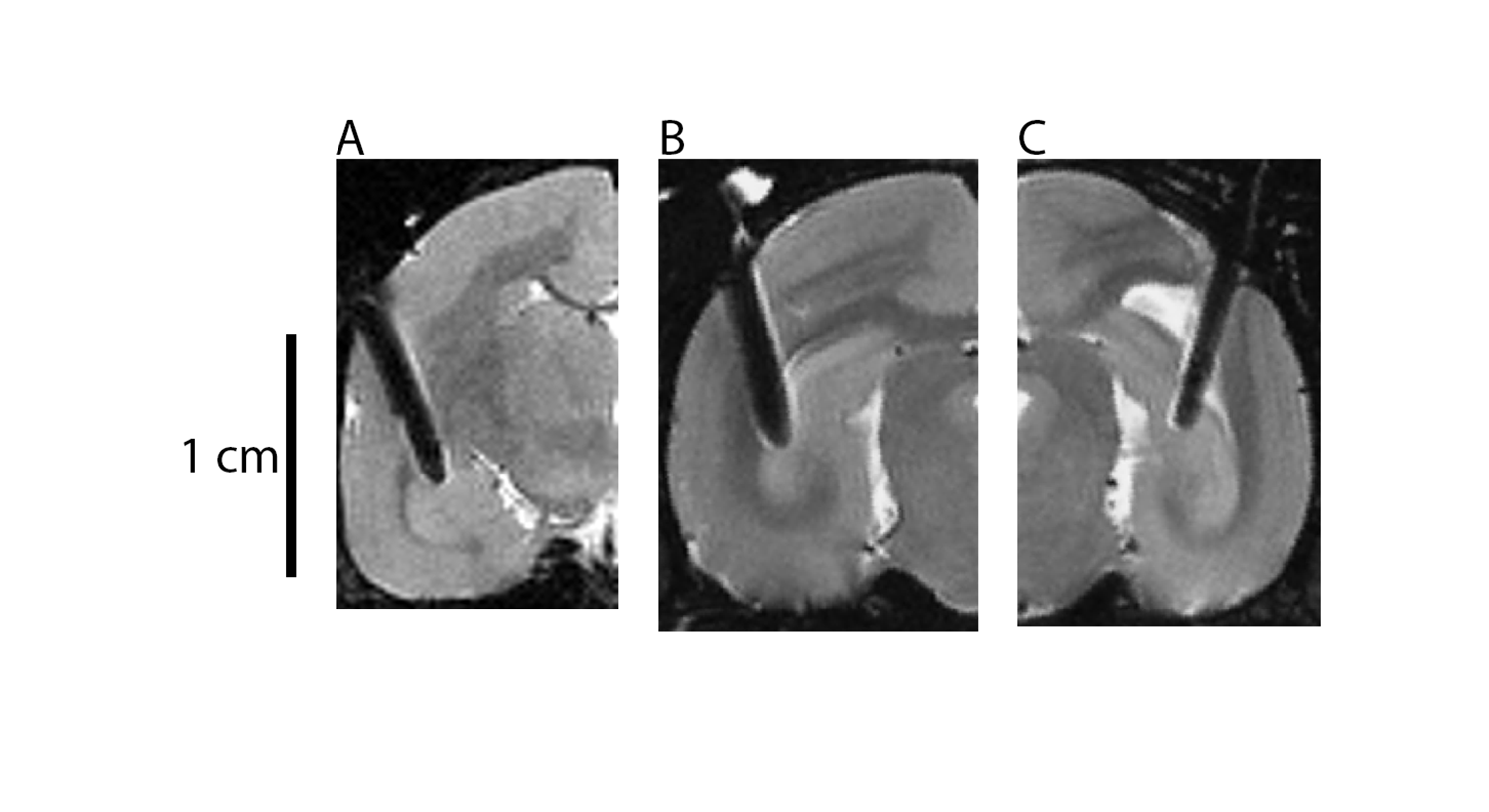

Supplement: S2 Fig — The left implant of subject BL is shown in (A), and the left and right implants of subject TD are shown in (B) and (C), respectively. Localization information for these implants is given in S1 Table. (TIF) [file pbio.3000546.s002.tif]

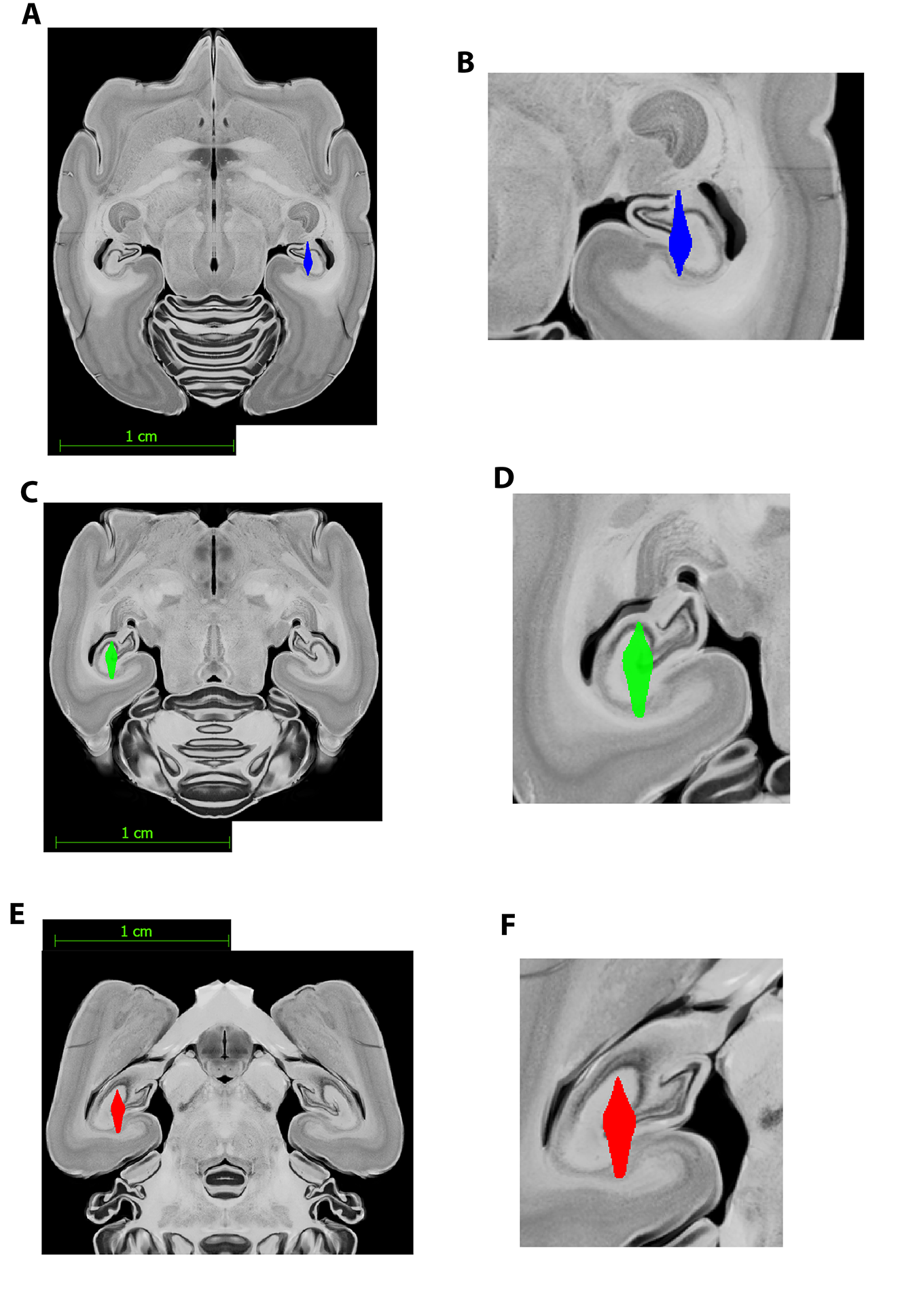

Supplement: S3 Fig — Axial slices were taken from the maximal cross-sectional area through each implanted volume and overlaid on the RIKEN marmoset atlas NISSL stain to visualize DG/CA field coverage. Each full-brain visualization (A, C, E) is paired with a zoomed view (B, D, F) to highlight the region of MBA coverage for each implant. The color-coding scheme matches that used in S8 Fig with blue = TD-R, green = TD-L, and red = BL-L. Quantification of volumetric subfield coverage is documented in S2 Table. BL-L, Male subject (BL) Left Hemisphere; CA, Cornu ammonis; DG: Dentate Gyrus,; MBA, microwire brush array; TD-L, Female subject (TD) Left Hemisphere; TD-R, Female subjects (TD) Right Hemisphere. (TIF) [file pbio.3000546.s003.tif]

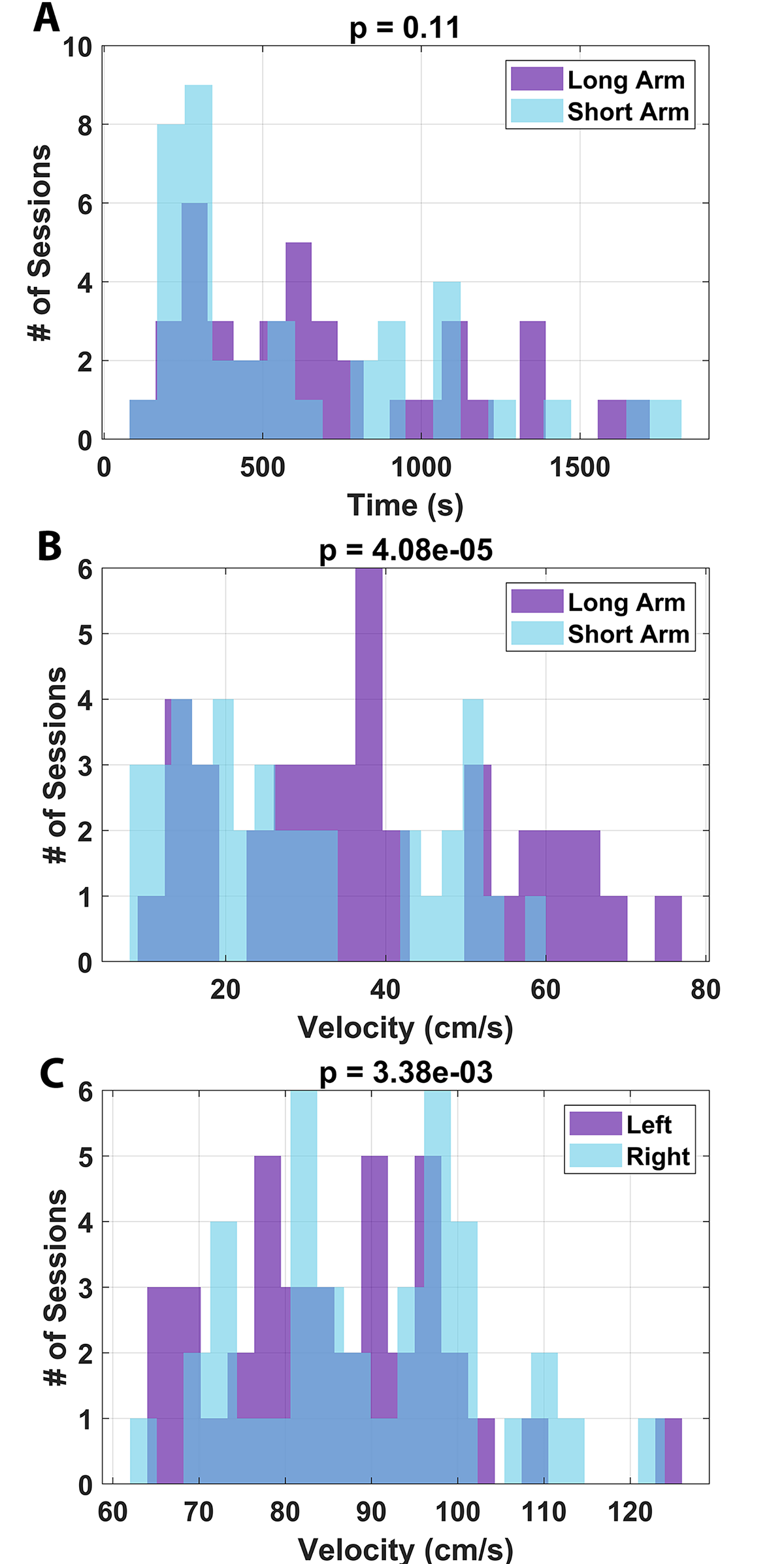

Supplement: S4 Fig — (A) Histograms of the total time the subject spent on the long and short arms of the track is shown for all 37 recording sessions. Time is aggregated across both HV and LV periods on each arm. (B) Histograms of mean velocity of the subject on each arm of the track is shown for all 37 recording sessions. Instantaneous velocity was separately computed across each recording session, separated by track arm (short/long), then averaged to yield the final mean velocity for each track arm and session. (C) Histograms of mean velocity of the subject traveling in each direction along the track. Instantaneous velocity, as computed in panel B, was segmented by direction of travel, left or right, and the mean computed for each direction of travel across both arms of the track for each recording session. Occupancy times for each recording are reported in seconds. All velocities are reported in centimeters per second. All reported p-values above were computed by paired signed-rank test between the 2 histograms shown in the figure, i.e., long versus short arm in panels A and B and left versus right in panel C. HV, high velocity; LV, low velocity. (TIF) [file pbio.3000546.s004.tif]

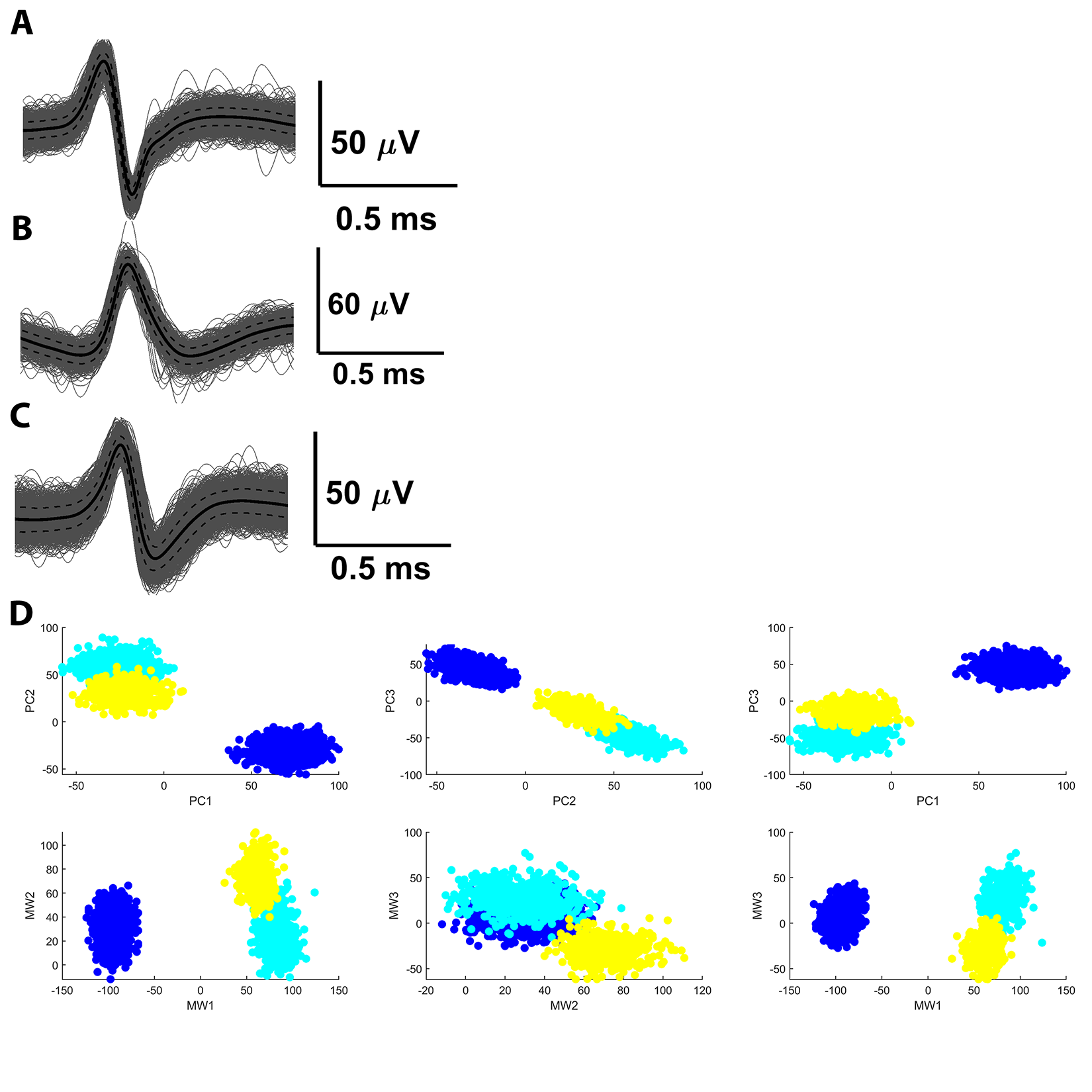

Supplement: S5 Fig — The waveforms for each of the 3 isolated spike clusters are shown in panels A through C, with the associated clustering feature space shown in 2D projections in panel D. The time and amplitude scale of each waveform cluster in panels A through C is shown to the right of its associated waveform. Within the feature spaces in panel D, the blue cluster represents the waveforms of panel A, the yellow cluster represents the waveforms in panel B, and the cyan cluster represents the waveforms in panel C. The top row of feature spaces in panel D shows the projection of all spikes detected on this channel onto the top 3 principal components shown in pairs from left to right: PC1 × PC2, PC2 × PC3, PC1 × PC3. The bottom row of panel D shows the projection of spikes onto the maximally non-Gaussian wavelet coefficients (MW) shown in pairs from left to right: MW1 × MW2, MW2 × MW3, MW1 × MW3. Clustering during spike sorting was conducted on the full 6-dimensional feature space. MW, maximum non-Gaussian wavelent coefficient; PC, principal component. (TIF) [file pbio.3000546.s005.tif]

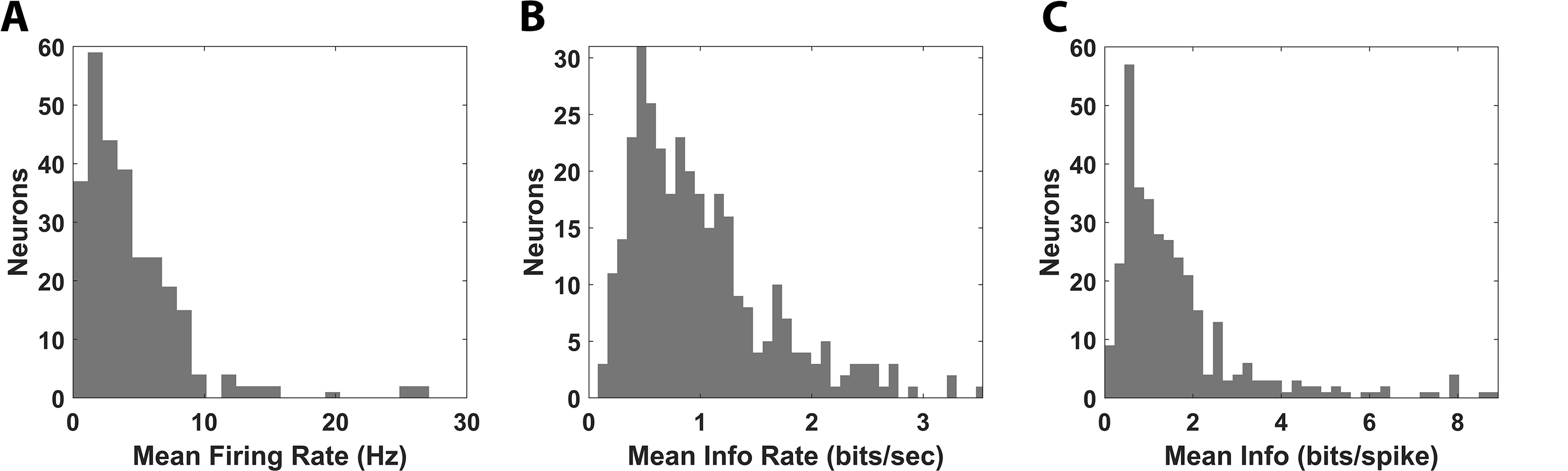

Supplement: S6 Fig — (A) Distribution of the mean firing rate for each neuron recorded. The mean firing rate was computed as the total number of spikes discharged over the course of the recording including both HV and LV periods. (B) Distribution of the mean information rate for all neurons recorded. The mean information rate is an indicator of the amount of information per unit time being transmitted about current track position by a given place cell to downstream neurons. This value was determined by dividing the amount of information carried per spike discharged. (C) The distribution of mean information of the place cell population plotted with the same conventions as in panel B. HV, high velocity; LV, low velocity. (TIF) [file pbio.3000546.s006.tif]

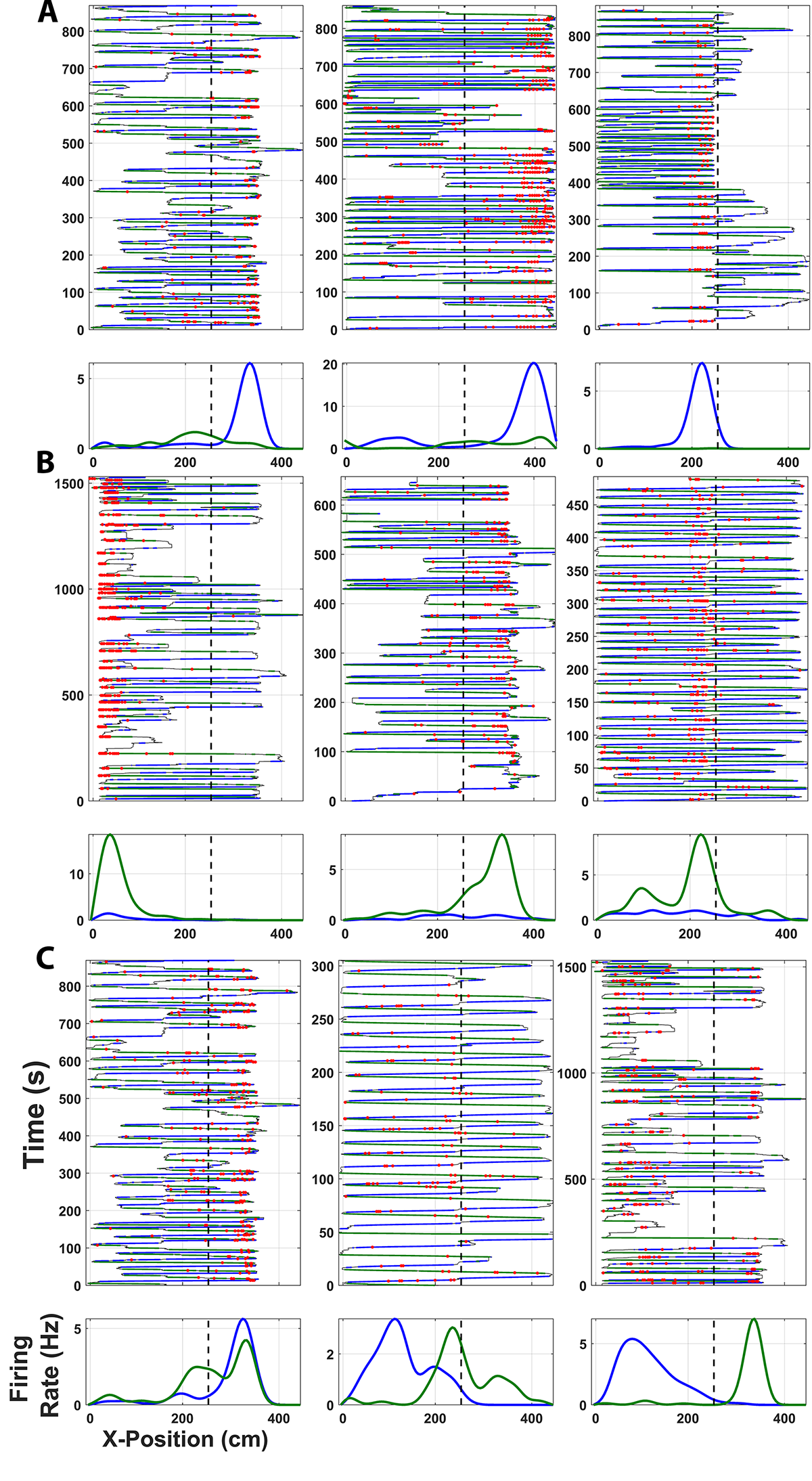

Supplement: S7 Fig — Place cells are separated according to directional selectivity with (A) RSPF, (B) LSPF, and (C) bidirectionally selective cells all being observed. For each individual neuron, the top plot shows individual travel trajectories for each test session (x-axis plots the position on the track, and y-axis plots time in seconds). Green line indicates left-moving travel, and blue lines indicate right-moving travel. Red dots plot the occurrence of an action potential during locomotion. Bottoms plots are a histogram of the spatial position of action potentials for each neuron, distinguishing between left-moving (green) and right-moving (red) travel during HV movement (>20 cm/sec). The vertical dashed line plots the 90° turn in the L track. HV, high velocity; LSPF, left-selective place field; RSPF, right-selective place field. (TIF) [file pbio.3000546.s007.tif]

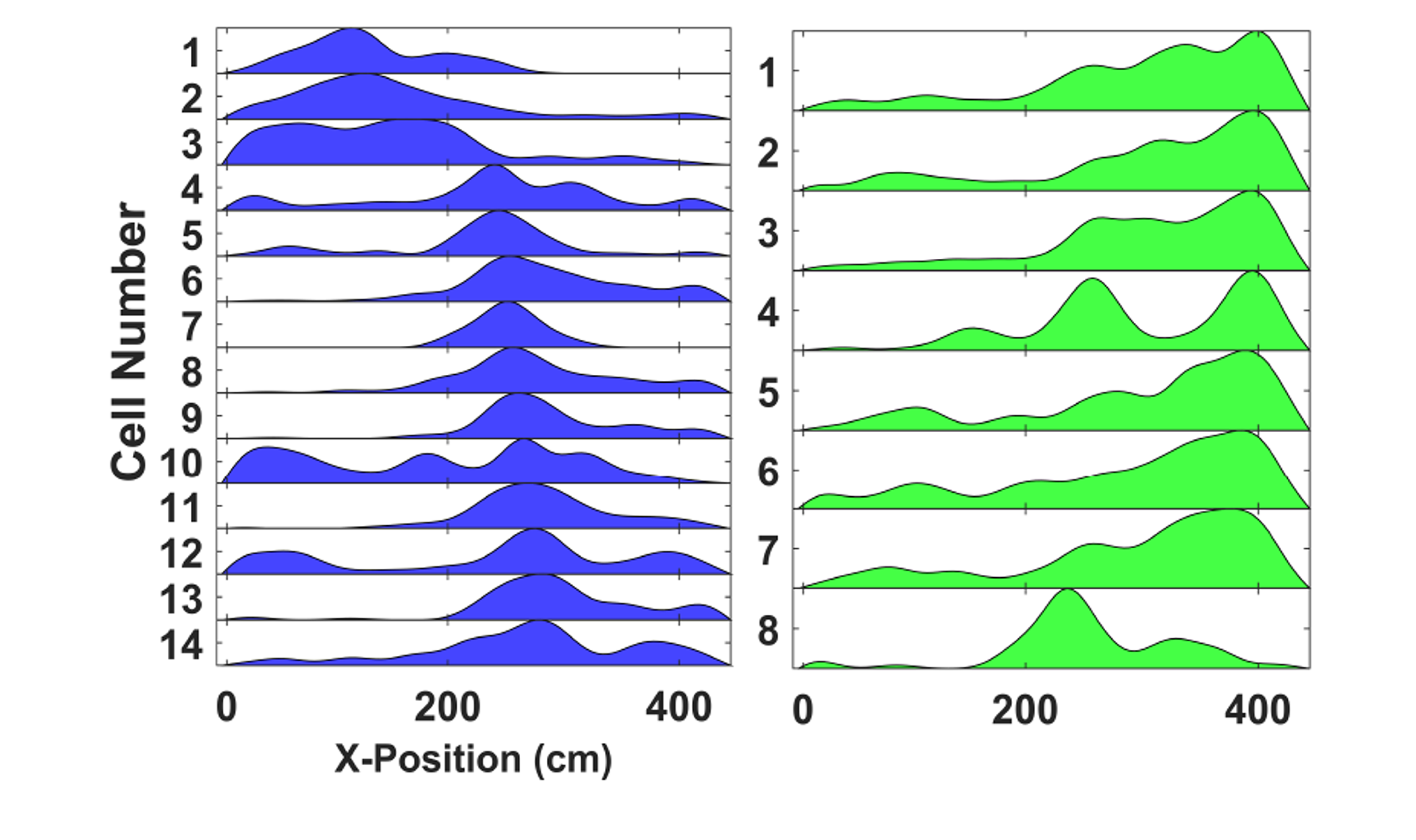

Supplement: S8 Fig — The image on the left plots the firing rate by the position along the L track for individual LSPF in blue, and the image on the right plots RSPF. Each distinct place field subplot corresponds to the activity of a single neuron when traveling in the respective direction. Place-cell numbering corresponds to the sorted order of peak position from the origin and in the direction of travel, i.e., 0 to 427 cm for blue and 427 to 0 cm for green. LSPF, left-selective place field; RSPF, right-selective place field. (TIF) [file pbio.3000546.s008.tif]

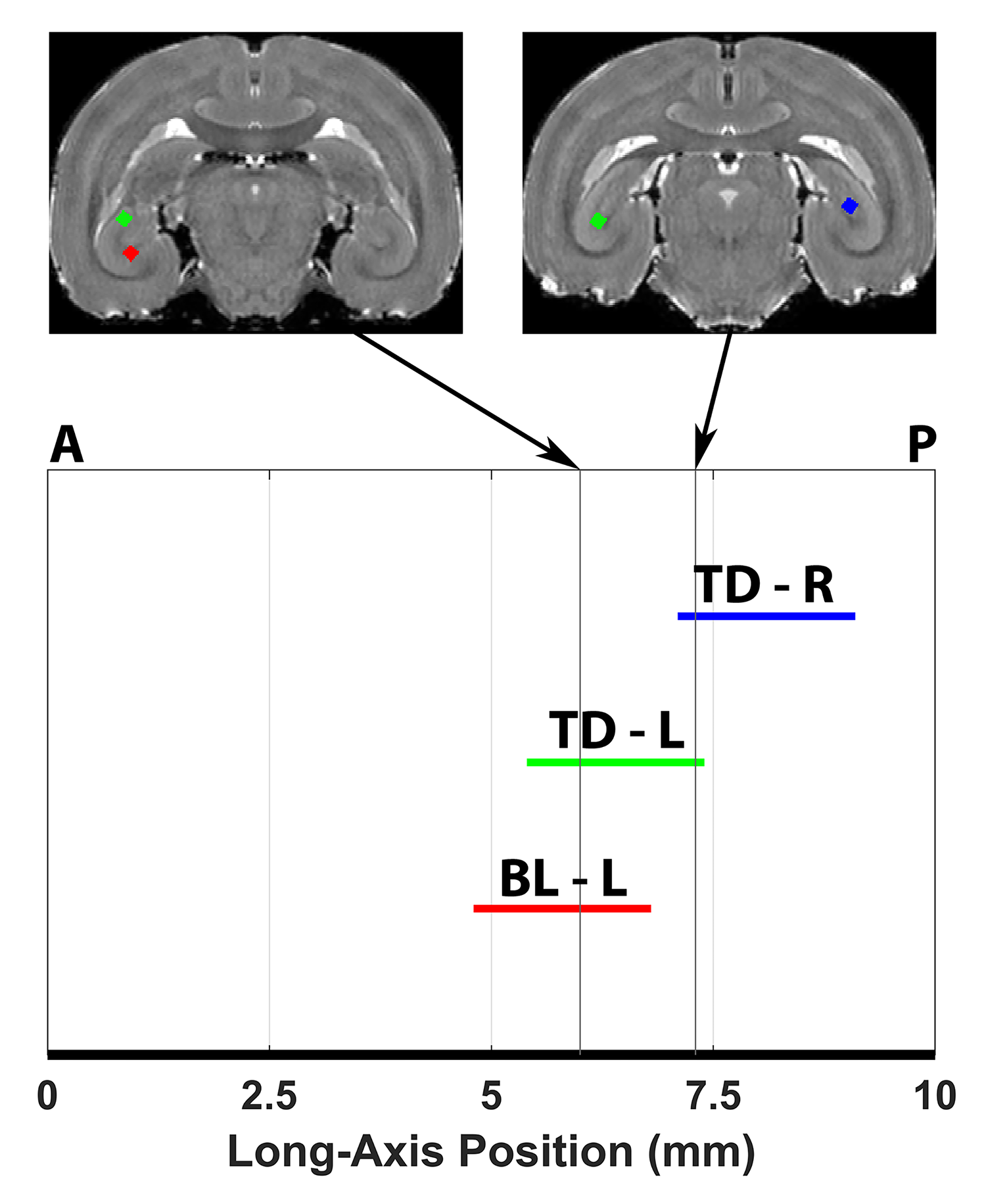

Supplement: S9 Fig — The volume of implantation is defined as the maximum possible volume of splay around the implant site, i.e., a sphere with 1 mm radius. The red volume corresponds to the implant on the left hemisphere of subject BL, the green corresponds to the left implant of subject TD, and the blue corresponds to the right implant of subject TD. The long axis of hippocampus was defined by computing the principal axis of all voxels in the anatomical atlas defined as “HPC.” The colored lines in the long axis position plot are the 1D projections of the volumes, with the red line (BL-L implant) being the most anterior, and the blue line (TD-R implant) being the most posterior. Black vertical lines indicate AP position of the cross-sectional slices of the T2-weighted MRI shown above. Note that the conventions for our animal scanner have image left corresponding to subject left. AP, Anterior Posterior. (TIF) [file pbio.3000546.s009.tif]

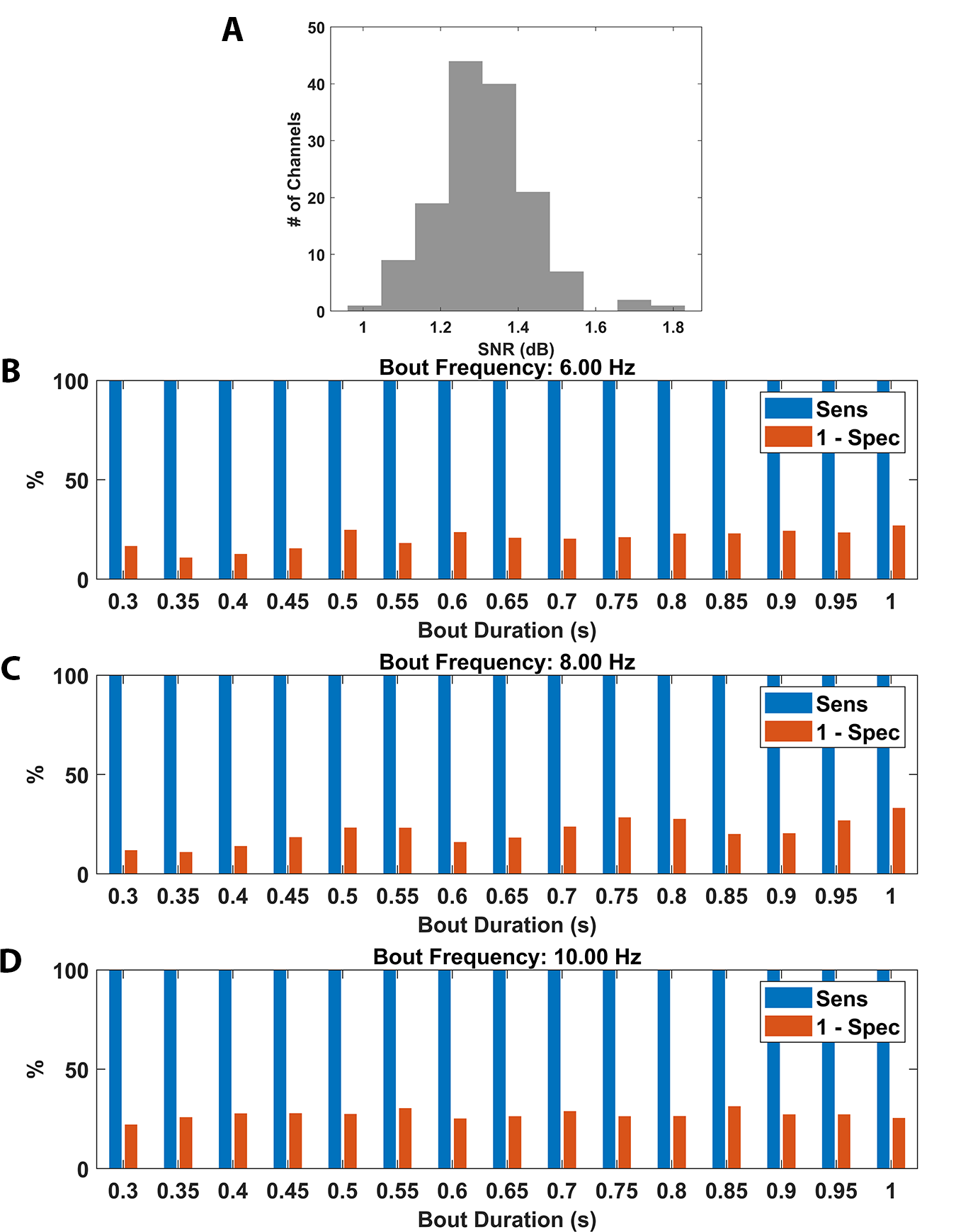

Supplement: S10 Fig — (A) The distribution of estimated θ-bout SNR for all recording channels that had a significant θ oscillations. The Sens and FPR (1 − specificity) are shown for bouts simulated at 6 Hz, 8 Hz, and 10 Hz in panels B, C, and D, respectively. Blue and orange bars in these figures correspond to the Sens and FPR of MODAL for the reported bout parameters, e.g., 100% Sens and 17% FPR for 0.3 s bouts with a mean frequency of 6 Hz for the left-most entry of panel B. The x-axes for panels B through D are bout durations for individual simulated runs of MODAL reported in seconds, and the y-axes are percentages for Sens and FPR. FPR, false positive rate; LFP, local field potential; MODAL, Multiple Oscillation Detection Algorithm; Sens, sensitivity; SNR,signal-to-noise ratio. (TIF) [file pbio.3000546.s010.tif]

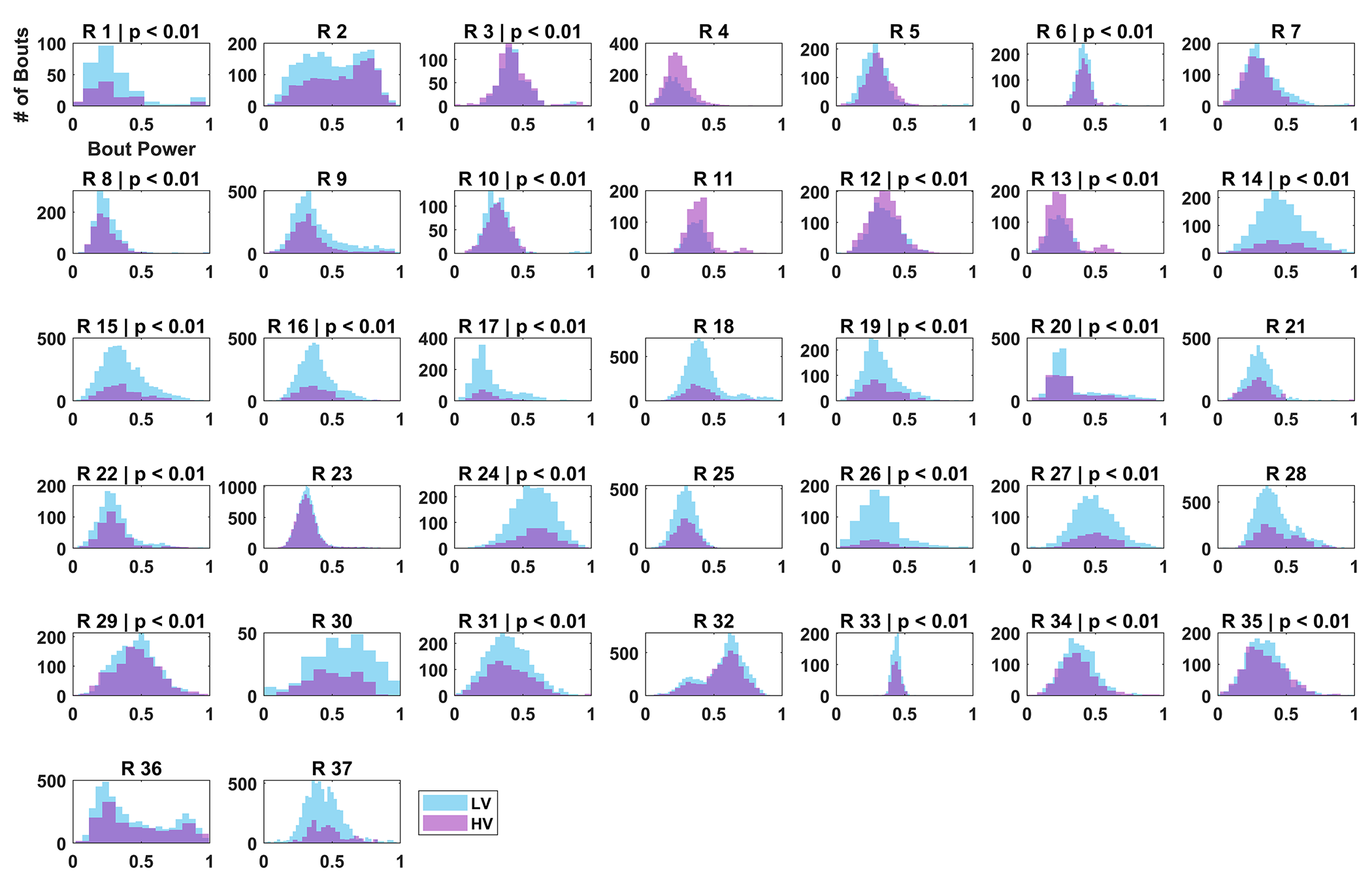

Supplement: S11 Fig — Bout power is plotted on the x-axis in normalized units, and total number of bouts exhibiting each power level by bin is plotted on the y-axis. The p-values reported for each day are indicative of the significance of the difference between the HV and LV duration distributions using the KS-Test. Only p-values that exceeded the α = 0.01 threshold are indicated. HV, high velocity; KS, Kolmogorov-Smirnov Test; LV, low velocity. (TIF) [file pbio.3000546.s011.tif]

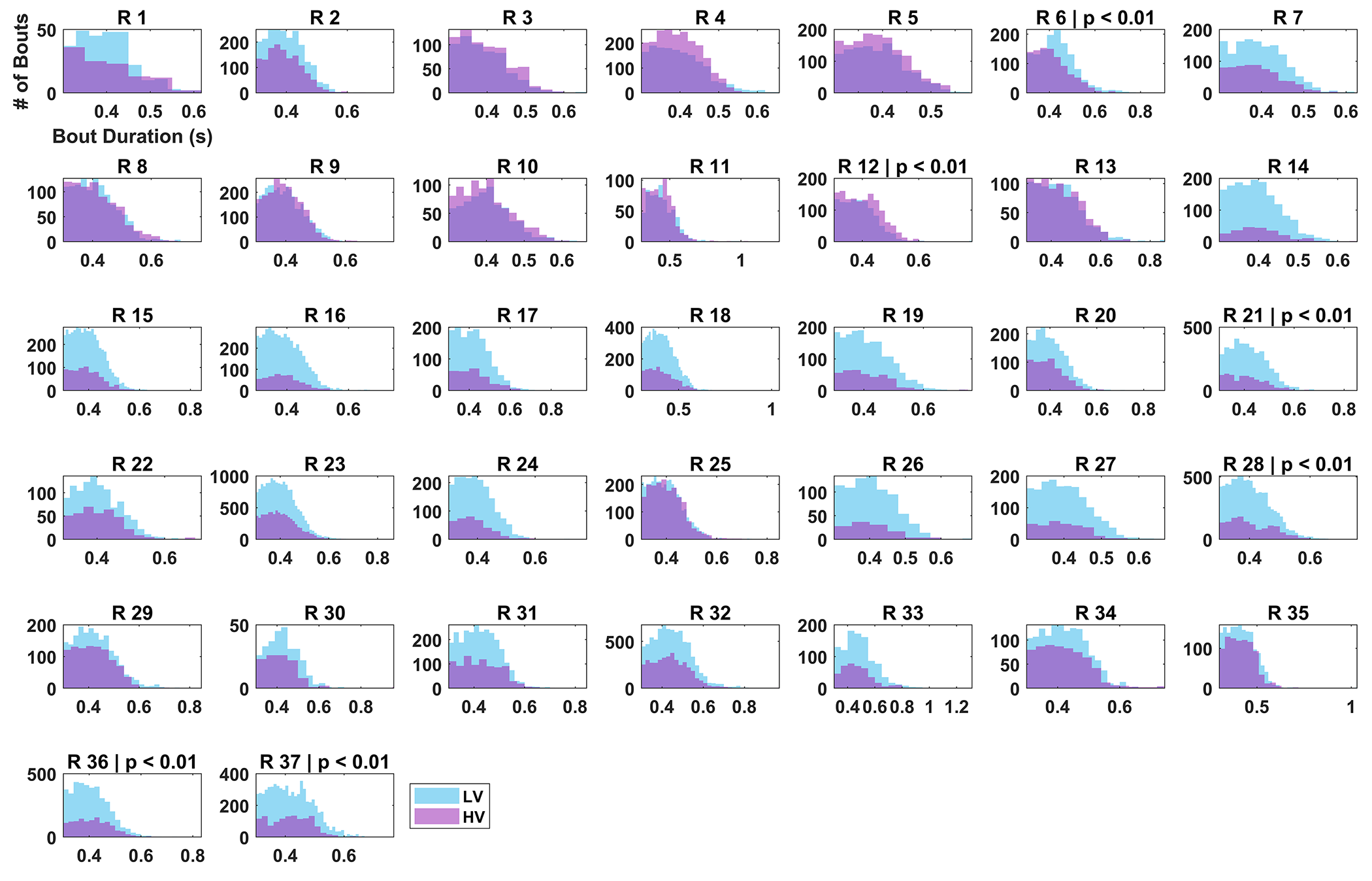

Supplement: S12 Fig — Bout duration is plotted on the x-axis in seconds, and total number of bouts exhibiting each duration by bin is plotted on the y-axis. The p-values reported for each day are indicative of the significance of the difference between the HV and LV duration distributions using the KS-Test. Only p-values that exceeded the α = 0.01 threshold are indicated. HV, high velocity; KS, Kolmogorov-Smirnov Test; LV, low velocity. (TIF) [file pbio.3000546.s012.tif]

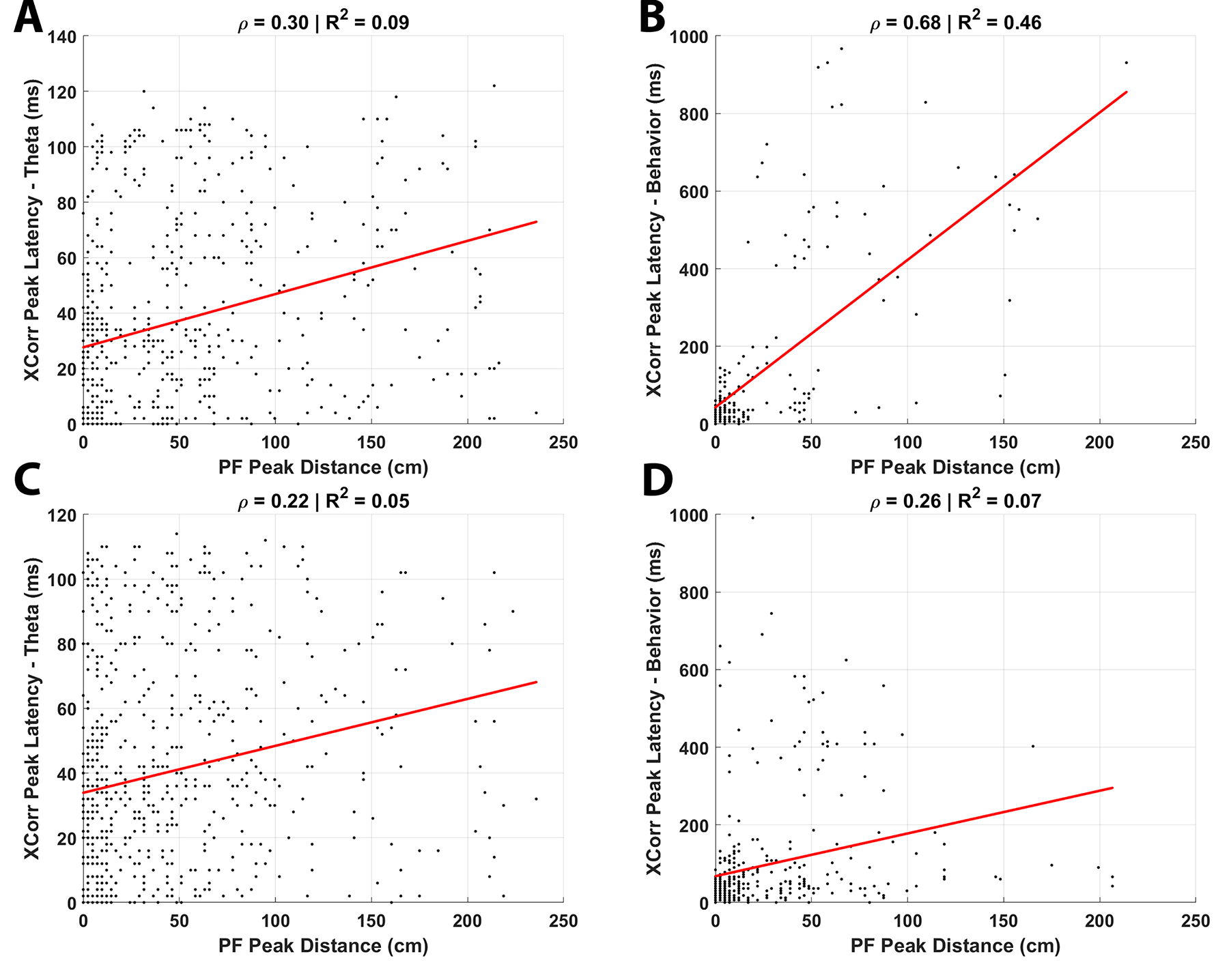

Supplement: S13 Fig — The distance between place-field peaks (x-axis) is plotted against the peak CCG latency for all pairs of units recorded on the same day (y-axis), with each point corresponding to a single place-cell pair. Overlaid red lines indicate the least-square fit of the data with associated Pearson correlation coefficient (ρ) and explained variance (R2) of the fit reported above each plot. (A) Plots of the CCG using the entire recording at the θ time scale. (B) Plots of the CCG using the entire recording at the behavioral time scale. (C) Plots of the CCG construction for the θ time scale but limited to spikes that occurred during θ bouts. (D) Plots of the CCG construction for the behavioral timescale but limited to spikes that occurred during θ bouts. All linear fits computed above were highly statistically significant (p < 10−10 in all cases), but the explained variance for the θ time scale was miniscule (7% = A, 5% = C). CCG, cross-correlogram. (TIF) [file pbio.3000546.s013.tif]

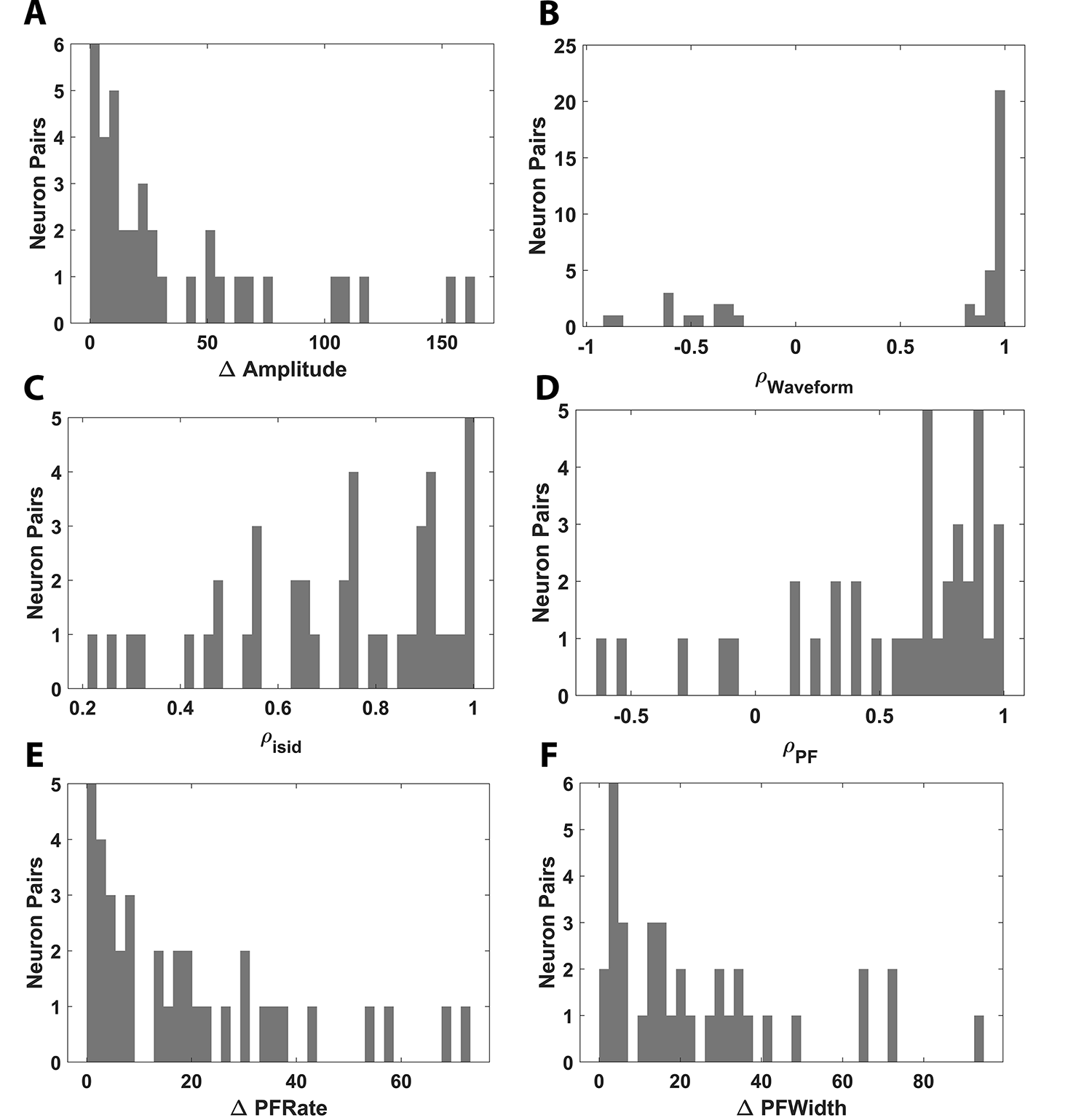

Supplement: S14 Fig — Neuron pairs shown here were on the same channel and exhibited the same place tuning across the different recording sessions. The similarity metrics shown are (A) the change in spike waveform amplitude, (B) the mean waveform correlation between pairs, (C) the correlation of the unsmoothed interspike-interval distribution with 500 ms latency and 5 ms bins. The remaining metrics focused on the place field: (D) place field correlation, (E) the change in peak firing rate within the place field, and (F) the change in width of the place field defined by 20% of max firing rate. For the place field metrics, directionally tuned place fields were matched (i.e., a bidirectionally tuned cell contributed twice to each of these metrics but only once to the metrics shown in panels A through C. MBA, microwire brush array. (TIF) [file pbio.3000546.s014.tif]

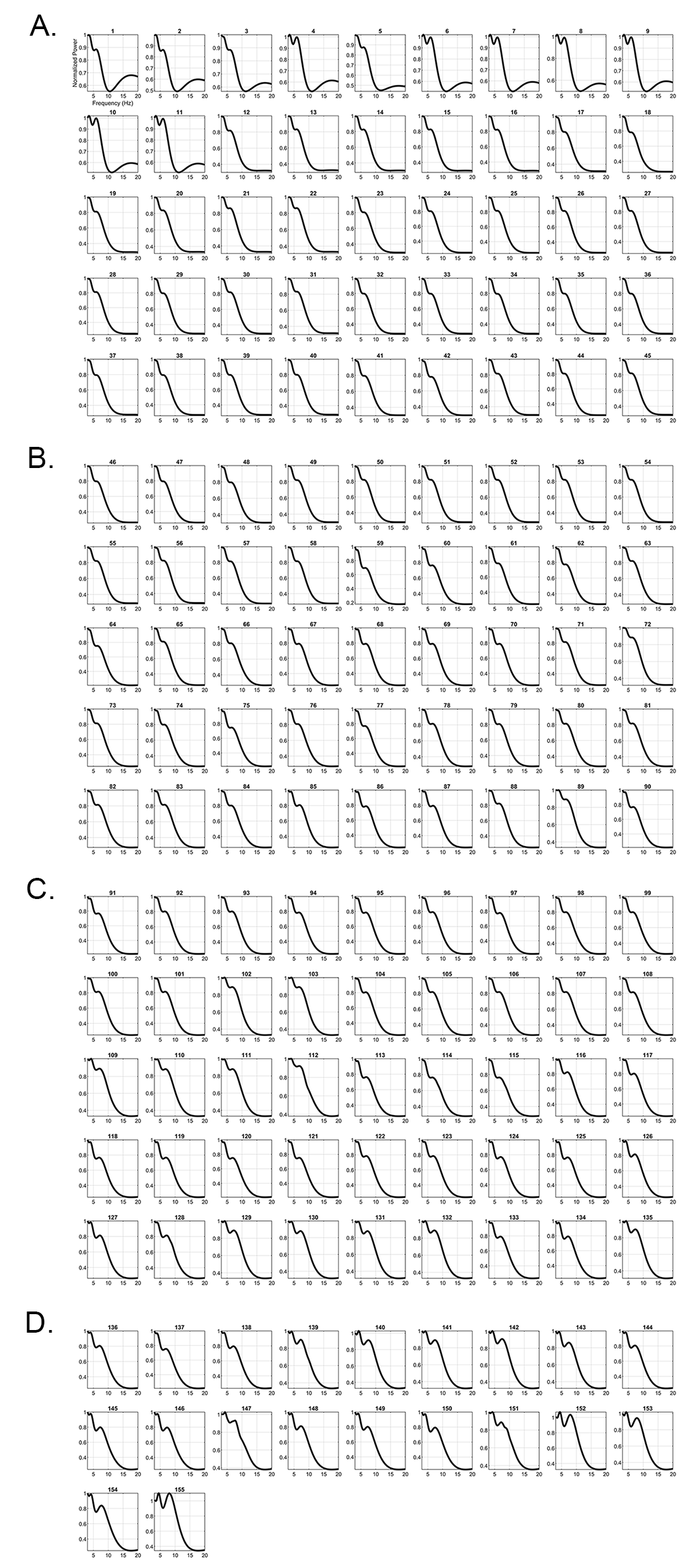

Supplement: S15 Fig — Individual plots are shown with increasing frequency on the x-axis and normalized power on the y-axis. Note spectra in these plots were normalized to the power at the 0Hz frequency of thepower spectrum (DC) to facilitate comparison between channels by visual inspection. The channels here are pooled across all 3 subjects and are sorted in order of increasing peak θ frequency, with the title number being an arbitrary total order for the channels postsorting. The identified θ band was not demarcated to facilitate reading of the estimated spectra but in all cases is centered on the peak consistently found between 5 and 10 Hz across all recordings. (A) Ch 1:45, (B) Ch 46:90, (C) Ch 91:135, (D) Ch 136:155. DC, direct current; MODAL, Multiple Oscillation Detection Algorithm. (TIF) [file pbio.3000546.s015.tif]

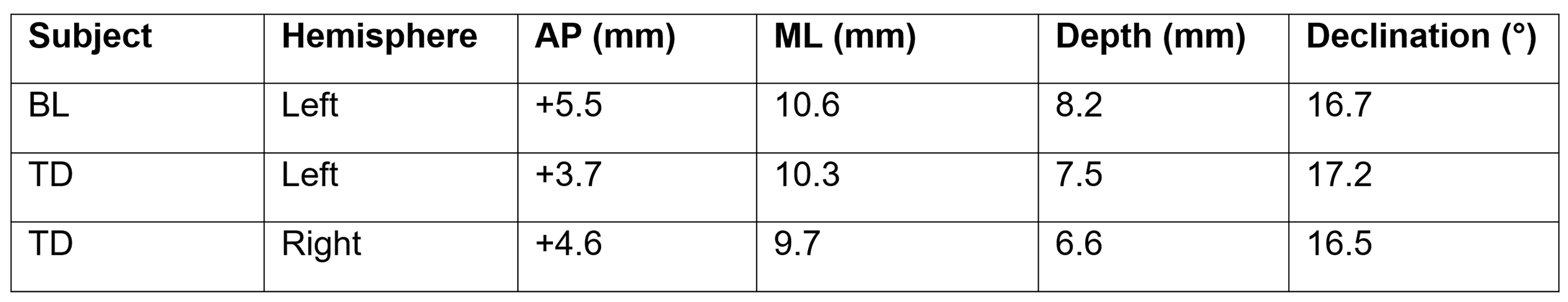

Supplement: S1 Table — The AP axis is computed with respect to the center of the interaural canal, which was clearly identifiable in all pre- and postoperative MRIs. The medio-lateral axis distance is computed with respect to the cerebral fissure. The declination is reported as angular deviation in degrees from true vertical. Depth is computed as linear distance advanced from the surface of cortex to the end of the leading edge of the guide tube within hippocampus. Note that, during postoperative scanning, the MBA had already been advanced 250 μm beyond the leading edge, though splaying of the individual electrode tips cannot be visualized because of isotropic spatial resolution limitations. AP, Anterior Posterior; MBA, microwire brush array. (TIF) [file pbio.3000546.s016.tif]

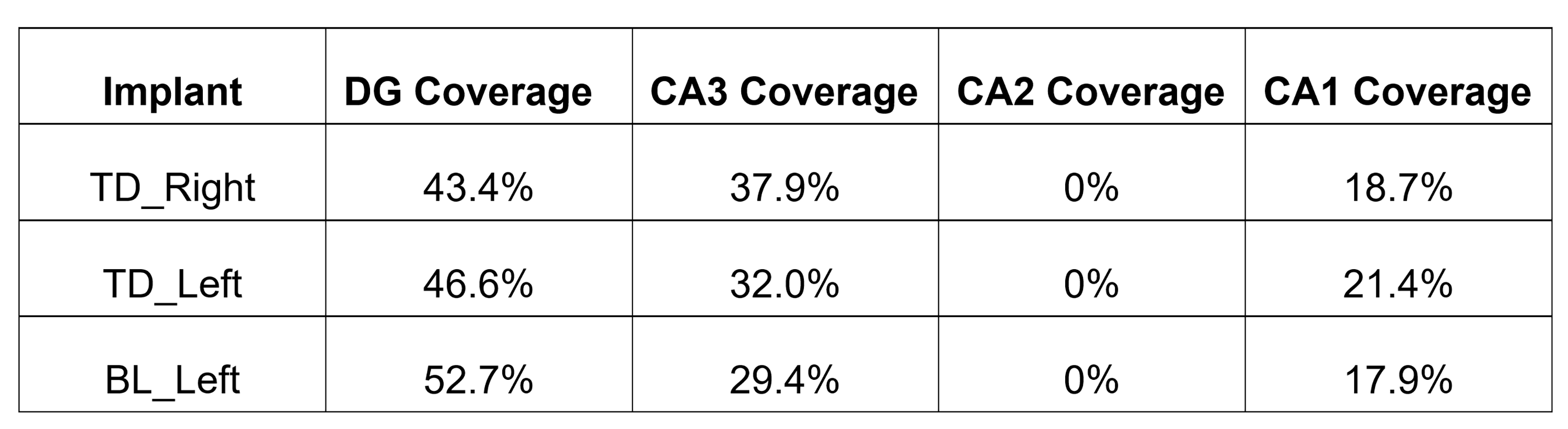

Supplement: S2 Table — The first column indicates the identity of each implant ordered from posterior to anterior, and the associated row indicates the fraction of pyramidal/granular layer voxels within hippocampus that the implant contains. Percentages for each implant are computed separately based on number of voxels falling within the pyramidal layer of each CA field and the granular layer of the dentate gyrus divided by the total number of voxels from all CA-field pyramidal layers within the hippocampus as indicated by the Riken T2-weighted anatomical atlas aligned with the included NISSL stain. CA, cornu ammonis. (TIF) [file pbio.3000546.s017.tif]

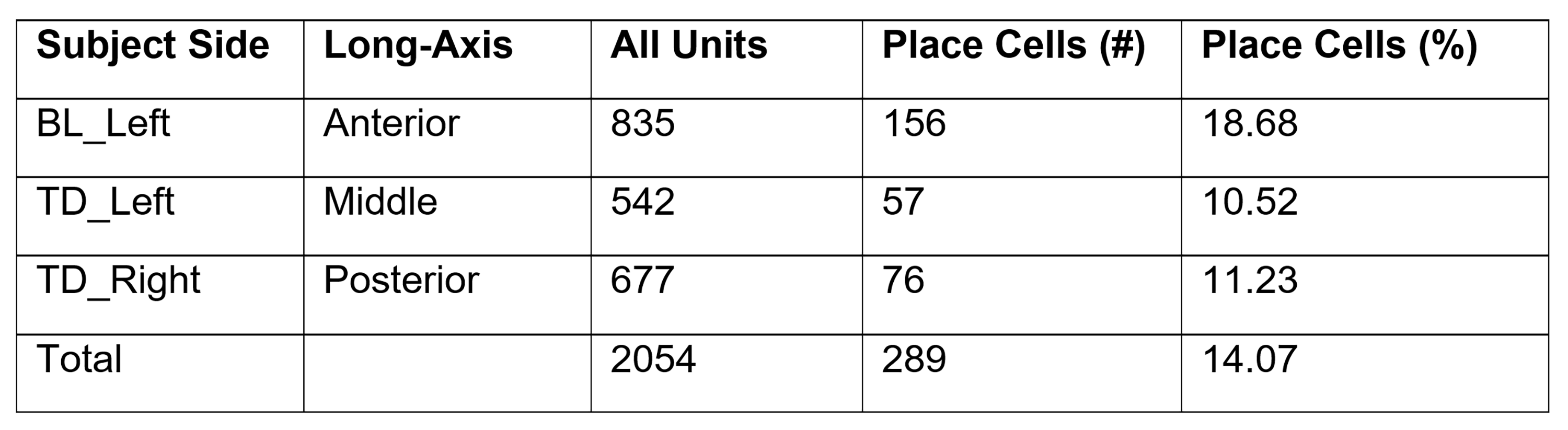

Supplement: S3 Table — The implants are reported in order from anterior to posterior according to their associated ROI’s projection onto the hippocampal long axis (see S8 Fig for details). All of the units recorded from a given MBA implant site are reported in the “All Units” column. The absolute number of place cells at the same location is reported in the “Place Cells (#)” column, and the ratio of the 2 is reported in the “Place Cells (%)” column. It is noted that for these counts, units intentionally recorded on the same day for stability analyses were included but not double or triple counted across the repeated sessions. MBA, microwire brush array; ROI, region of interest. (TIF) [file pbio.3000546.s018.tif]
